# Supplementary material for: Serum N-Glycomics Stratifies Bacteremic Patients Infected with Different Pathogens
Source: J Clin Med. 2021 Feb 1;10(3):516. doi: 10.3390/jcm10030516 (PMC7867038; doi:10.3390/jcm10030516)
Supplement: Supplementary file 1 [file jcm-10-00516-s001.zip › Chatterjee_Bacteremia_JClinMed_Revised_Supplementary Data S1-S3.pdf]

Supplementary Data S1-S3

for

Serum *N*-Glycomics Stratifies Bacteremic Patients Infected with Different Pathogens

Sayantani Chatterjee [1,2], Rebeca Kawahara [1,2], Harry C. Tjondro [1,2], David R. Shaw [3], Marni Nenke [4,5,6], David J. Torpy [4,5], Morten Thaysen-Andersen [1,2]*

[1]*Department of Molecular Sciences, Macquarie University, Sydney, Australia*

[2]*Biomolecular Discovery Research Centre, Macquarie University, Sydney, NSW, Australia*

[3]*Infectious Diseases Clinic, Royal Adelaide Hospital, Adelaide, Australia*

[4]*Endocrine and Metabolic Unit, Royal Adelaide Hospital, Adelaide, Australia*

[5]*School of Medicine, University of Adelaide, Adelaide, Australia*

[6]*Department of Endocrinology and Diabetes, The Queen Elizabeth Hospital, Woodville South, Australia*

***Corresponding author**:
Dr Morten Thaysen-Andersen, PhD
Department of Molecular Sciences
Macquarie University, NSW-2109, Sydney, Australia
Ph: +61 2 9850 7487
Email: morten.andersen@mq.edu.au

Overview of Supplementary Data

Annotation and Fragmentation Key (slide 3)

Supplementary Data S1: BPC, EICs and manually annotated PGC-LC-ESI-CID-MS (-) spectra of *N*-glycans (alditols) of key importance released from bacteremic sera (Slide 4 – Slide 19).

Supplementary Data S2: Examples of EICs, S/N and manually annotated PGC-LC-ESI-CID-MS (-) spectra of very low abundant *N*-glycans (alditols) released from bacteremic sera (0.01%-0.15% relative abundance levels) (Slide 20 – Slide 25).

Supplementary Data S3: Manually annotated PGC-LC-ESI-CID-MS/MS (-) spectra of all reported *N*-glycans (alditols) released from bacteremic and healthy sera (Slide 26 – Slide 88).

Annotation and Fragmentation Key

- Mannose (Man) (162.0528 Da)
- Galactose (Gal) (162.0528 Da)
- *N*-Acetylglucosamine (GlcNAc) (203.0794 Da)
- *N*-Acetylneuraminic acid (NeuAc) (291.0954 Da)
- Fucose (Fuc) (146.0579 Da)
- Cross-ring fragment (unspecified)
- Indicates mostly Y ions (includes oxygen of glyosidic linkage)
- Indicates mostly Z ions (excludes oxygen of glyosidic linkage)
- Reduced reducing end

a-b-c-d Isomers

Unknown linkage

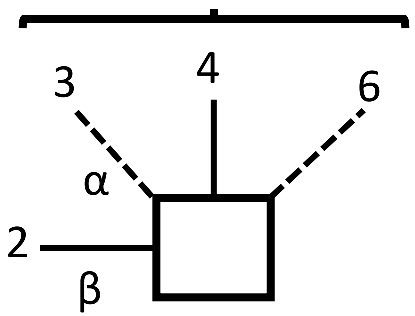

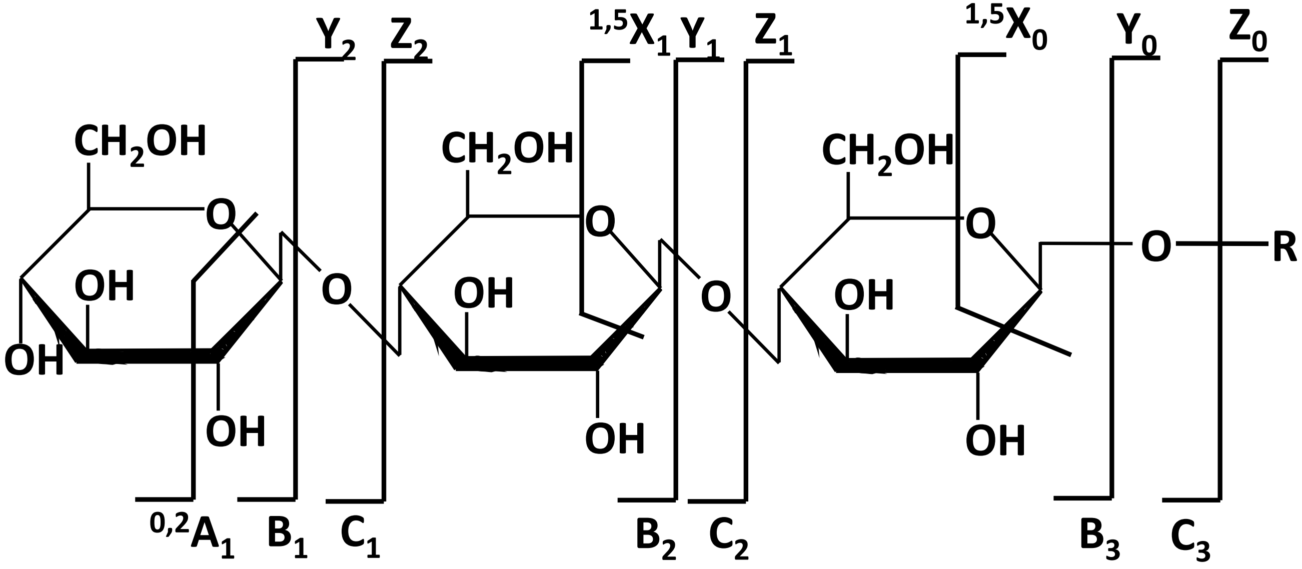

Han, L. & Costello, C.E. Mass spectrometry of glycans. *Biochemistry (Moscow)* 78, 710-720 (2013)

Supplementary Data S1

BPC, EICs and manually annotated PGC-LC-ESI-CID-MS (-) spectra of *N*-glycans (alditols) of key importance released from bacteremic sera

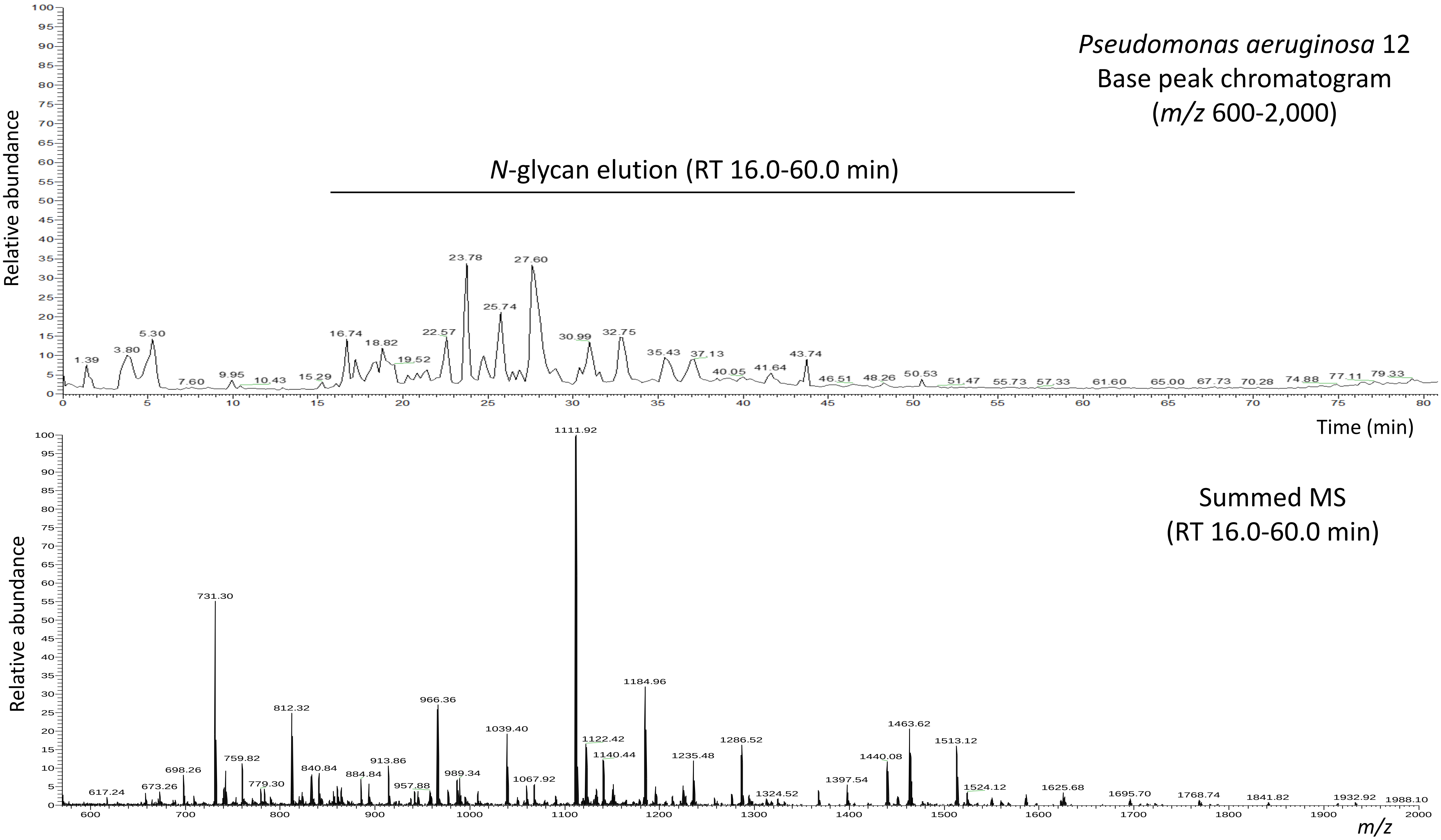

Pseudomonas aeruginosa 12
Base peak chromatogram
(m/z 600-2,000)
N-glycan elution (RT 16.0-60.0 min)
Relative abundance
Time (min)
1.39
3.80
5.30
7.60
9.95
10.43
15.29
16.74
18.82
19.52
22.57
23.78
25.74
27.60
30.99
32.75
35.43
37.13
40.05
41.64
43.74
46.51
48.26
50.53
51.47
55.73
57.33
61.60
65.00
67.73
70.28
74.88
77.11
79.33
Summed MS
(RT 16.0-60.0 min)
Relative abundance
m/z
617.24
673.26
698.26
731.30
759.82
779.30
812.32
840.84
884.84
913.86
957.88
966.36
989.34
1039.40
1067.92
1111.92
1122.42
1140.44
1184.96
1235.48
1286.52
1324.52
1397.54
1440.08
1463.62
1513.12
1524.12
1625.68
1695.70
1768.74
1841.82
1932.92
1988.10

Escherichia coli 6
Extracted ion chromatogram
(*m/z* 799.82)

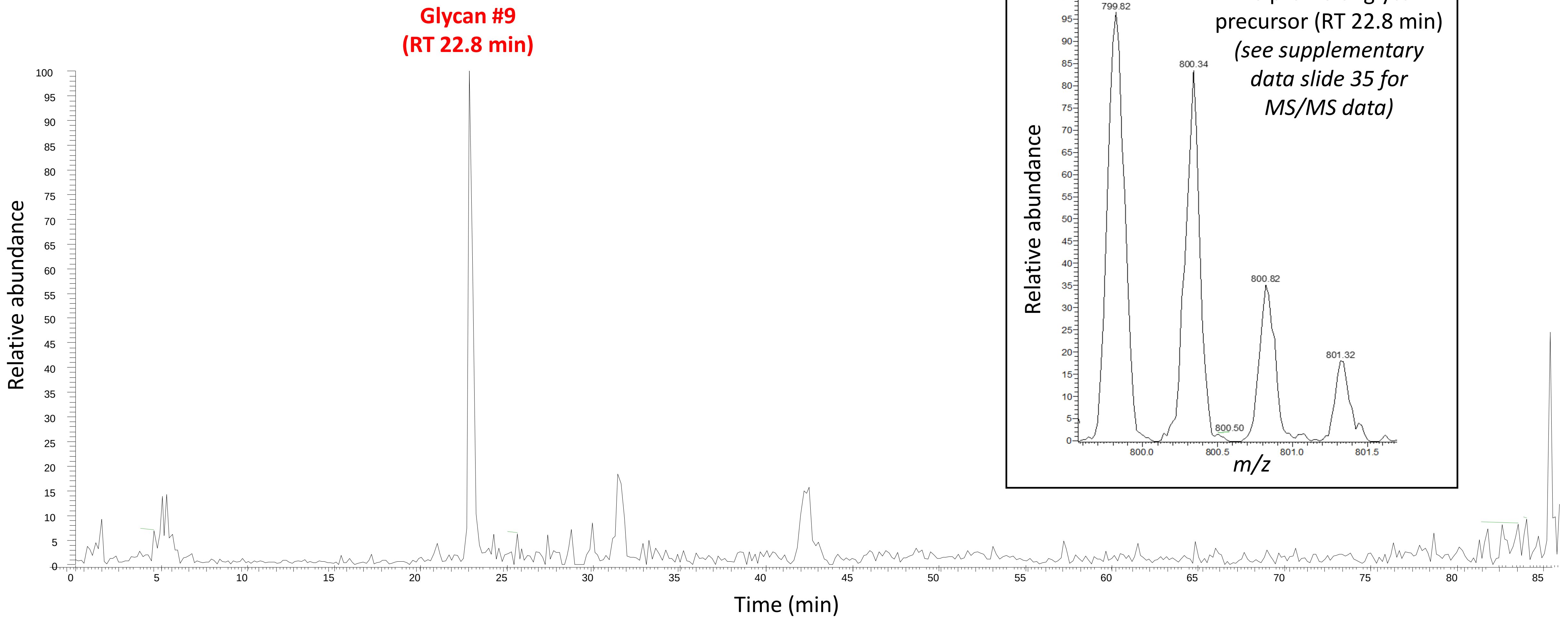

***Monoisotopic signal of identified glycan**

Pseudomonas aeruginosa 12
Extracted Ion Chromatogram
(*m/z* 1260.48)

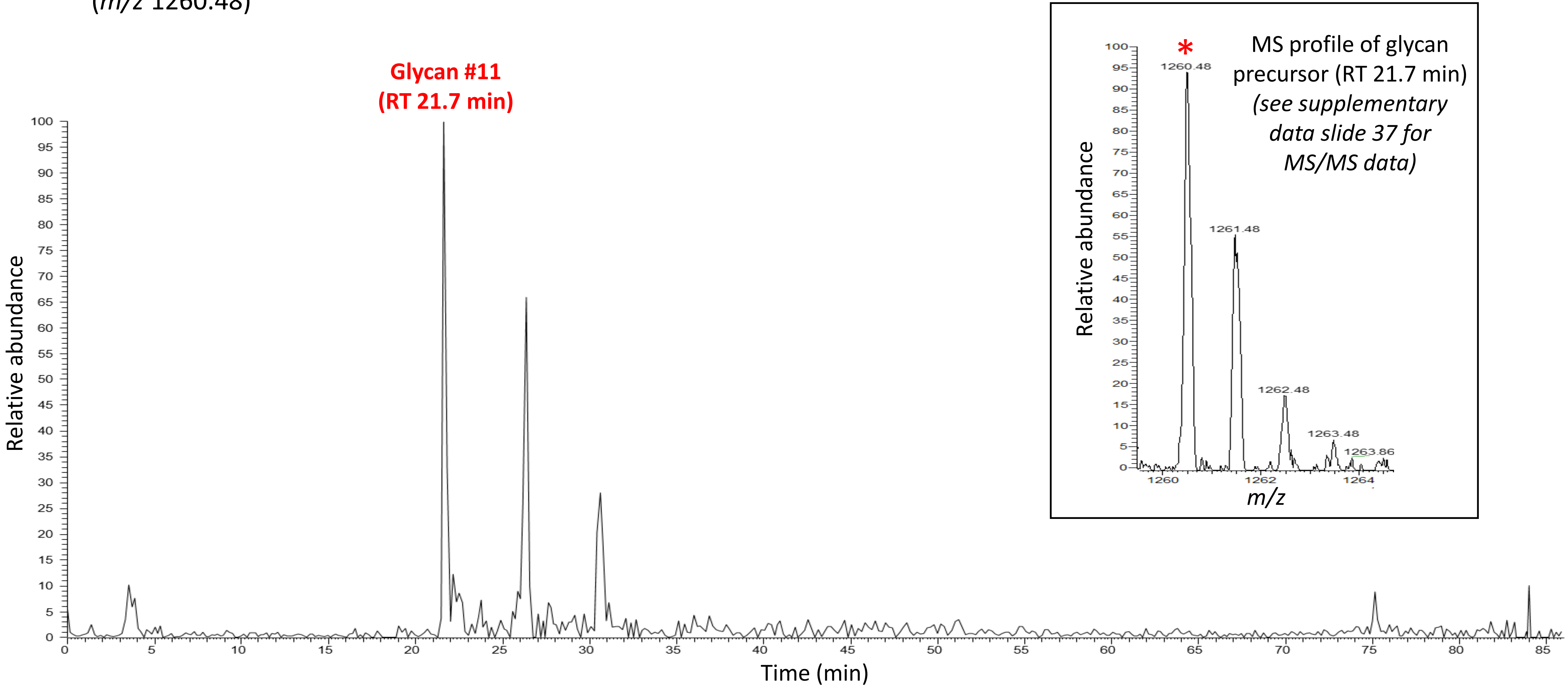

*Monoisotopic signal of identified glycan

Staphylococcus aureus 19
Extracted Ion Chromatogram
m/z 783.32

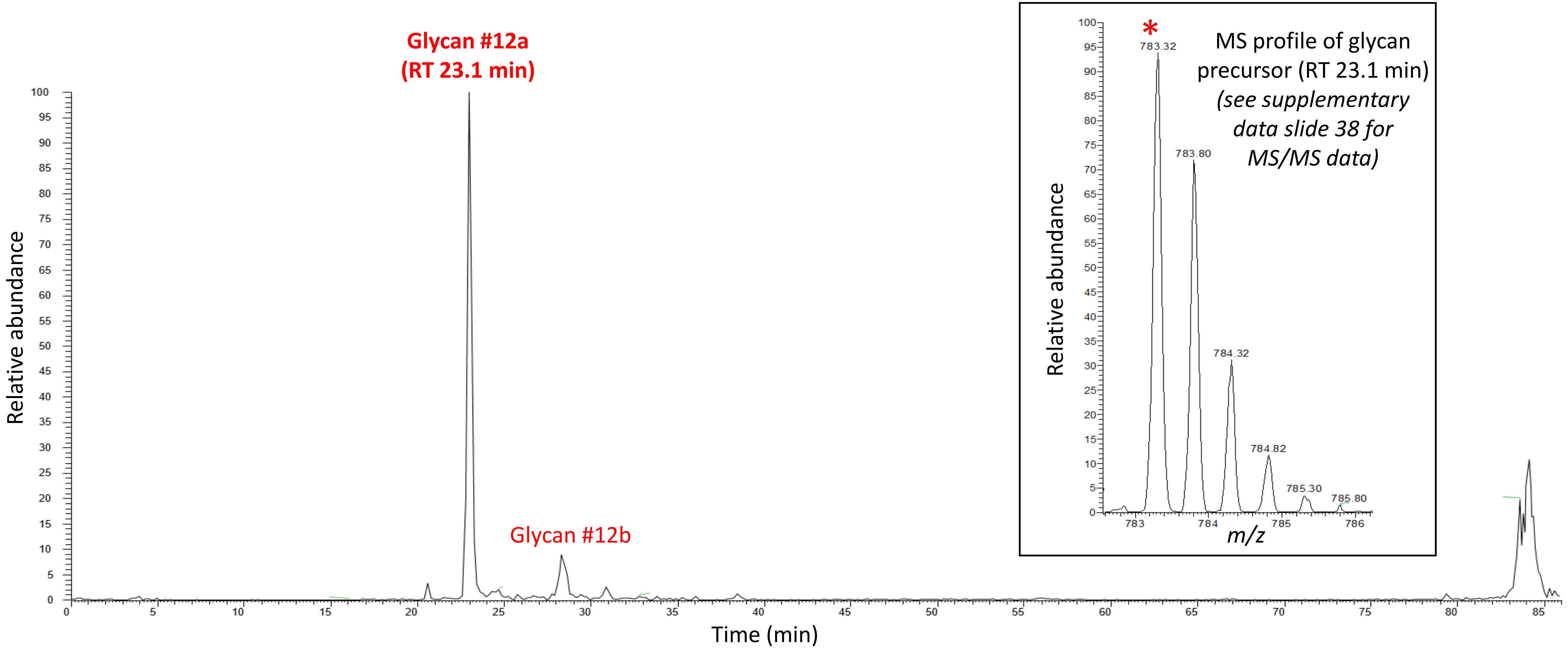

***Monoisotopic signal of identified glycan**

Streptococcus viridans 29
Extracted Ion Chromatogram
m/z 1512.68

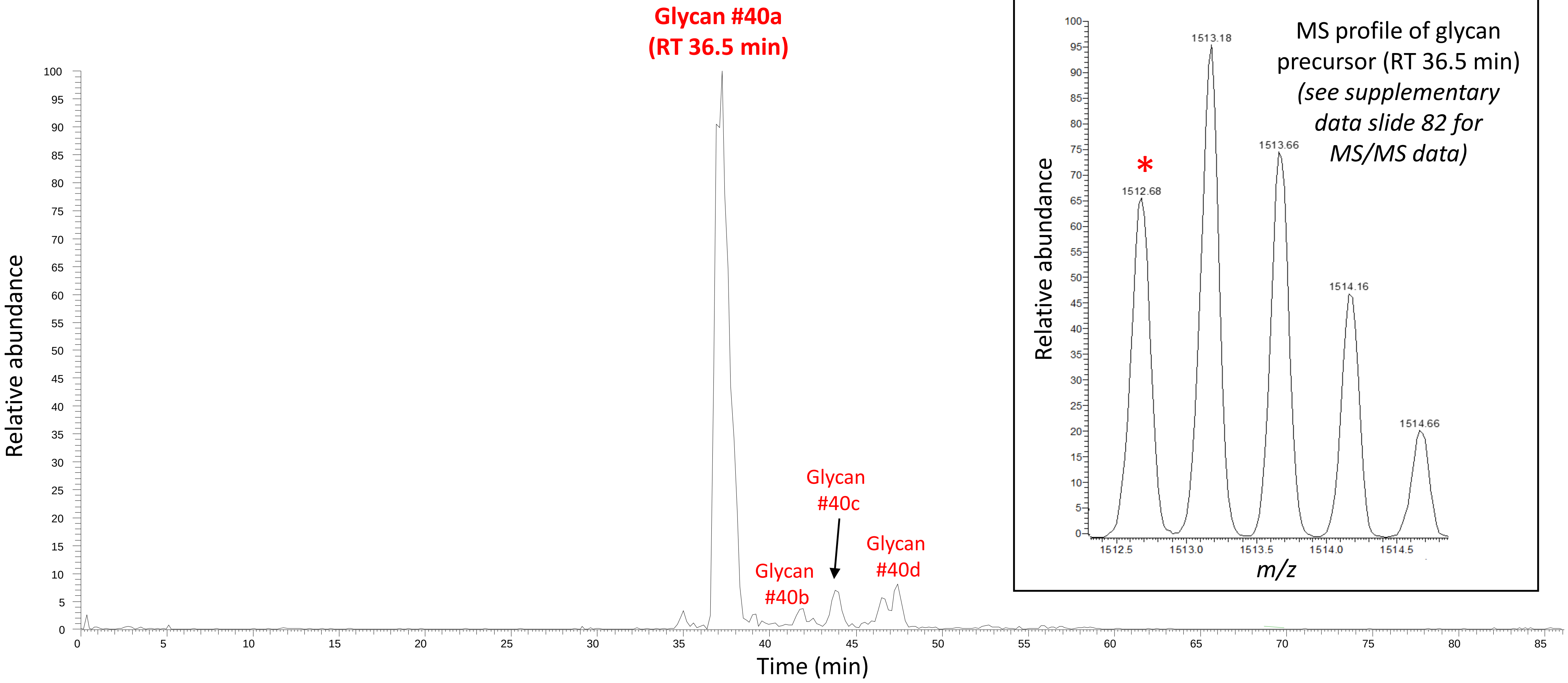

*Monoisotopic signal of identified glycan

Pseudomonas aeruginosa 12
Extracted Ion Chromatogram
m/z 832.82

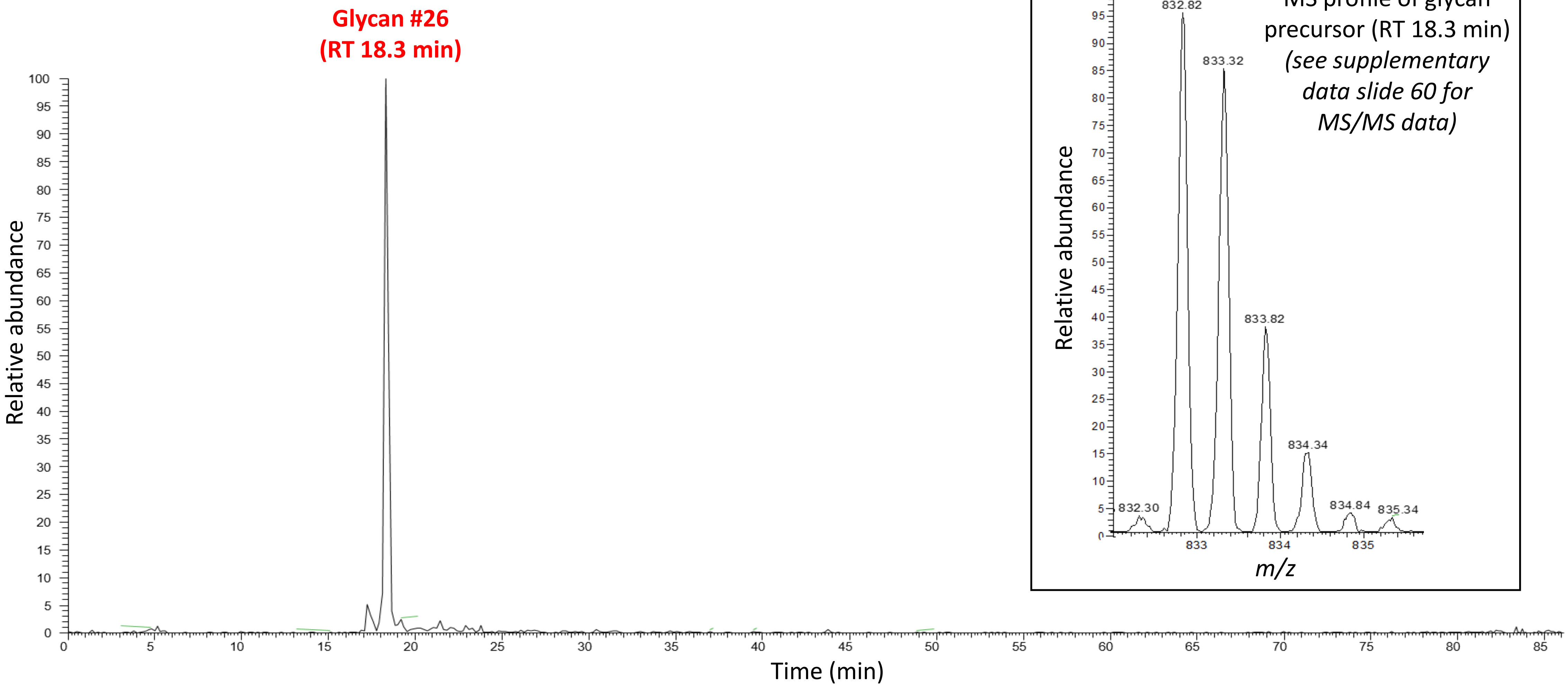

*Monoisotopic signal of identified glycan

Pseudomonas aeruginosa 12
Extracted Ion Chromatogram
m/z 739.30

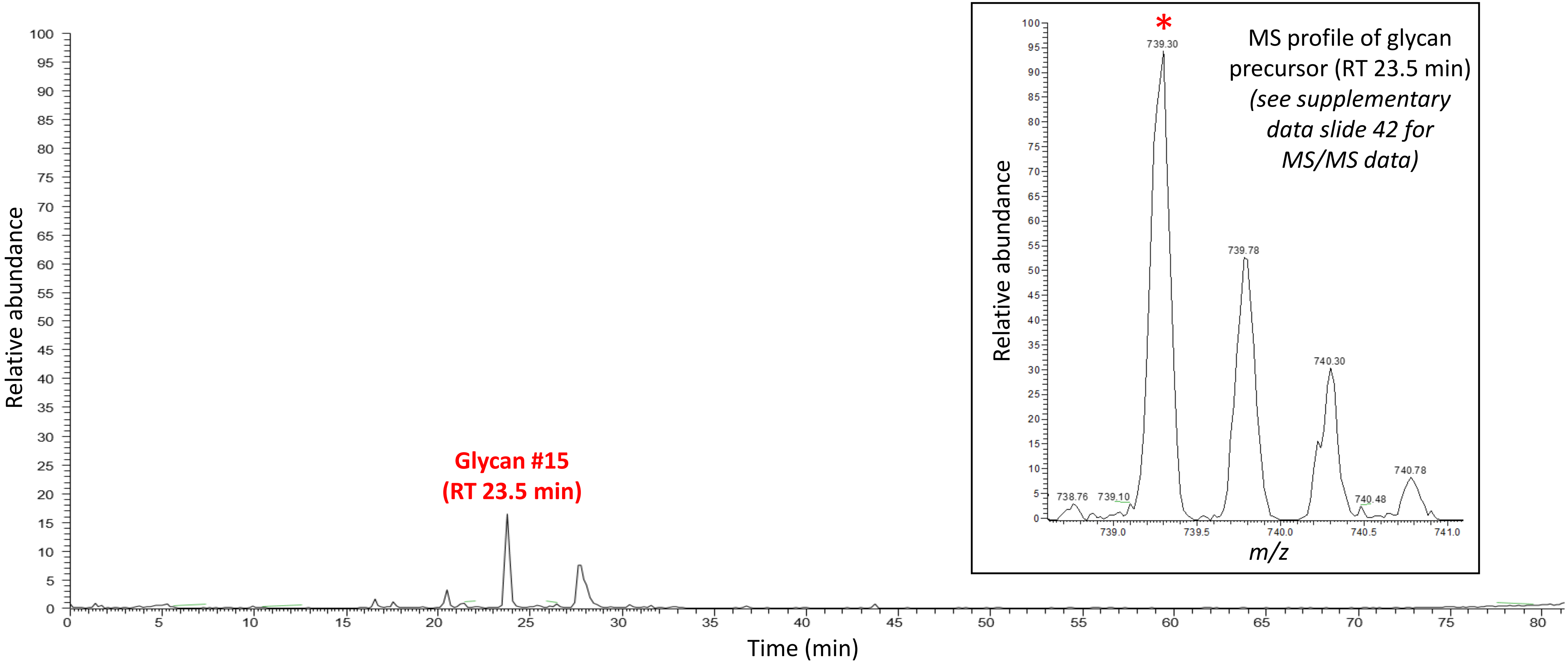

*Monoisotopic signal of identified glycan

Pseudomonas aeruginosa 12
Extracted Ion Chromatogram
m/z 1059.42

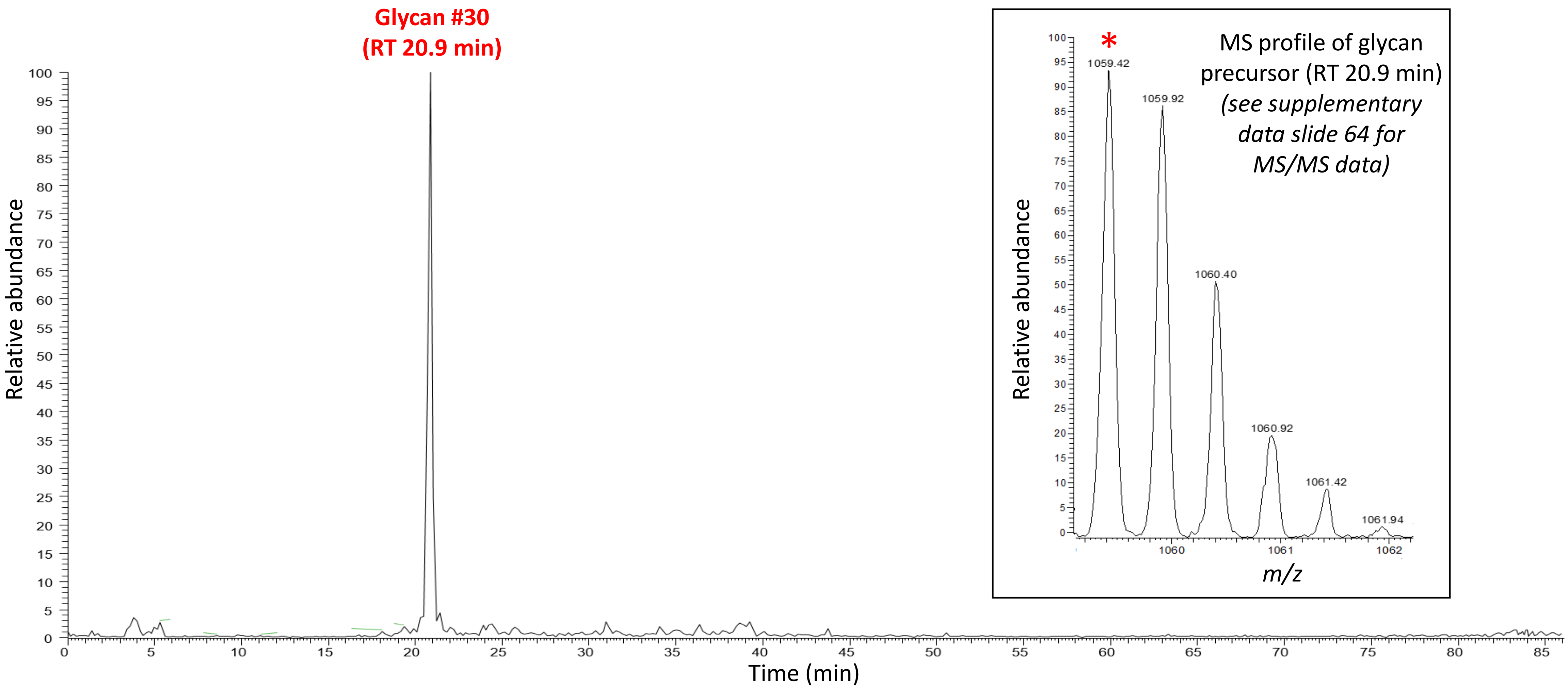

***Monoisotopic signal of identified glycan**

Pseudomonas aeruginosa 12
Extracted Ion Chromatogram
m/z 731.30

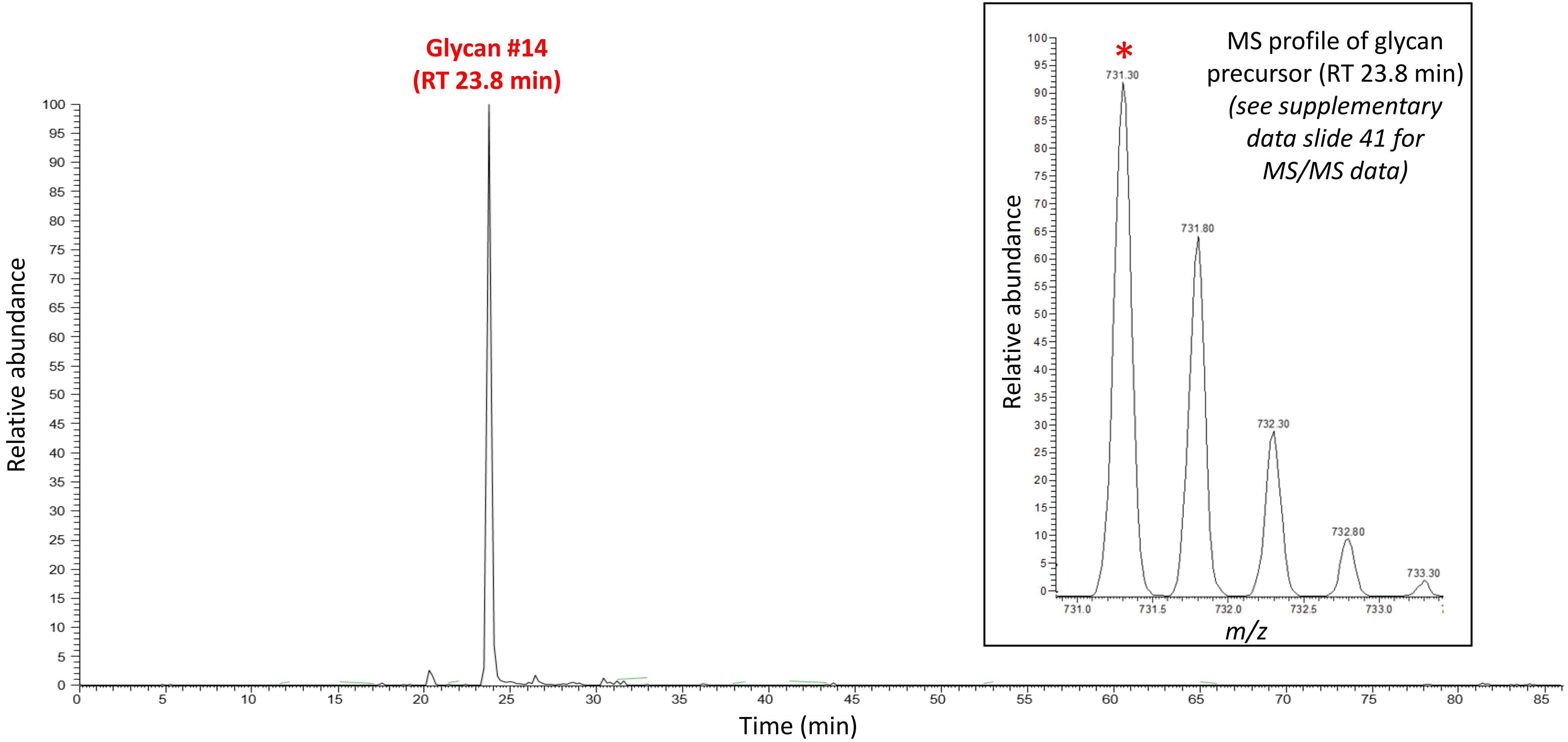

*Monoisotopic signal of identified glycan

Pseudomonas aeruginosa 12
Extracted Ion Chromatogram
m/z 1038.92

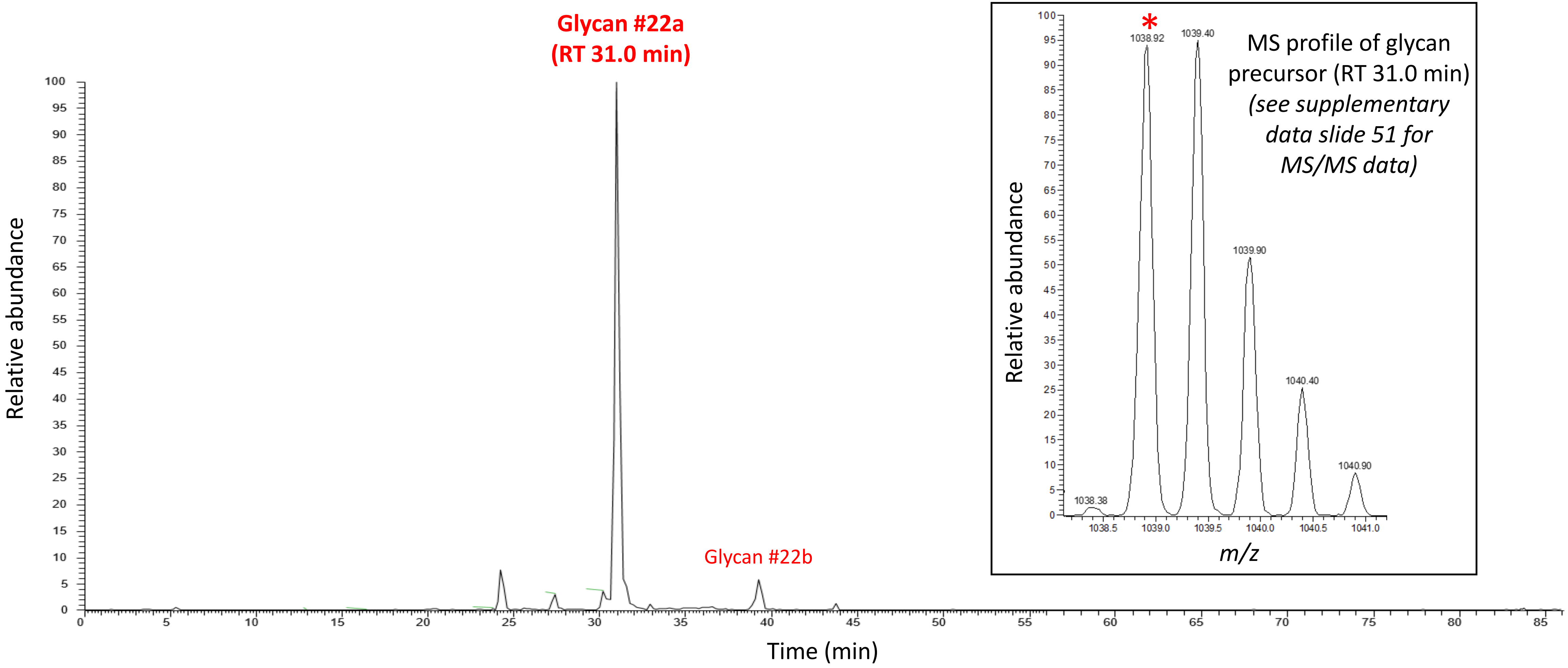

***Monoisotopic signal of identified glycan**

Pseudomonas aeruginosa 12
Extracted Ion Chromatogram
m/z 913.88

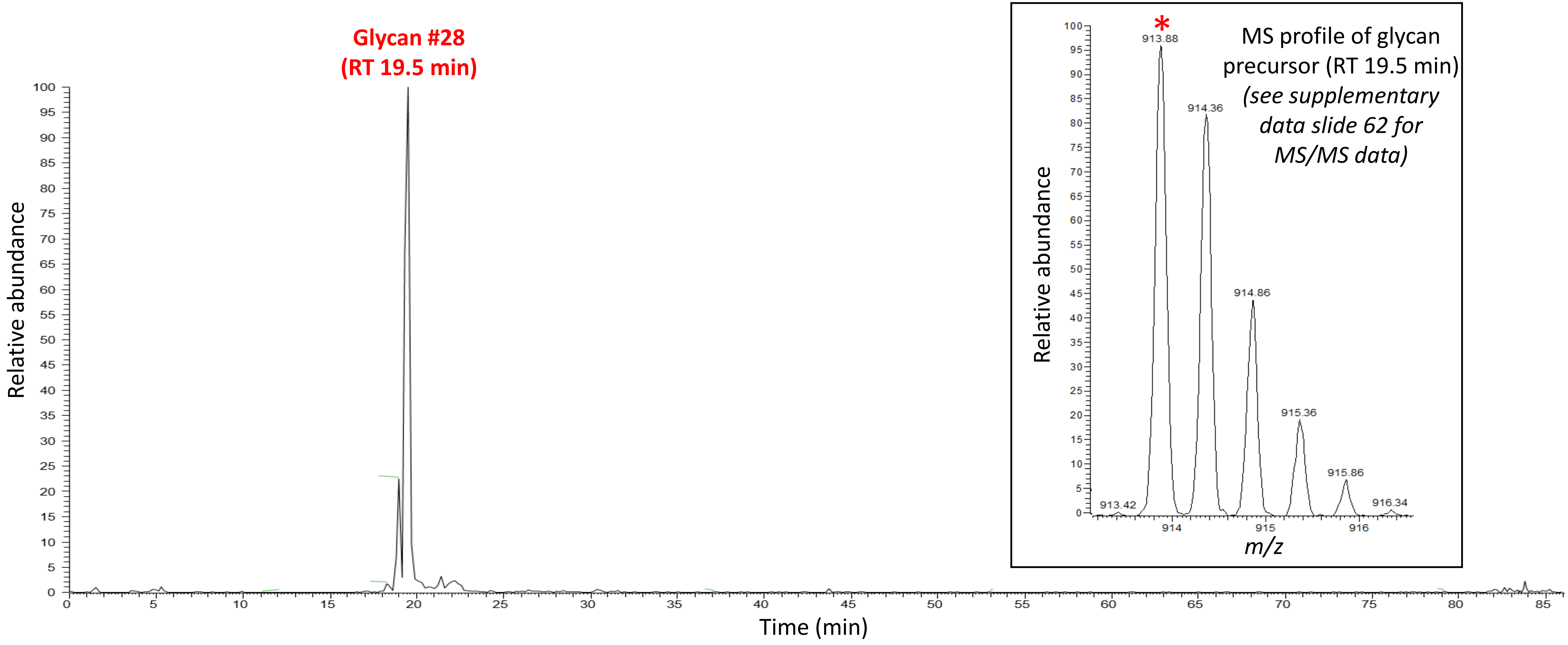

*Monoisotopic signal of identified glycan

Pseudomonas aeruginosa 12
Extracted Ion Chromatogram
m/z 812.32

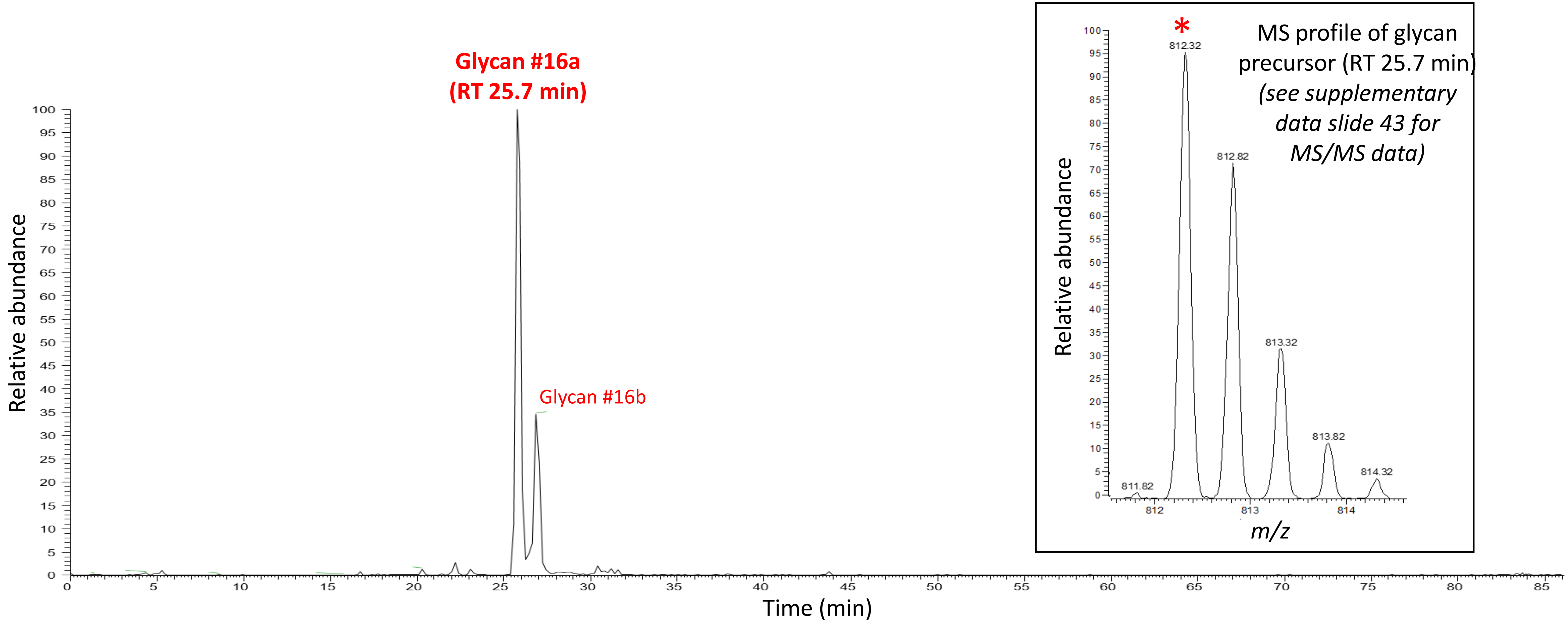

***Monoisotopic signal of identified glycan**

Pseudomonas aeruginosa 12
Extracted Ion Chromatogram
m/z 893.36

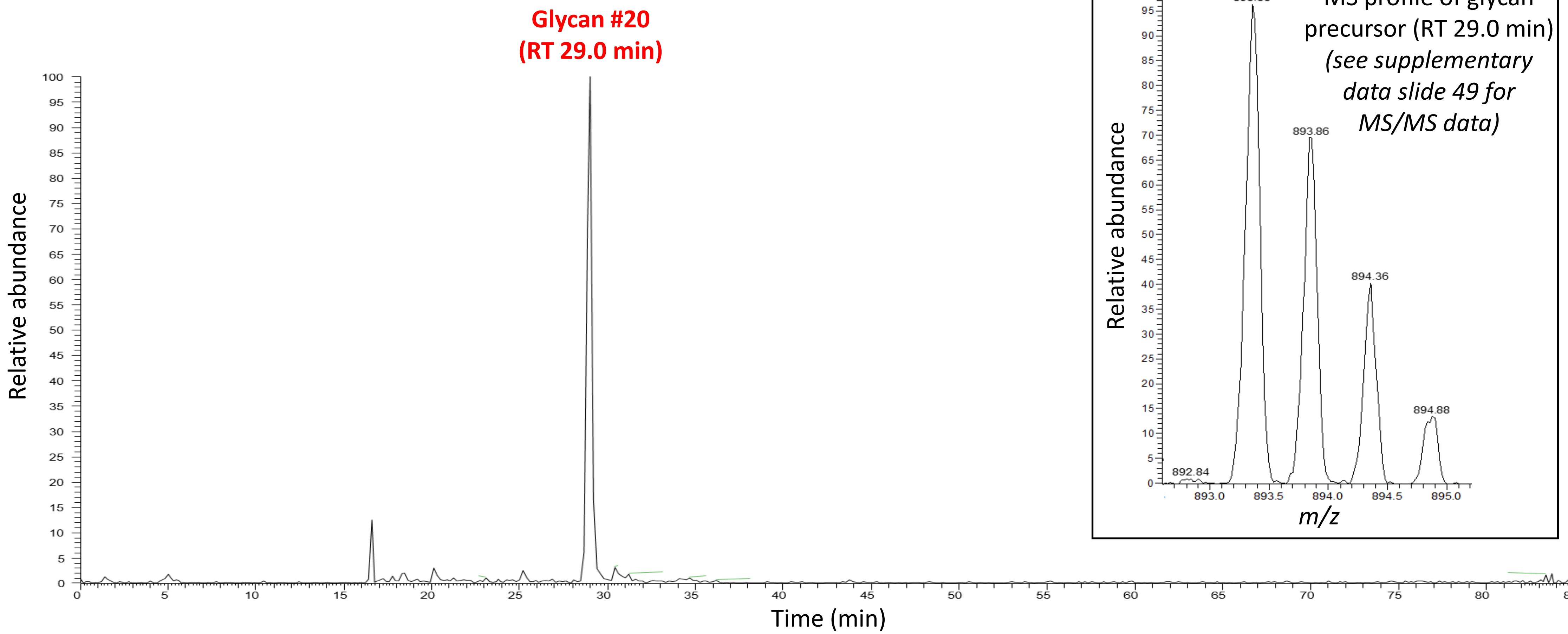

*Monoisotopic signal of identified glycan

Pseudomonas aeruginosa 12
Extracted Ion Chromatogram
m/z 812.32

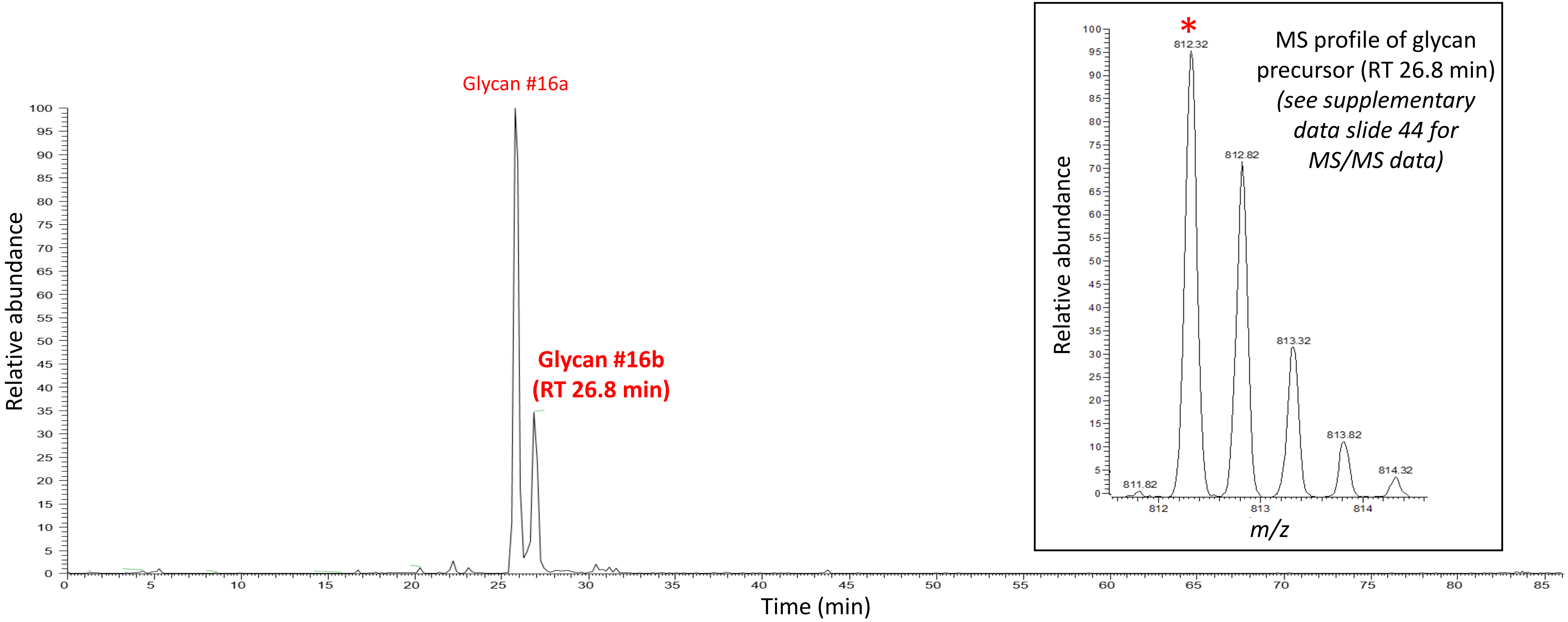

*Monoisotopic signal of identified glycan

Pseudomonas aeruginosa 12
Extracted Ion Chromatogram
m/z 957.88

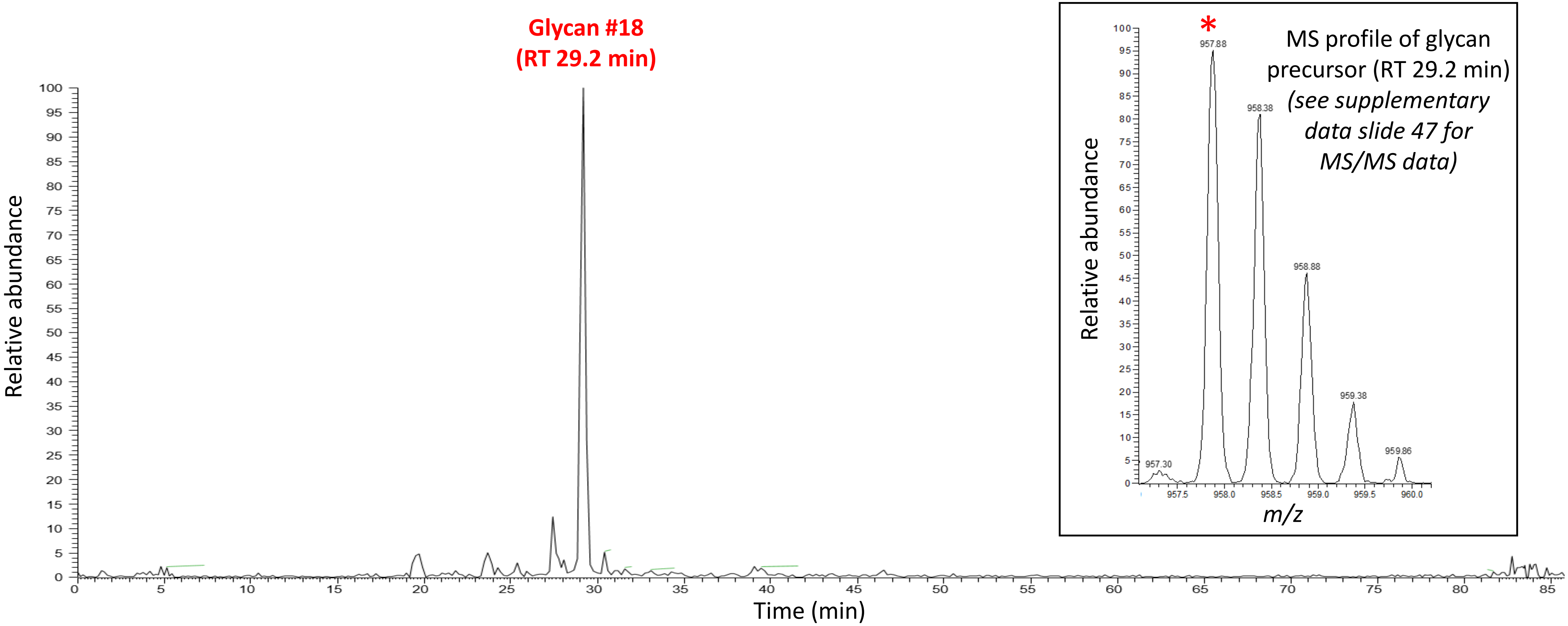

*Monoisotopic signal of identified glycan

Supplementary Data S2

Examples of EICs, S/N and manually annotated PGC-LC-ESI-CID-MS (-) spectra of very low abundant *N*-glycans (alditols) released from bacteremic sera (0.01%-0.15% relative abundance levels)

Escherichia coli 8
Extracted Ion Chromatogram
m/z 1022.42

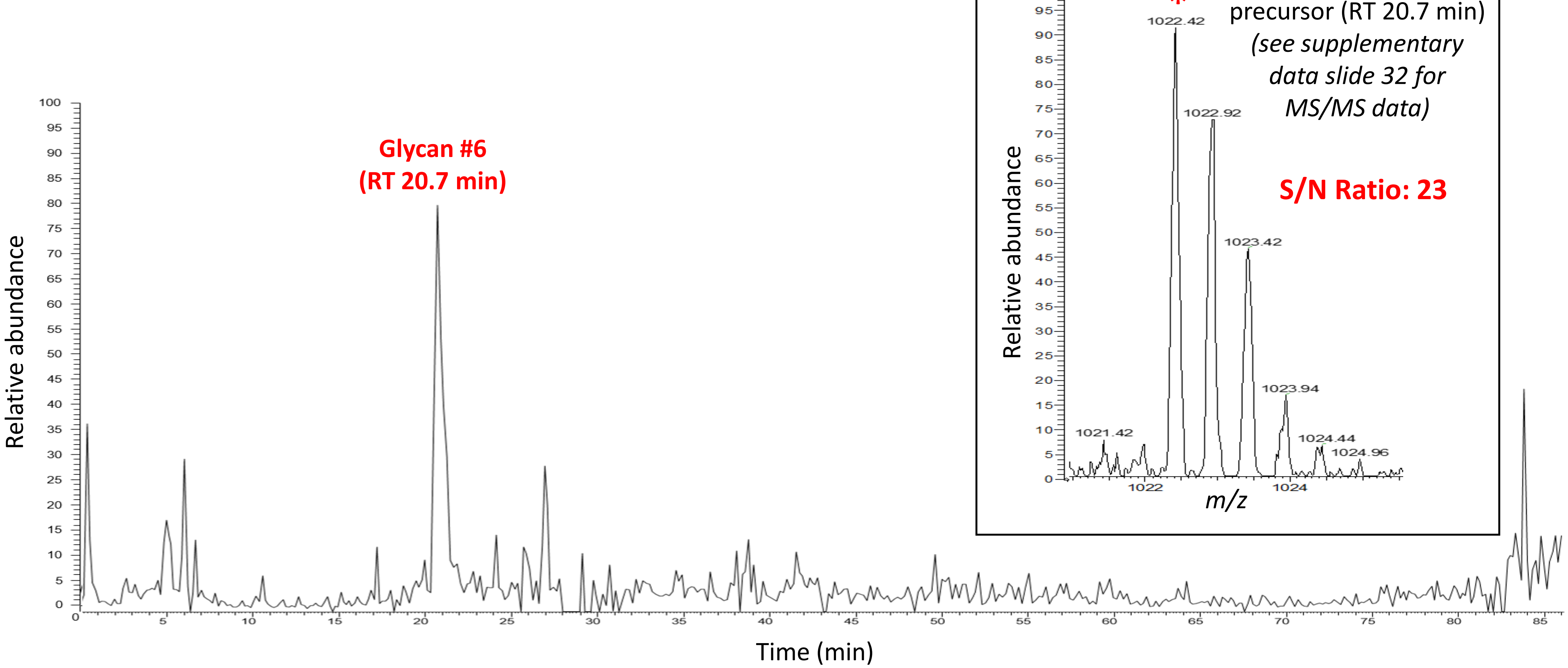

*Monoisotopic signal of identified glycan

Staphylococcus aureus 17
Extracted Ion Chromatogram
m/z 718.76

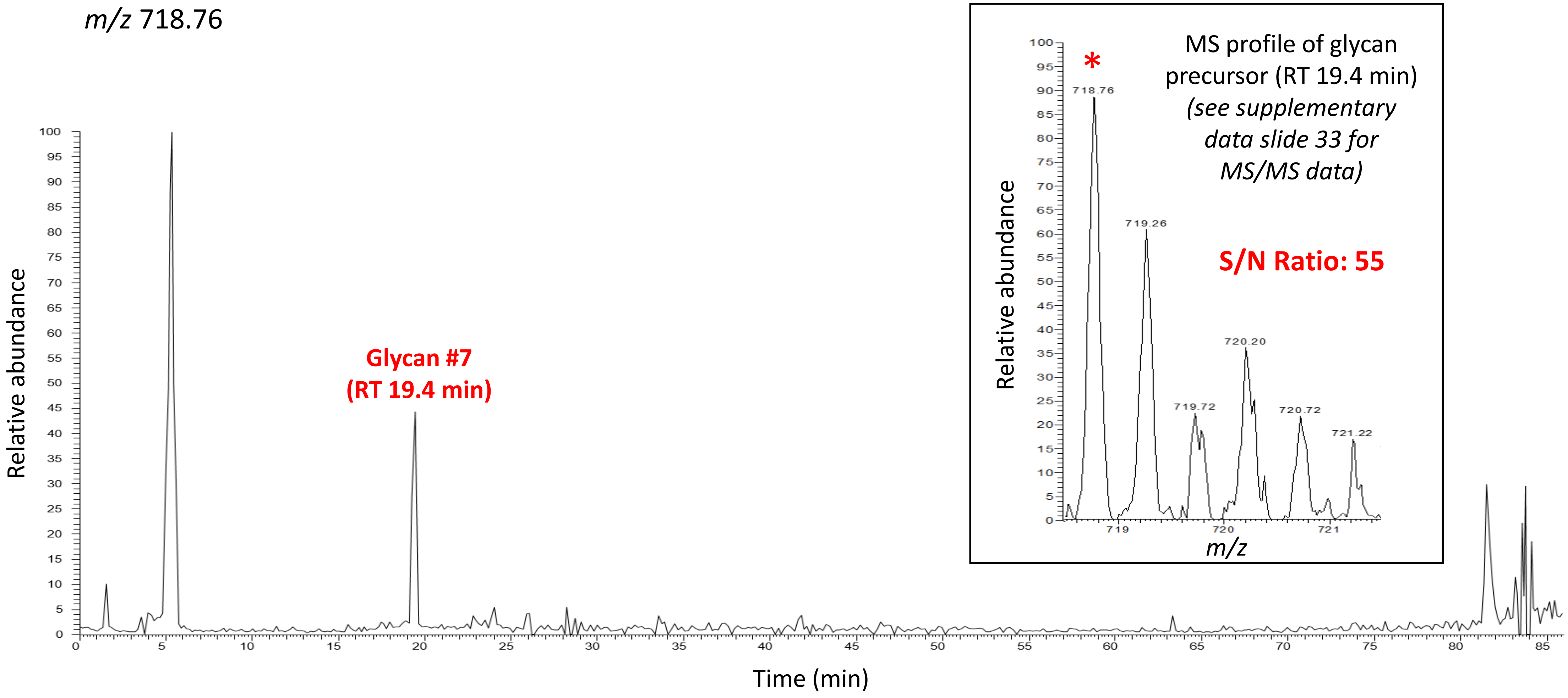

*Monoisotopic signal of identified glycan

Streptococcus viridans 29
Extracted Ion Chromatogram
m/z 1213.00

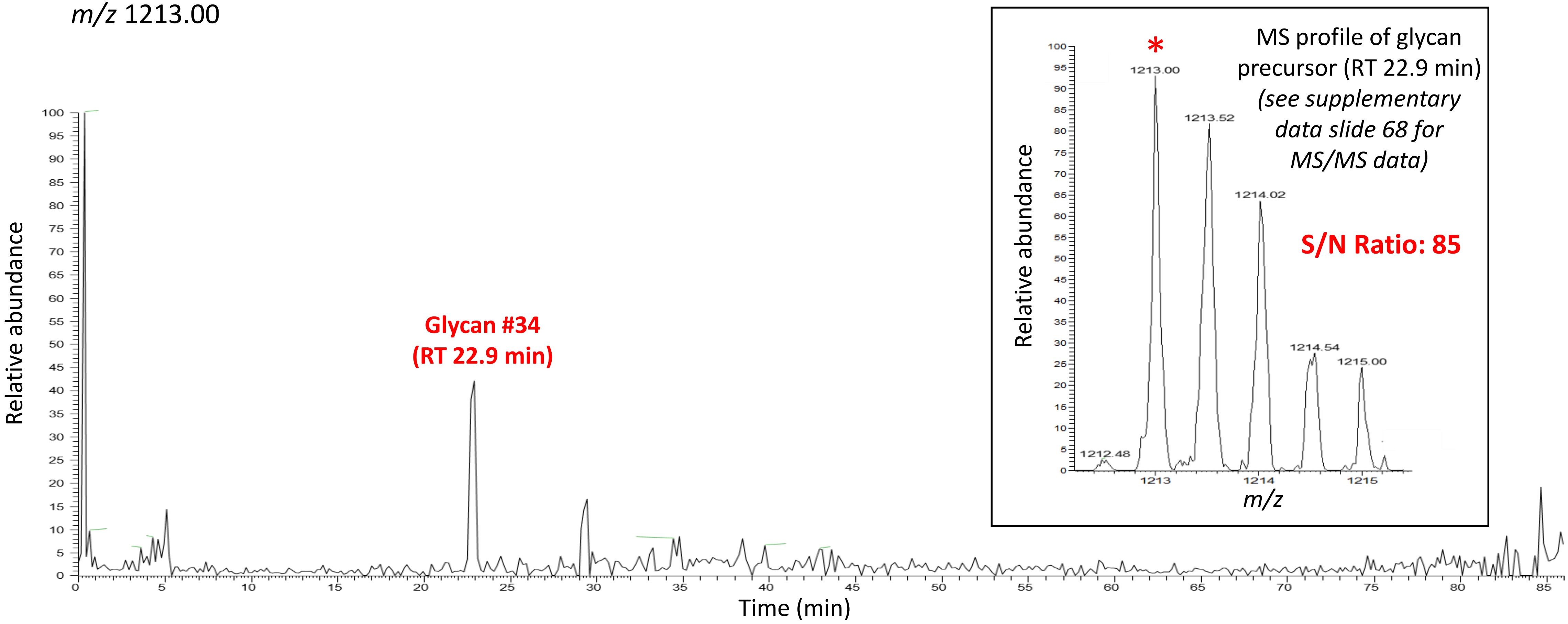

*Monoisotopic signal of identified glycan

Pseudomonas aeruginosa 13
Extracted Ion Chromatogram
m/z 1294.02

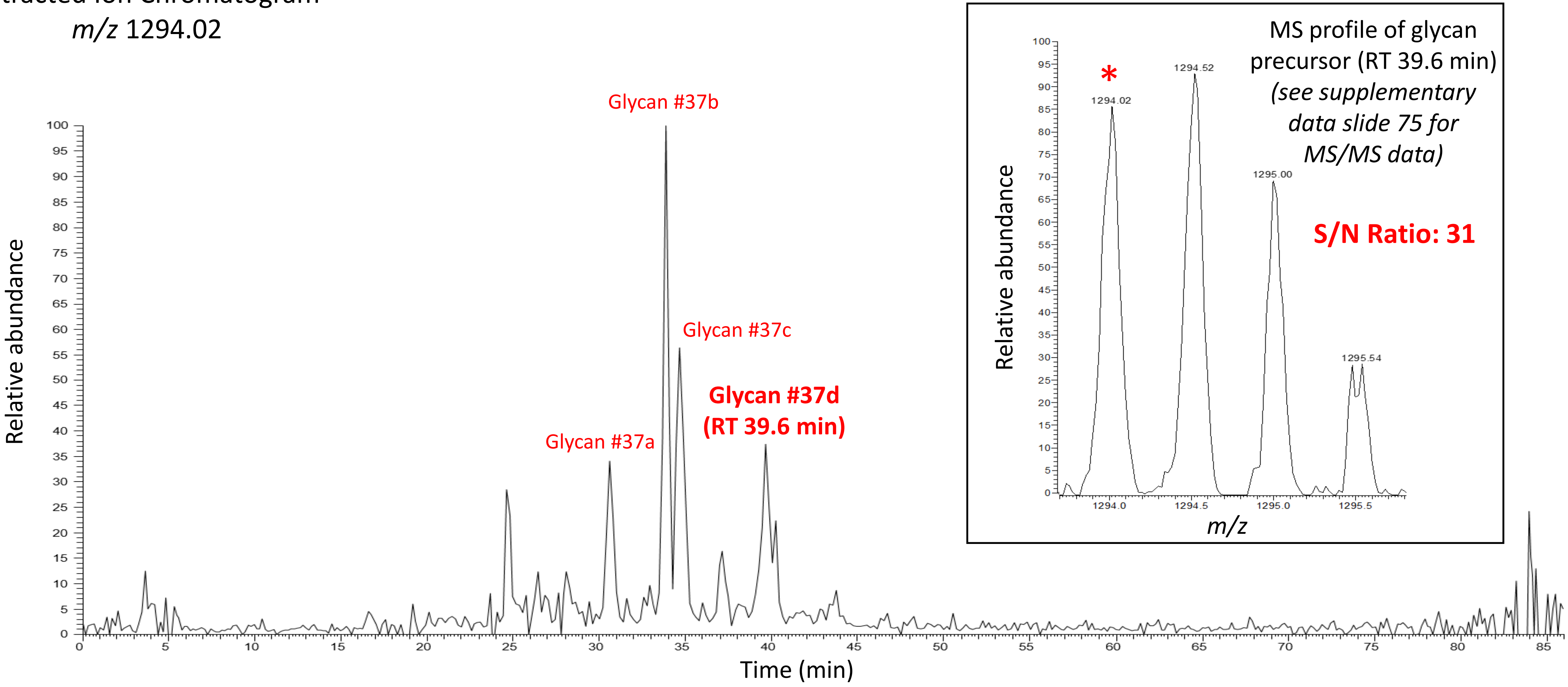

*Monoisotopic signal of identified glycan

Staphylococcus aureus 17
Extracted Ion Chromatogram
m/z 1512.60

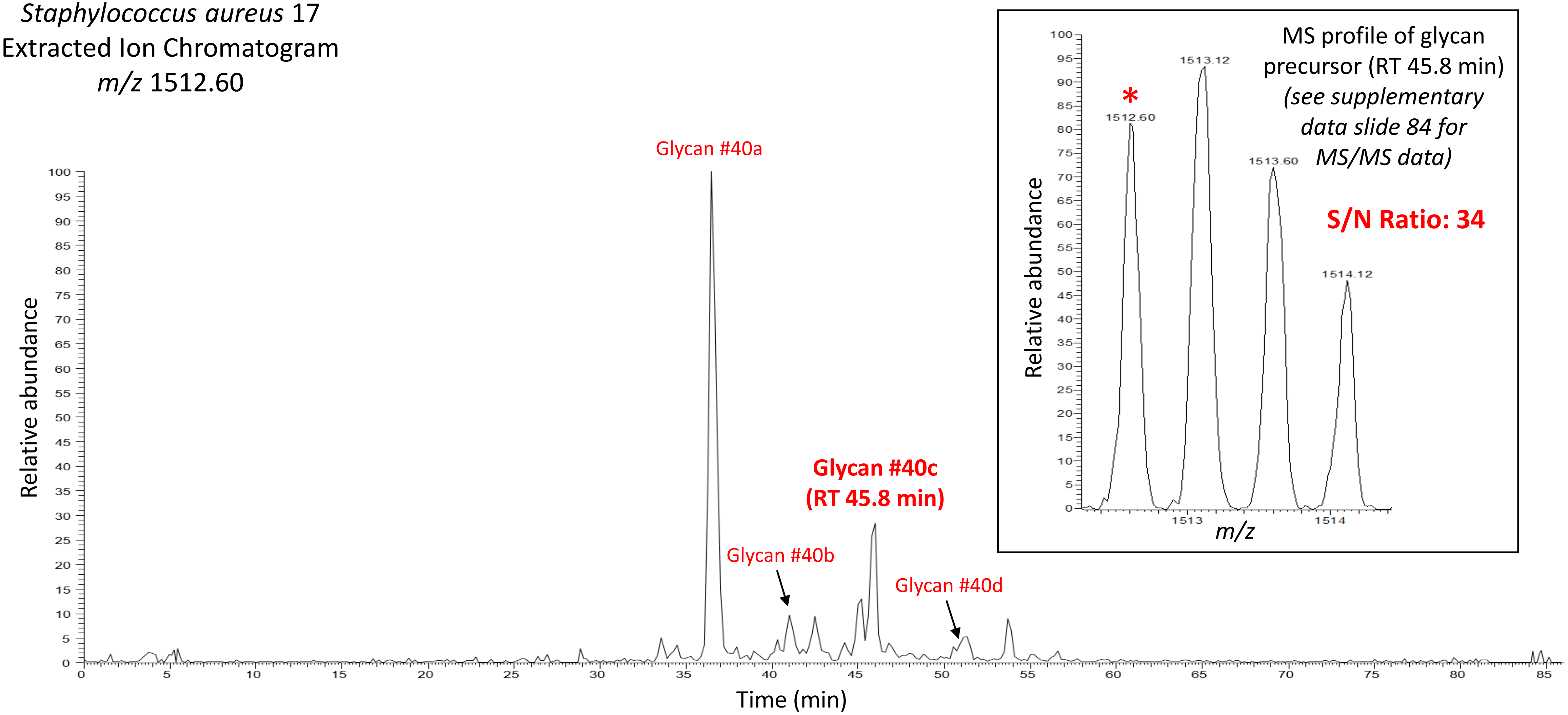

***Monoisotopic signal of identified glycan**

Supplementary Data S3

Manually annotated PGC-LC-ESI-CID-MS/MS (-) spectra of all reported *N*-glycans (alditols) released from bacteremic and healthy sera

Glycan #1

Observed *m/z* 1235.48 (1-), RT: ~23.4 min

[M-H]$^-$ 1235.48 Da

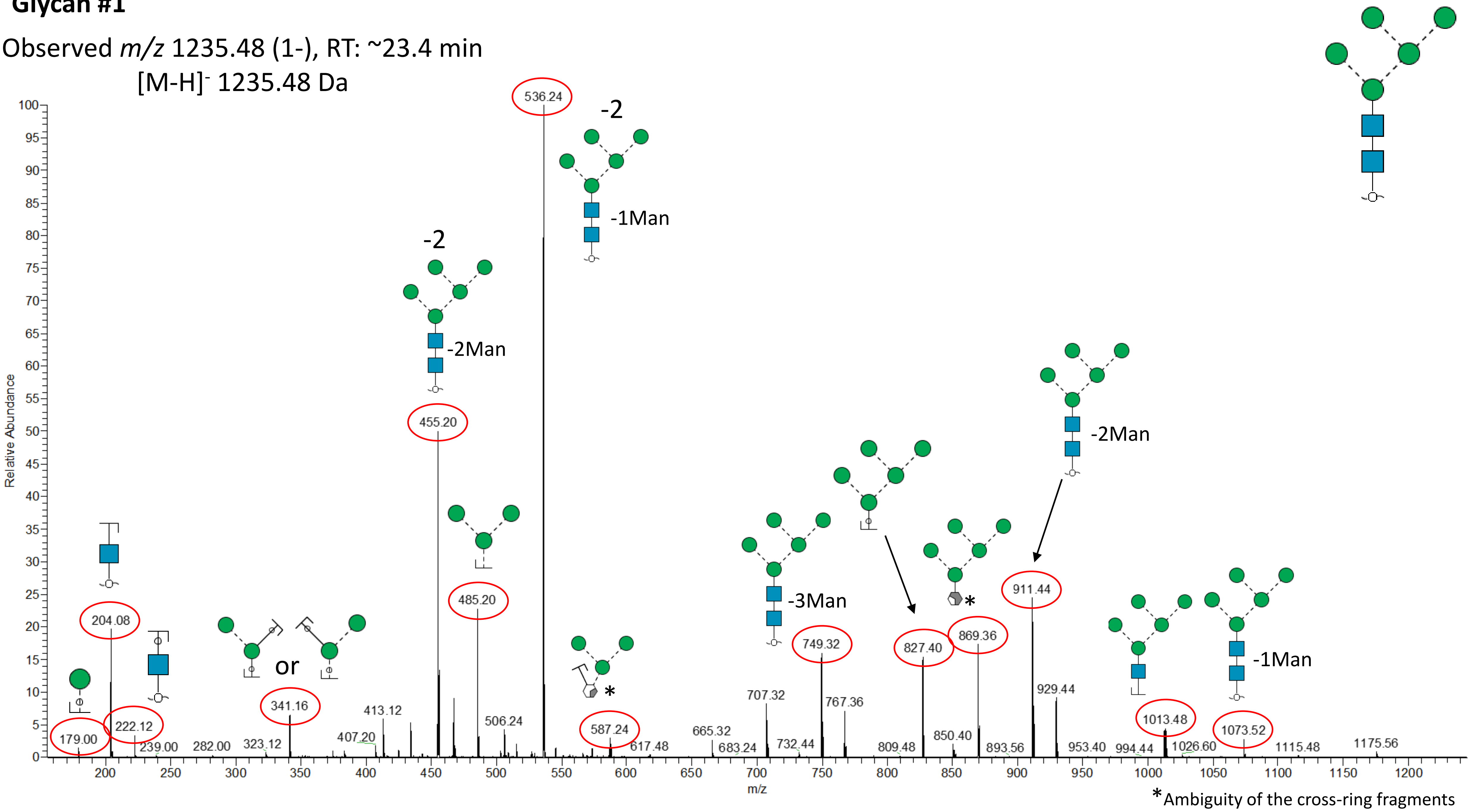

Glycan #2

Observed *m/z* 698.26 (2-), RT: ~19.3 min
[M-H]- 1397.49 Da

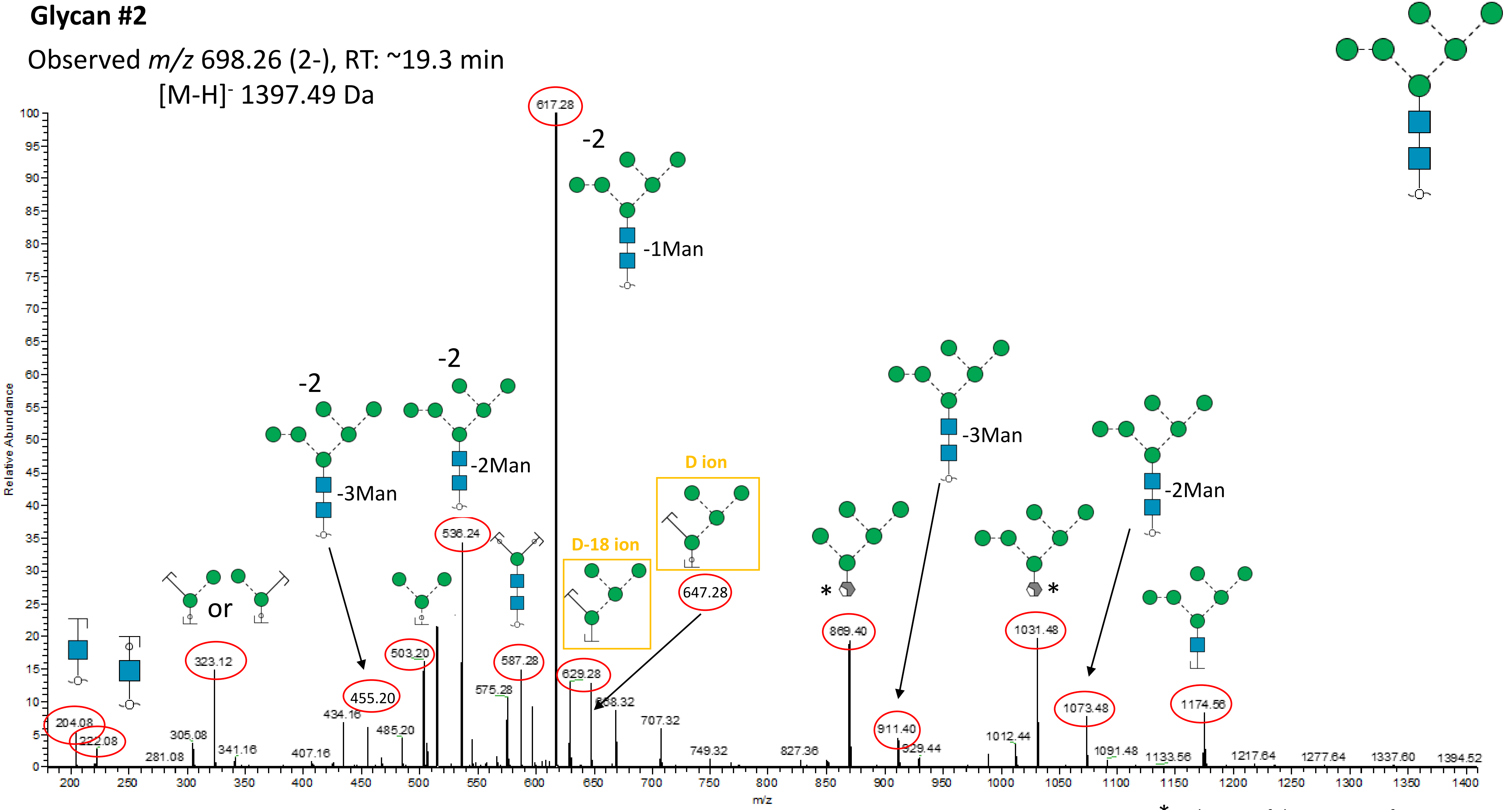

*Ambiguity of the cross-ring fragments

Glycan #3

Observed *m/z* 779.30 (2-), RT: ~18.9 min

$[M-H]^-$ 1559.54 Da

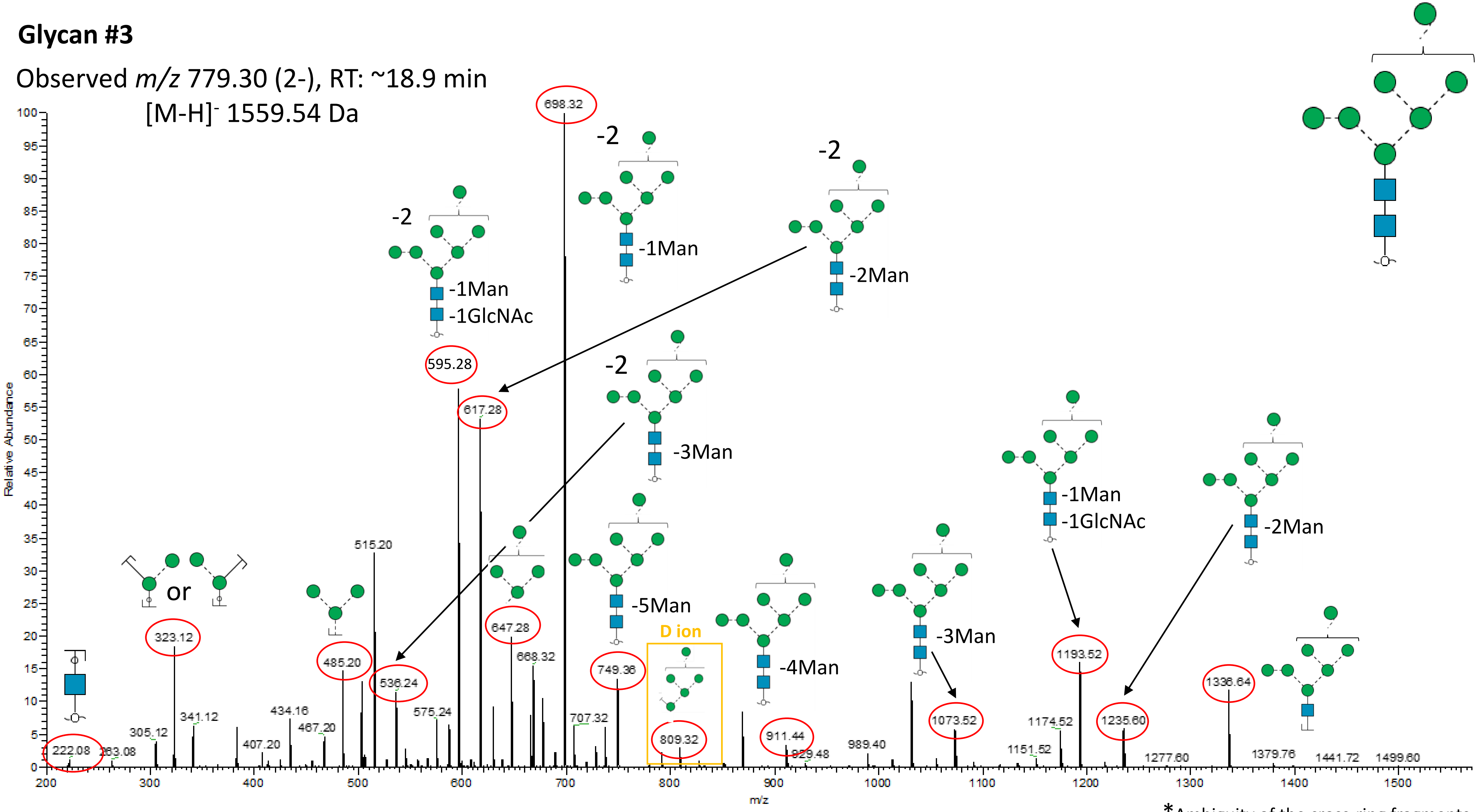

*Ambiguity of the cross-ring fragments

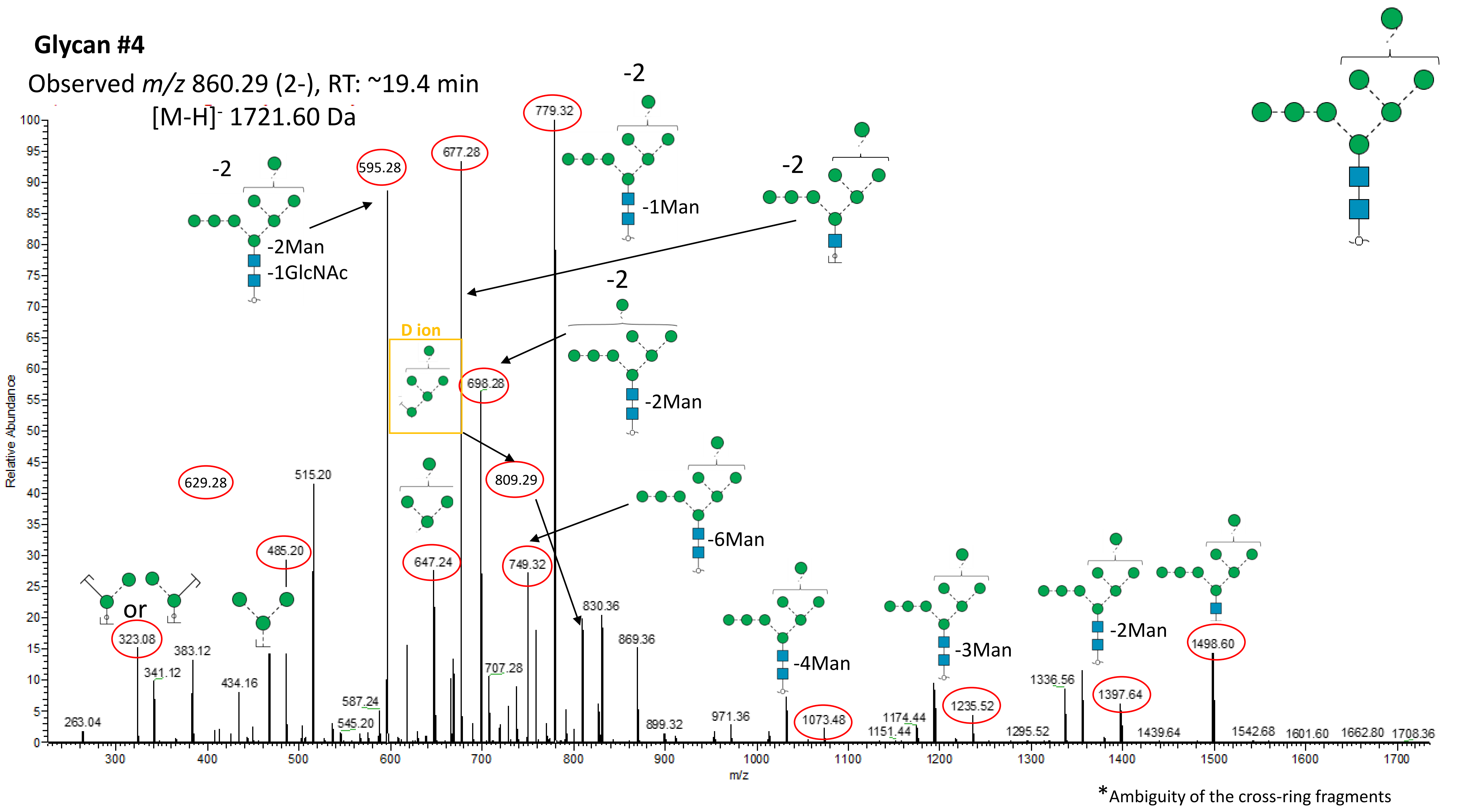

Glycan #4
Observed m/z 860.29 (2-), RT: ~19.4 min
[M-H]- 1721.60 Da
-2
-2Man
-1GlcNAc
-1Man
-2Man
-6Man
-4Man
-3Man
D ion
or
Relative Abundance
m/z
263.04
323.08
341.12
383.12
434.16
485.20
515.20
545.20
587.24
595.28
629.28
647.24
677.28
698.28
707.28
749.32
779.32
809.29
830.36
869.36
899.32
971.36
1073.48
1151.44
1174.44
1235.52
1295.52
1336.56
1397.64
1439.64
1498.60
1542.68
1601.60
1662.80
1708.36
*Ambiguity of the cross-ring fragments

Glycan #5

Observed *m/z* 941.36 (2-), RT: ~19.2 min

[M-H]- 1883.65 Da

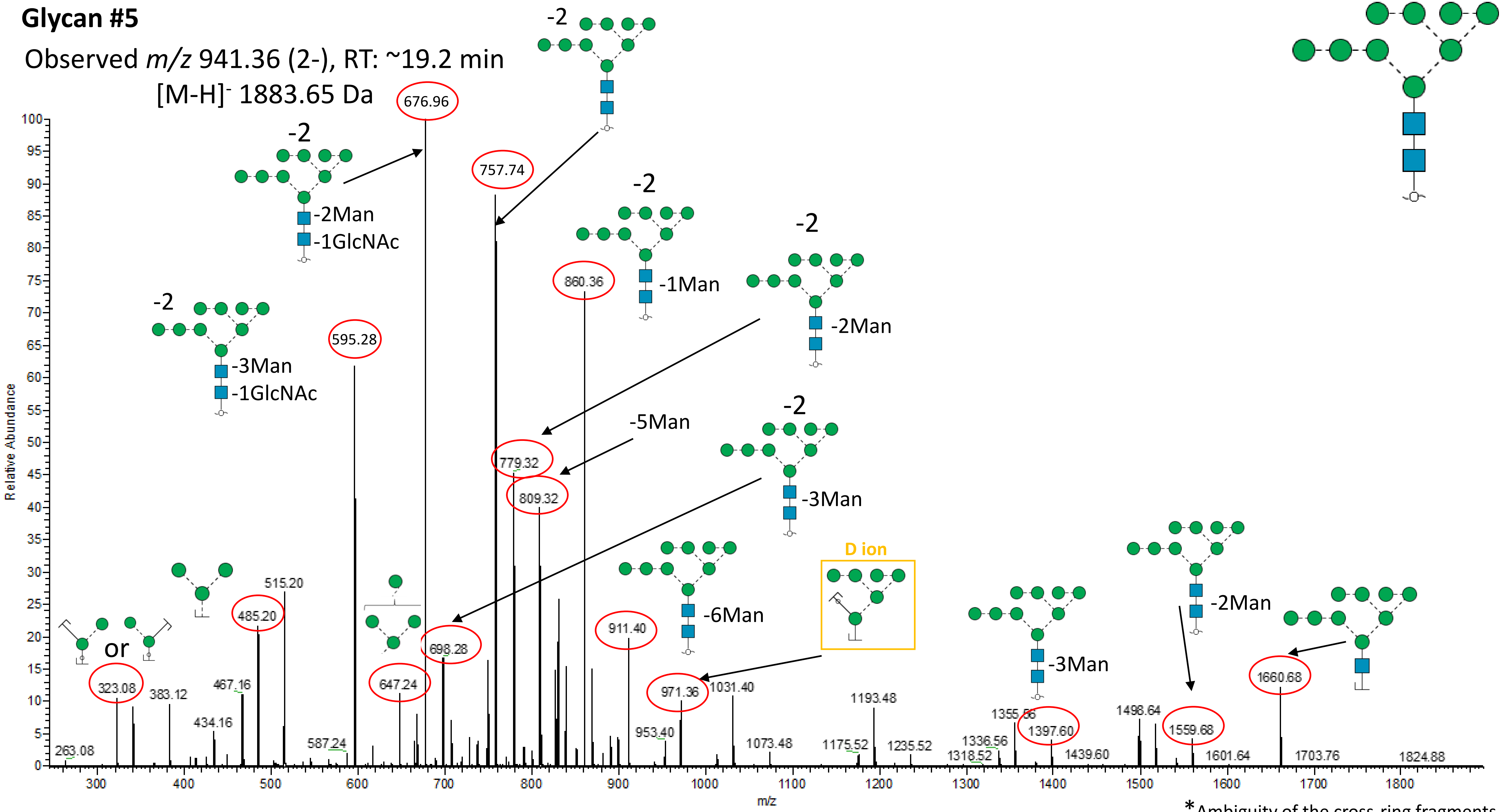

*Ambiguity of the cross-ring fragments

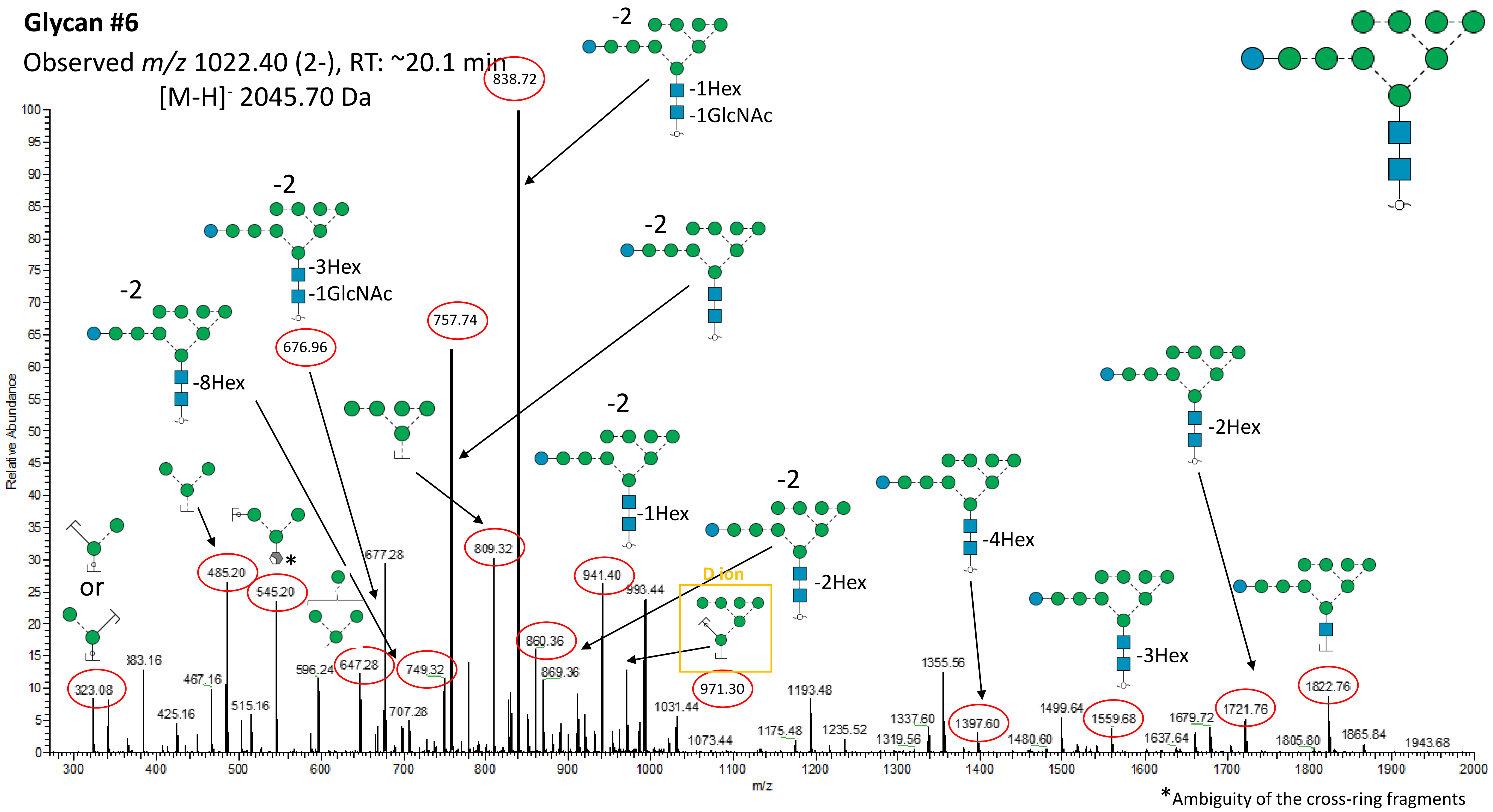

Glycan #6
Observed m/z 1022.40 (2-), RT: ~20.1 min
[M-H]- 2045.70 Da
-2
-1Hex
-1GlcNAc
838.72
-3Hex
-1GlcNAc
-8Hex
757.74
676.96
-1Hex
809.32
941.40
993.44
860.36
869.36
-2Hex
-4Hex
-2Hex
-3Hex
D ion
971.30
or
*
485.20
545.20
677.28
323.08
383.16
467.16
425.16
515.16
596.24
647.28
749.32
707.28
1031.44
1073.44
1175.48
1193.48
1235.52
1319.56
1337.60
1355.56
1397.60
1480.60
1499.64
1559.68
1637.64
1679.72
1721.76
1805.80
1822.76
1865.84
1943.68
Relative Abundance
m/z
*Ambiguity of the cross-ring fragments

Glycan #7

Observed *m/z* 718.78 (2-), RT: ~20.6 min

[M-H]- 1438.52 Da

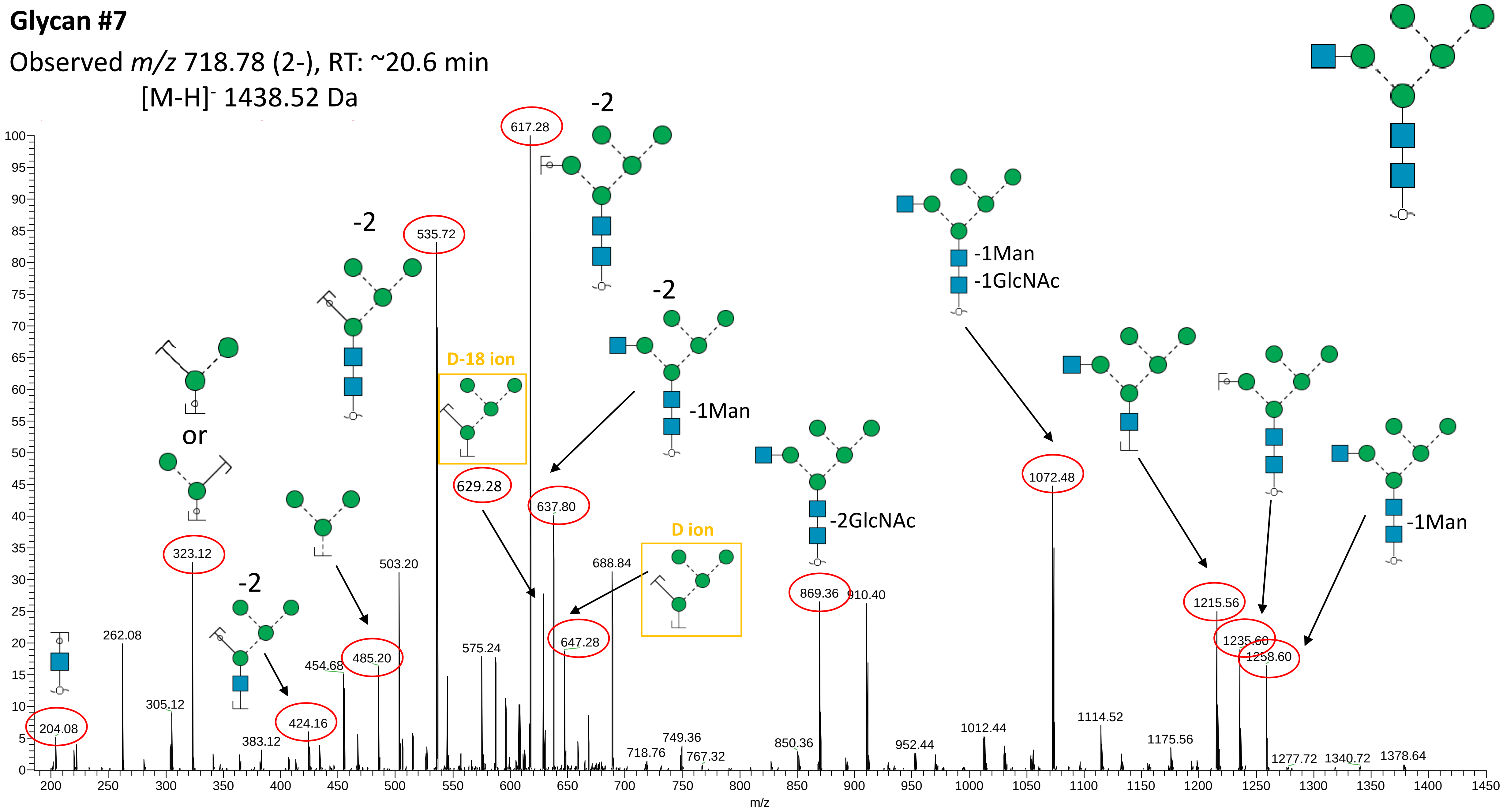

*Ambiguity in the cross-ring fragments

Glycan #8

Observed *m/z* 864.32 (2-), RT: ~22.5 min
[M-H]- 1729.61 Da

Note: Based on PGC-LC elution pattern, this glycan is annotated as the α2,6-sialyl linkage isomer.

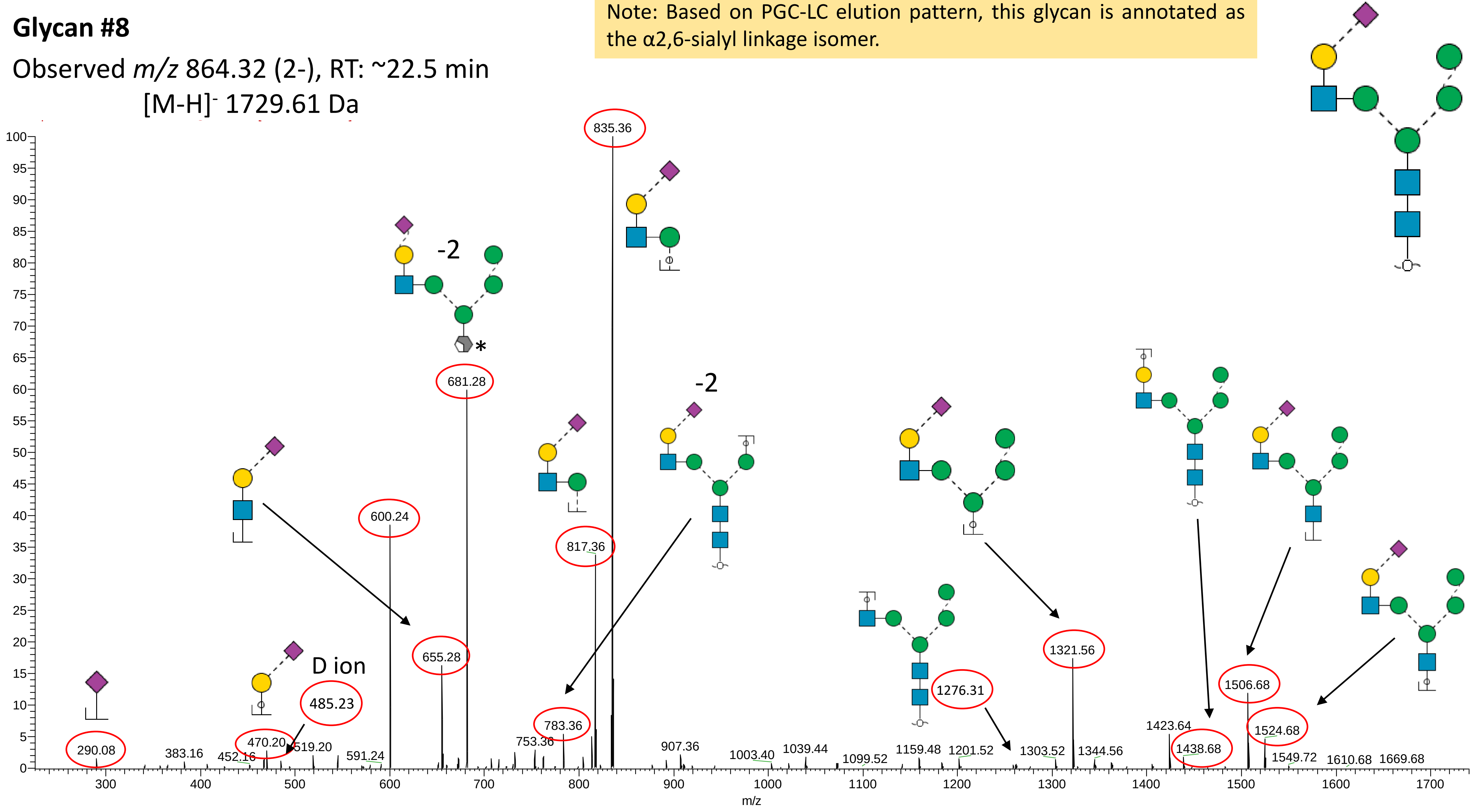

*Ambiguity in the cross-ring fragments

Glycan #9

Observed *m/z* 799.78 (2-), RT: ~23.2 min

$[M-H]^-$ 1600.57 Da

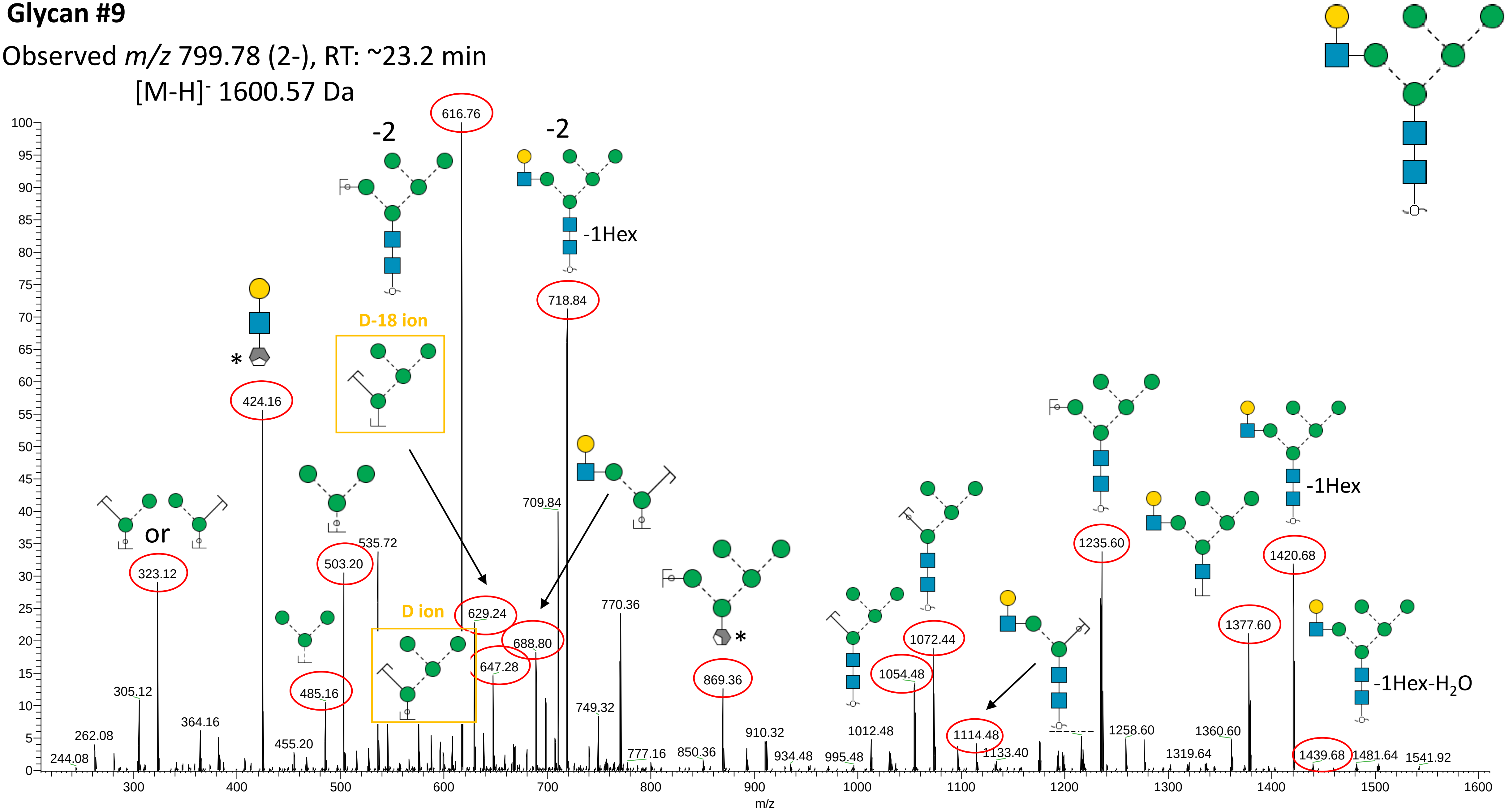

*Ambiguity in the cross-ring fragments

Glycan #10

Observed *m/z* 945.36 (2-), RT: ~25.1 min

[M-H]- 1891.66 Da

Note: Based on PGC-LC elution pattern, this glycan is annotated as the α2,6-sialyl linkage isomer.

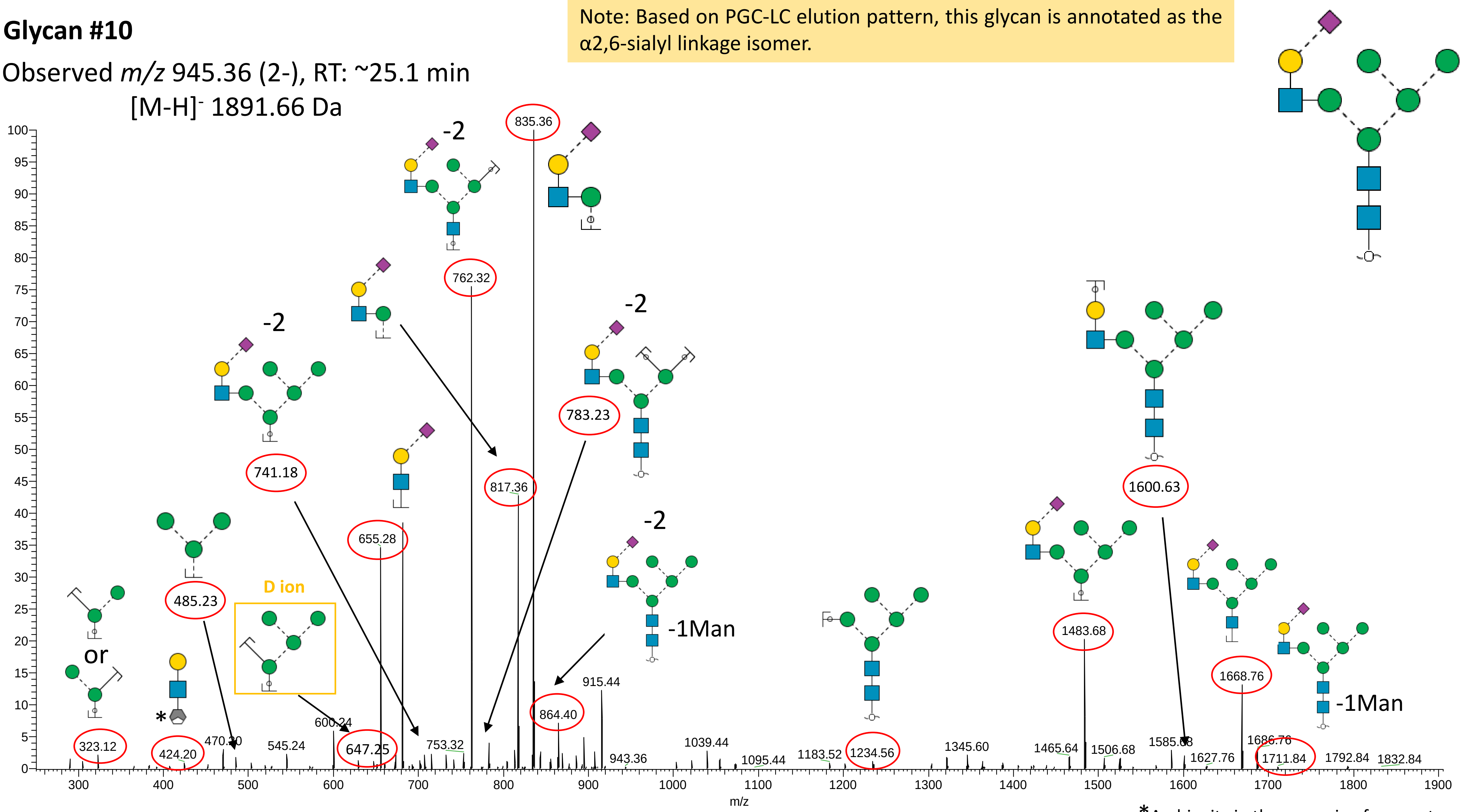

*Ambiguity in the cross-ring fragments

Glycan #11

Observed *m/z* 1260.48 (1-), RT: ~22.5 min

$[M-H]^-$ 1260.47 Da

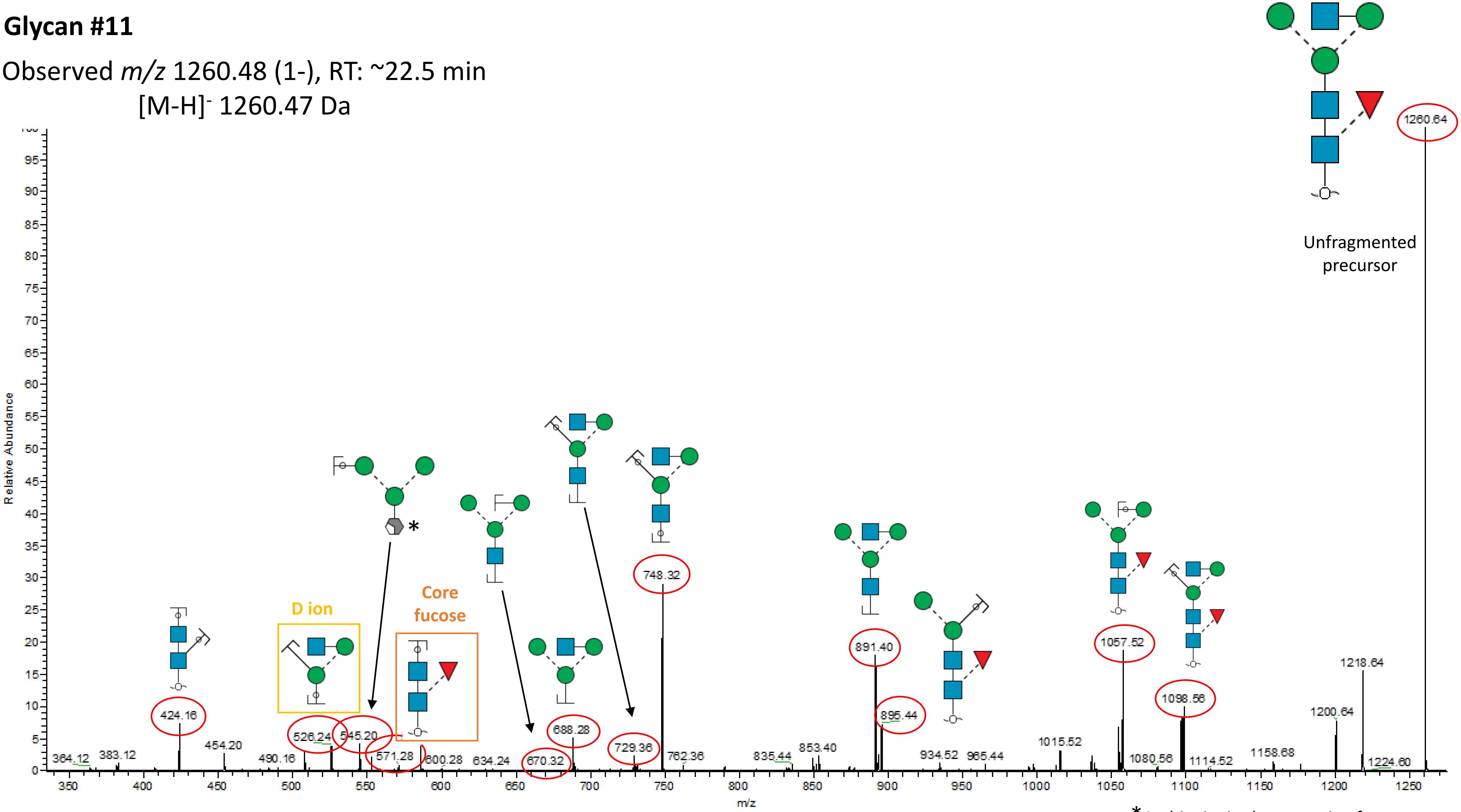

*Ambiguity in the cross-ring fragments

Glycan #12a

Observed *m/z* 783.30 (2-), RT: ~23.4 min

[M-H]⁻ 1567.56 Da

Note: From biosynthetic pathway and the lack of D and/or D-18 ions, monoantennary sialylation is predicted to occupy the α-1,3 arm. Based on early PGC-LC elution, this glycan is annotated as the α2,6-sialyl linkage isomer.

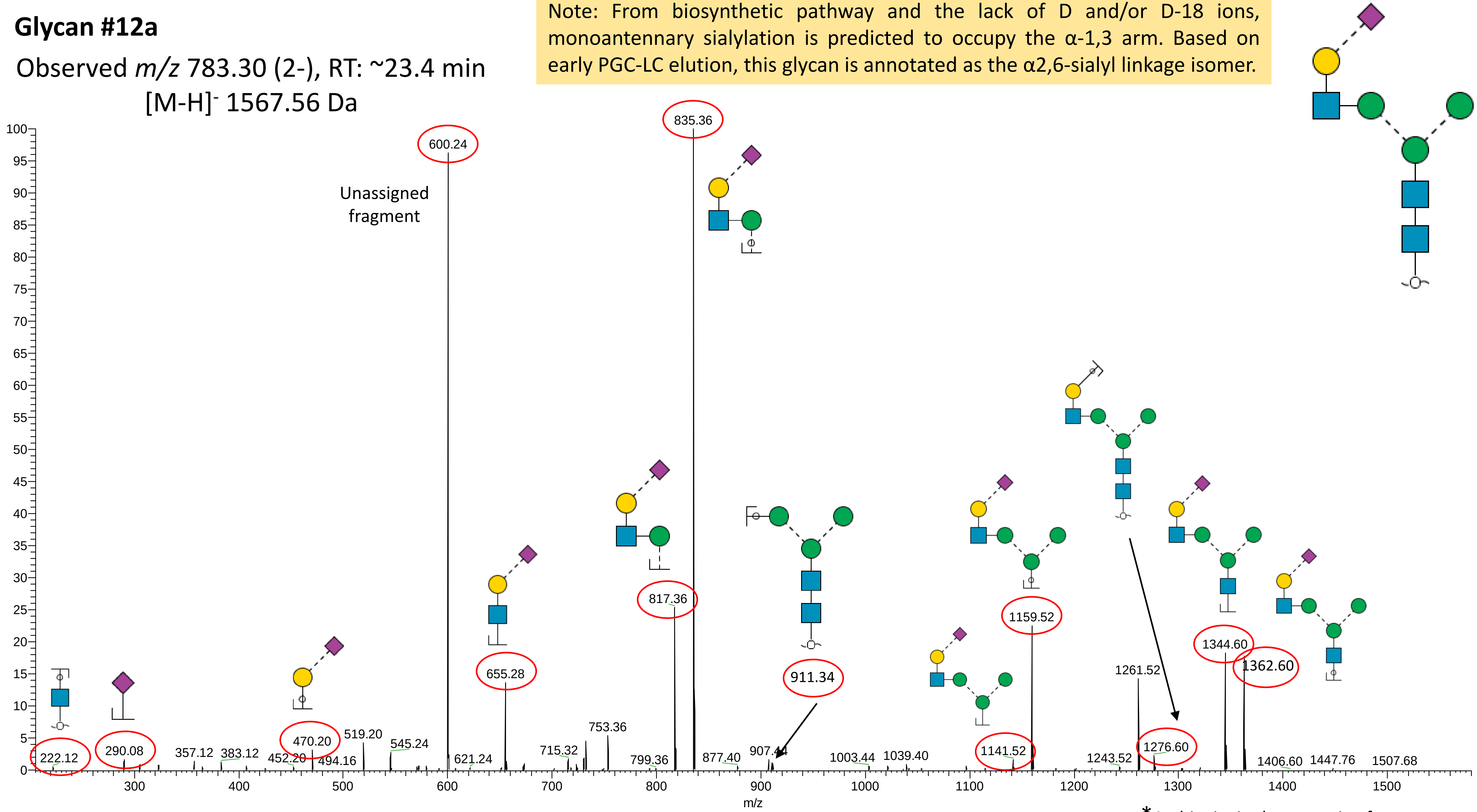

*Ambiguity in the cross-ring fragments

Glycan #12b

Observed *m/z* 783.30 (2-), RT: ~28.3 min

$[M-H]^-$ 1567.56 Da

Note: From biosynthetic pathway and the lack of D and/or D-18 ions, monoantennary sialylation is predicted to occupy the α-1,3 arm. Based on late PGC-LC elution, this glycan is annotated as the α2,3-sialyl linkage isomer.

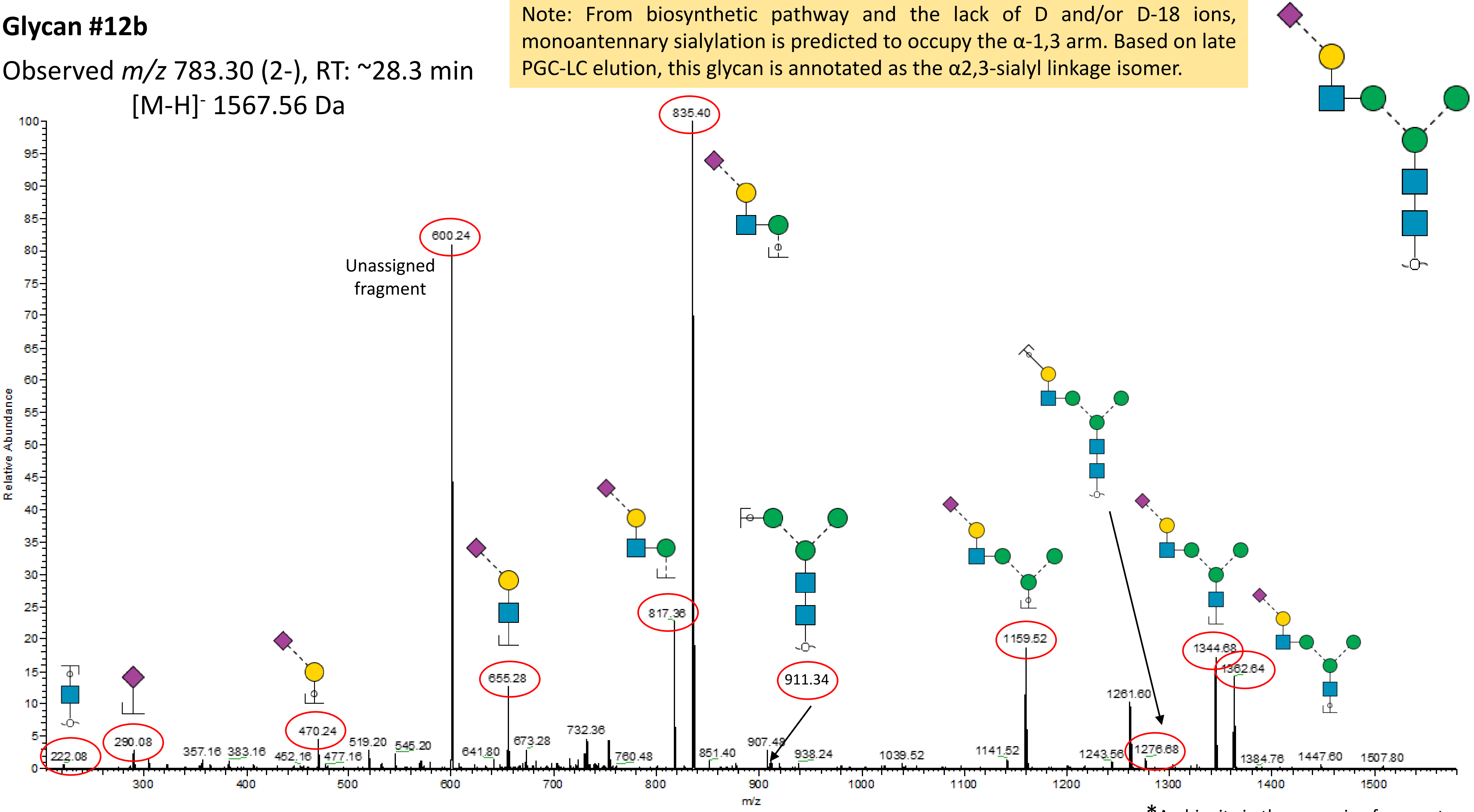

*Ambiguity in the cross-ring fragments

Glycan #13

Observed *m/z* 658.26 (2-), RT: ~20.5 min

$[M-H]^-$ 1317.49 Da

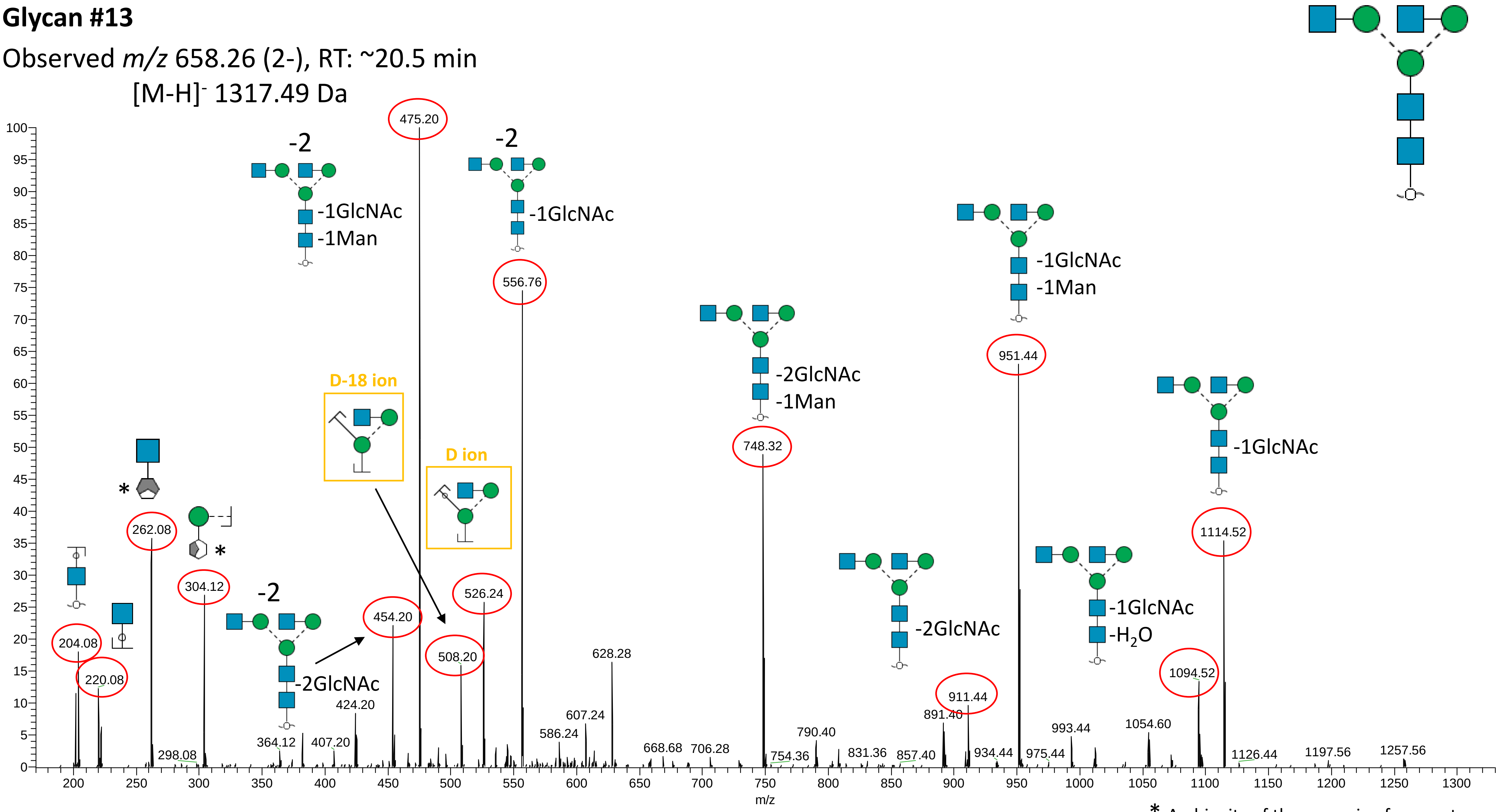

* Ambiguity of the cross-ring fragments

Glycan #14

Observed *m/z* 731.30 (2-), RT: ~24.7 min

$[M\text{-}H]^-$ 1463.55 Da

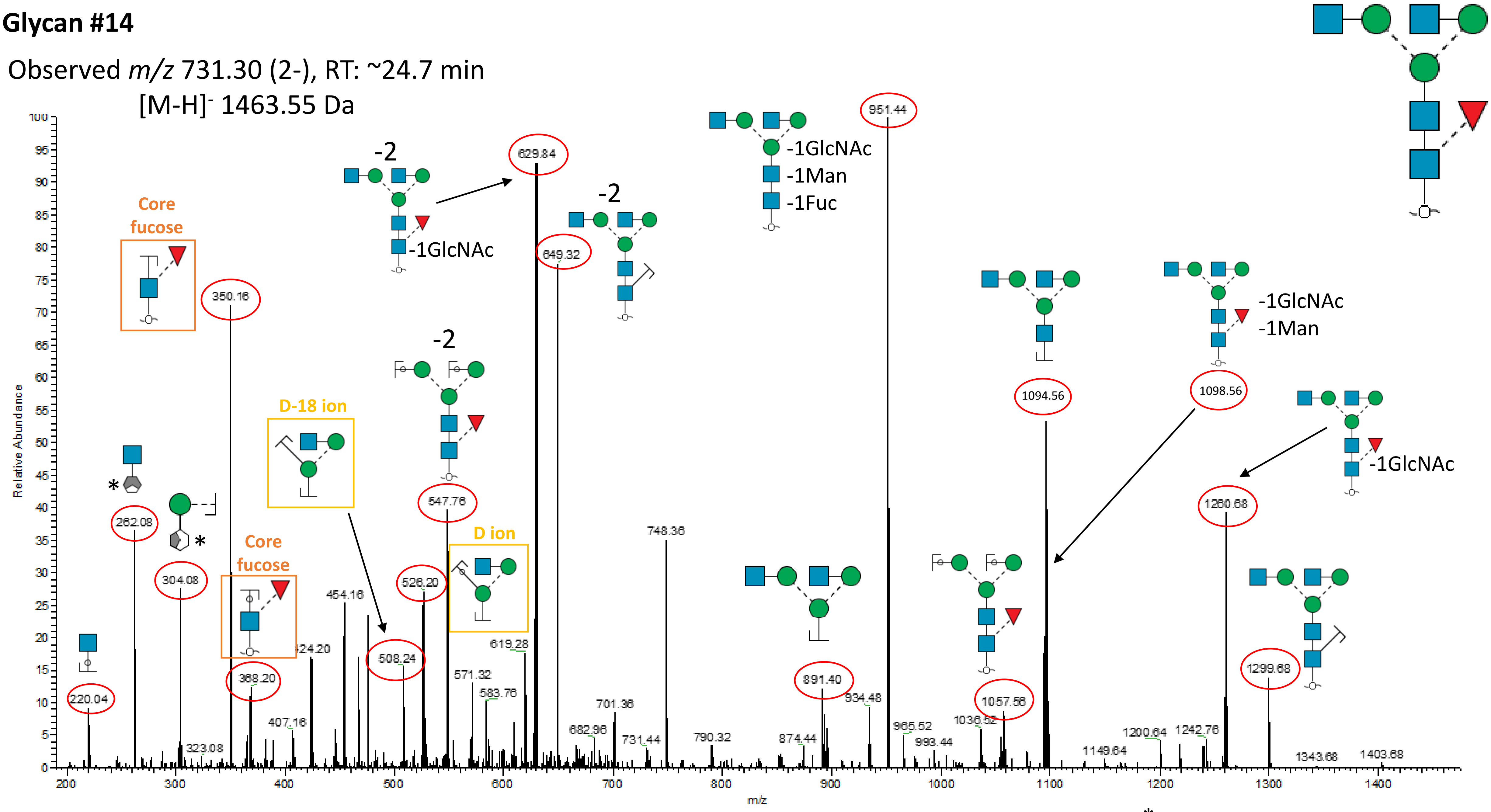

*Ambiguity of the cross-ring fragments

Glycan #15

Observed *m/z* 739.30 (2-), RT: ~22.3 min

[M-H]$^-$ 1479.54 Da

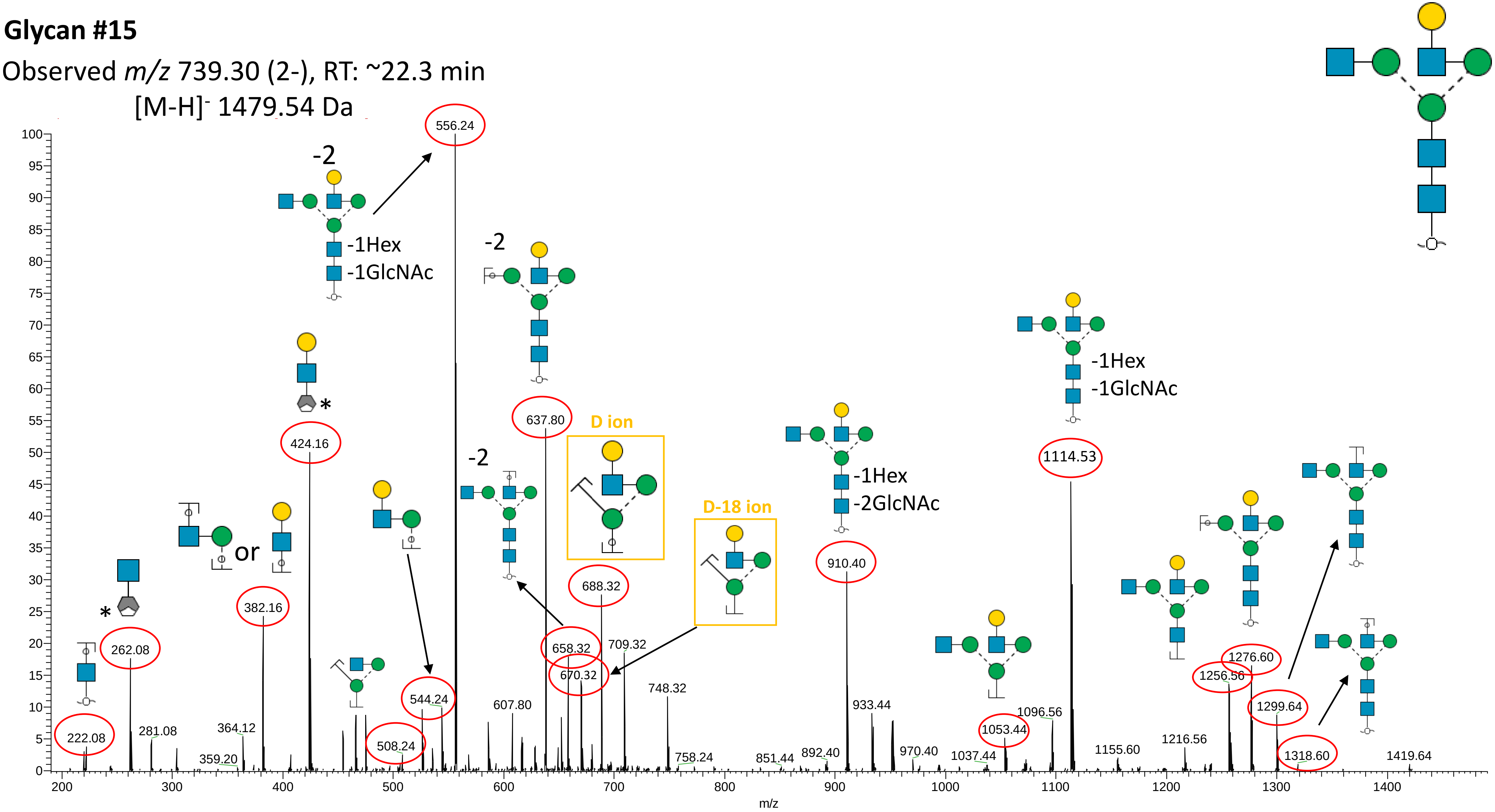

*Ambiguity of the cross-ring fragments

Glycan #16a

Observed *m/z* 812.32 (2-), RT: ~27.4 min

[M-H]- 1625.60 4 Da

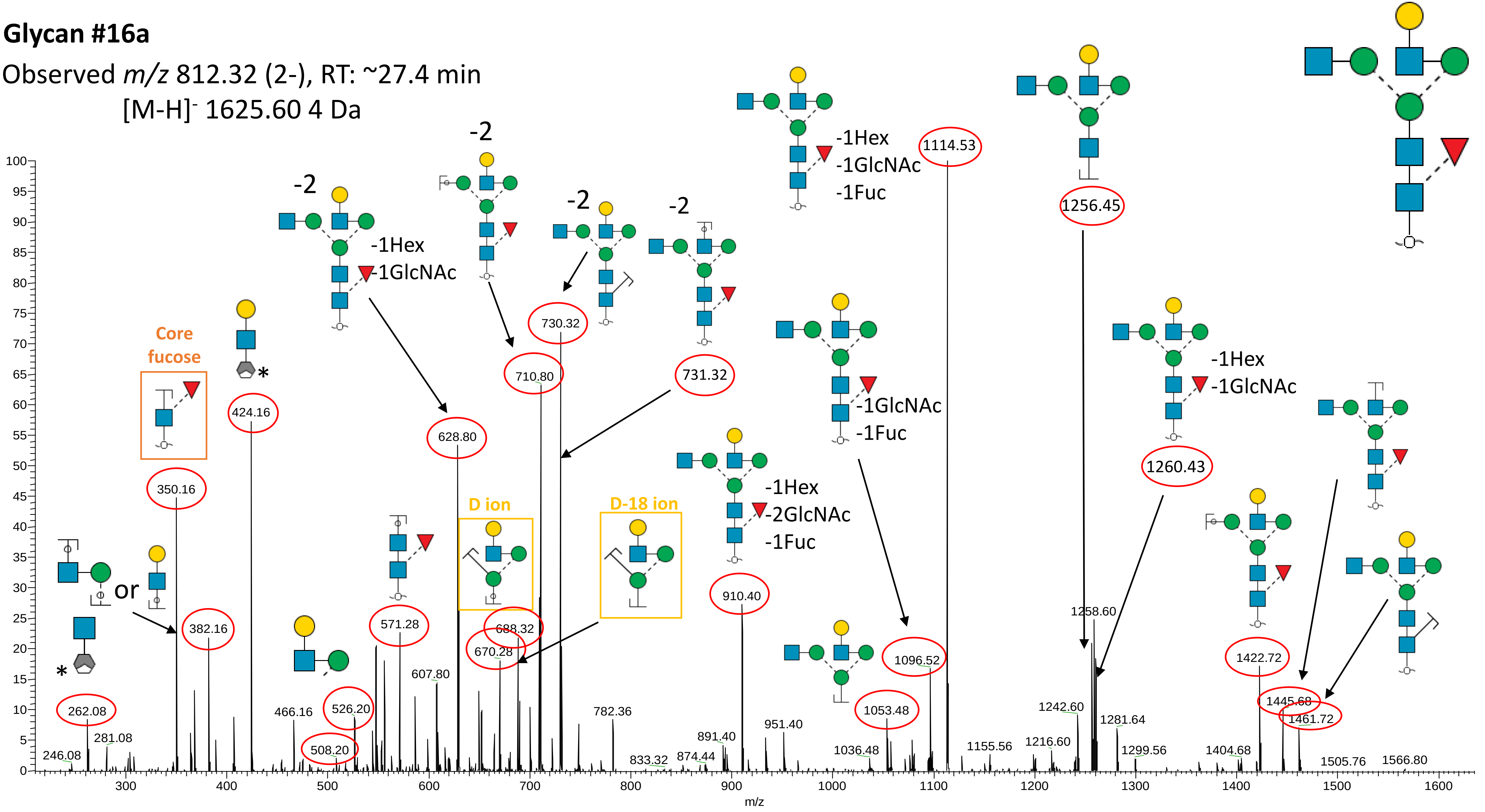

*Ambiguity of the cross-ring fragments

Glycan #16b

Observed *m/z* 812.32 (2-), RT: ~28.8 min

$[M\text{-}H]^-$ 1625.60 Da

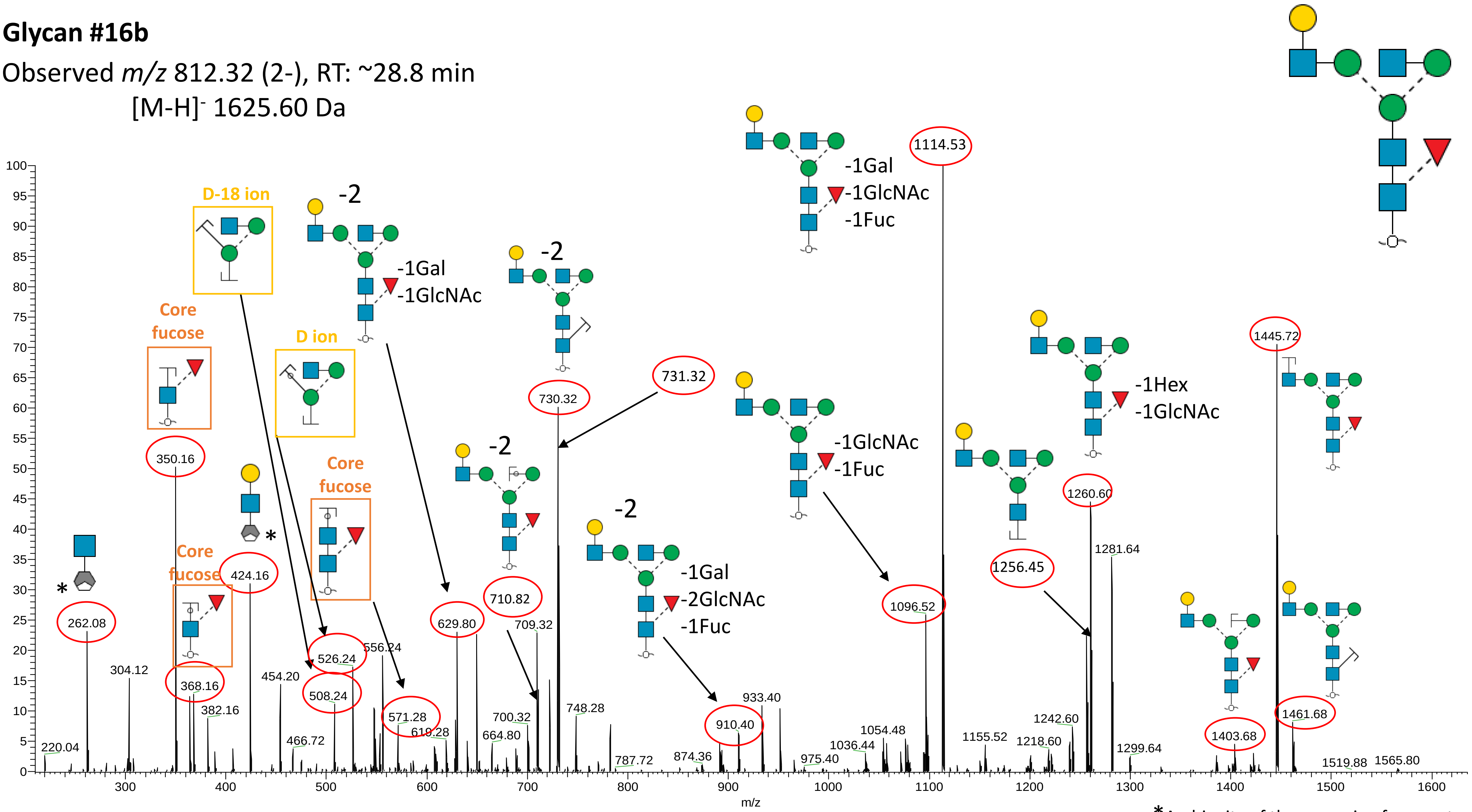

*Ambiguity of the cross-ring fragments

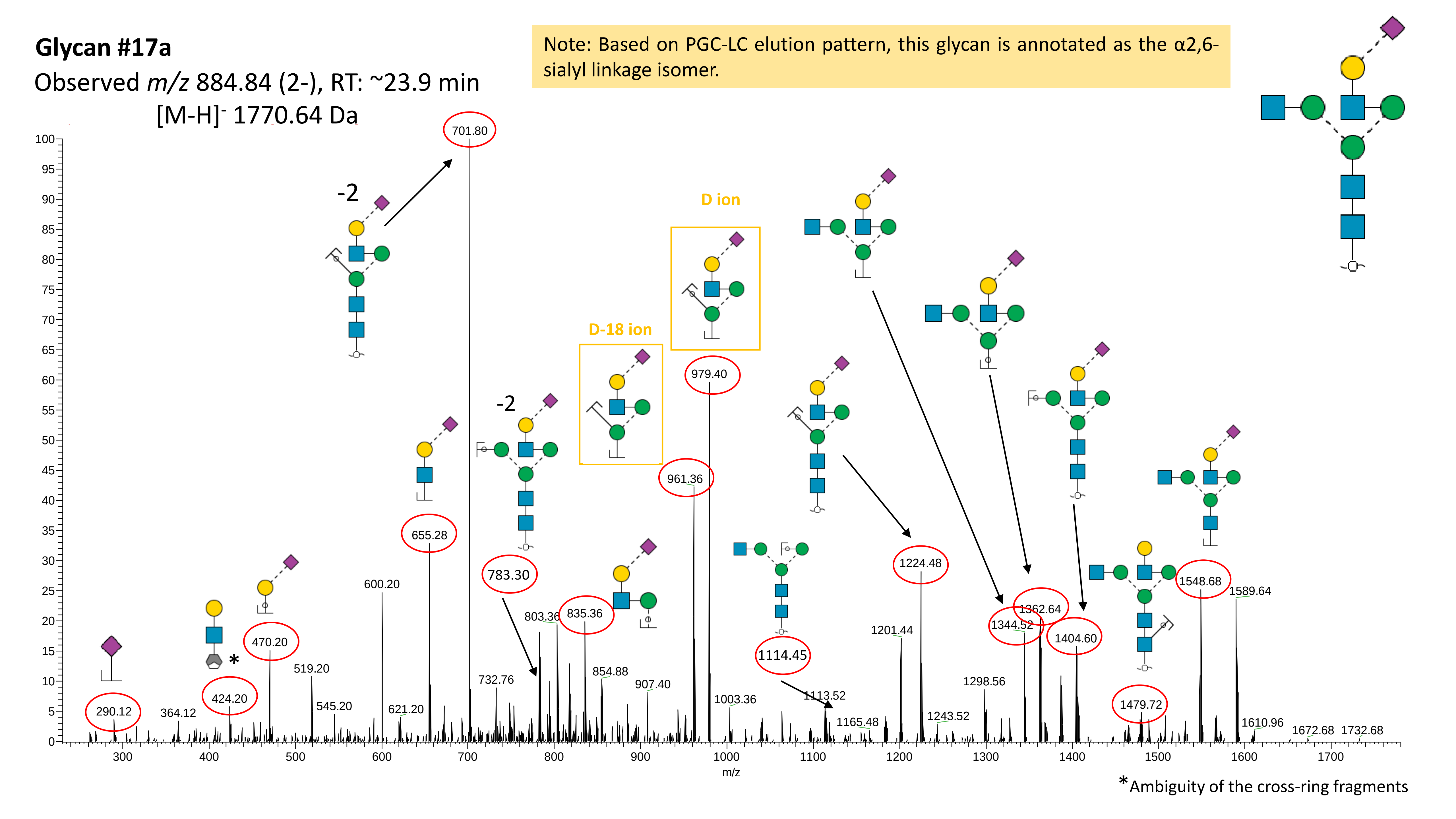
Glycan #17a
Observed m/z 884.84 (2-), RT: ~23.9 min
[M-H]- 1770.64 Da
Note: Based on PGC-LC elution pattern, this glycan is annotated as the α2,6-sialyl linkage isomer.
D ion
D-18 ion
-2
-2
*
701.80
979.40
961.36
655.28
783.30
835.36
803.36
600.20
470.20
424.20
290.12
364.12
519.20
545.20
621.20
732.76
854.88
907.40
1003.36
1113.52
1114.45
1165.48
1201.44
1224.48
1243.52
1298.56
1344.52
1362.64
1404.60
1479.72
1548.68
1589.64
1610.96
1672.68
1732.68
m/z
*Ambiguity of the cross-ring fragments

Glycan #17b

Observed *m/z* 884.84 (2-), RT: ~25.2 min

[M-H]- 1625.60 Da

Note: Based on PGC-LC elution pattern, this glycan is annotated as the α2,6-sialyl linkage isomer.

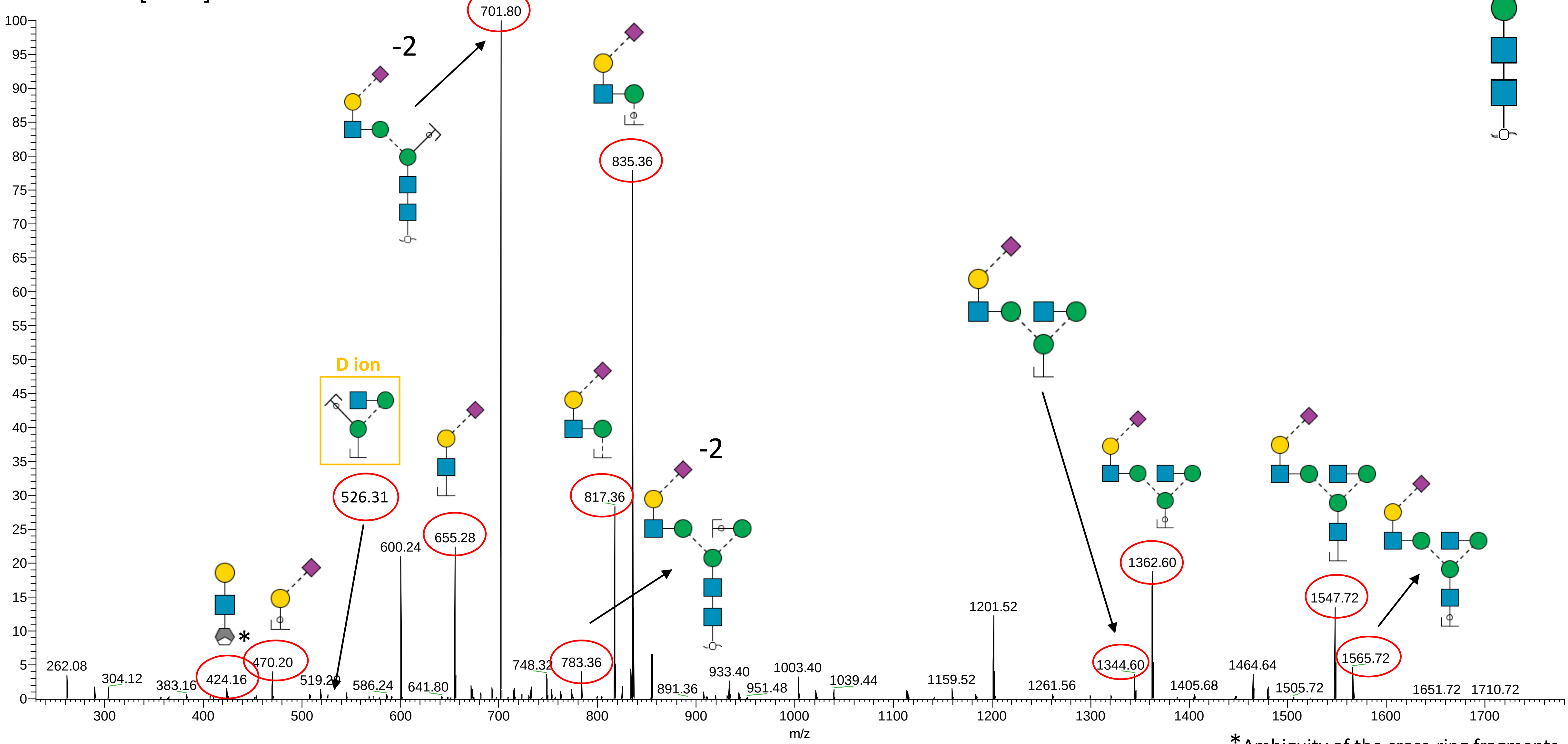

*Ambiguity of the cross-ring fragments

Glycan #18

Observed *m/z* 957.88 (2-), RT: ~29.3 min

$[M-H]^{-}$ 1916.70 Da

Note: Based on PGC-LC elution pattern, this glycan is annotated as the α2,6-sialyl linkage isomer.

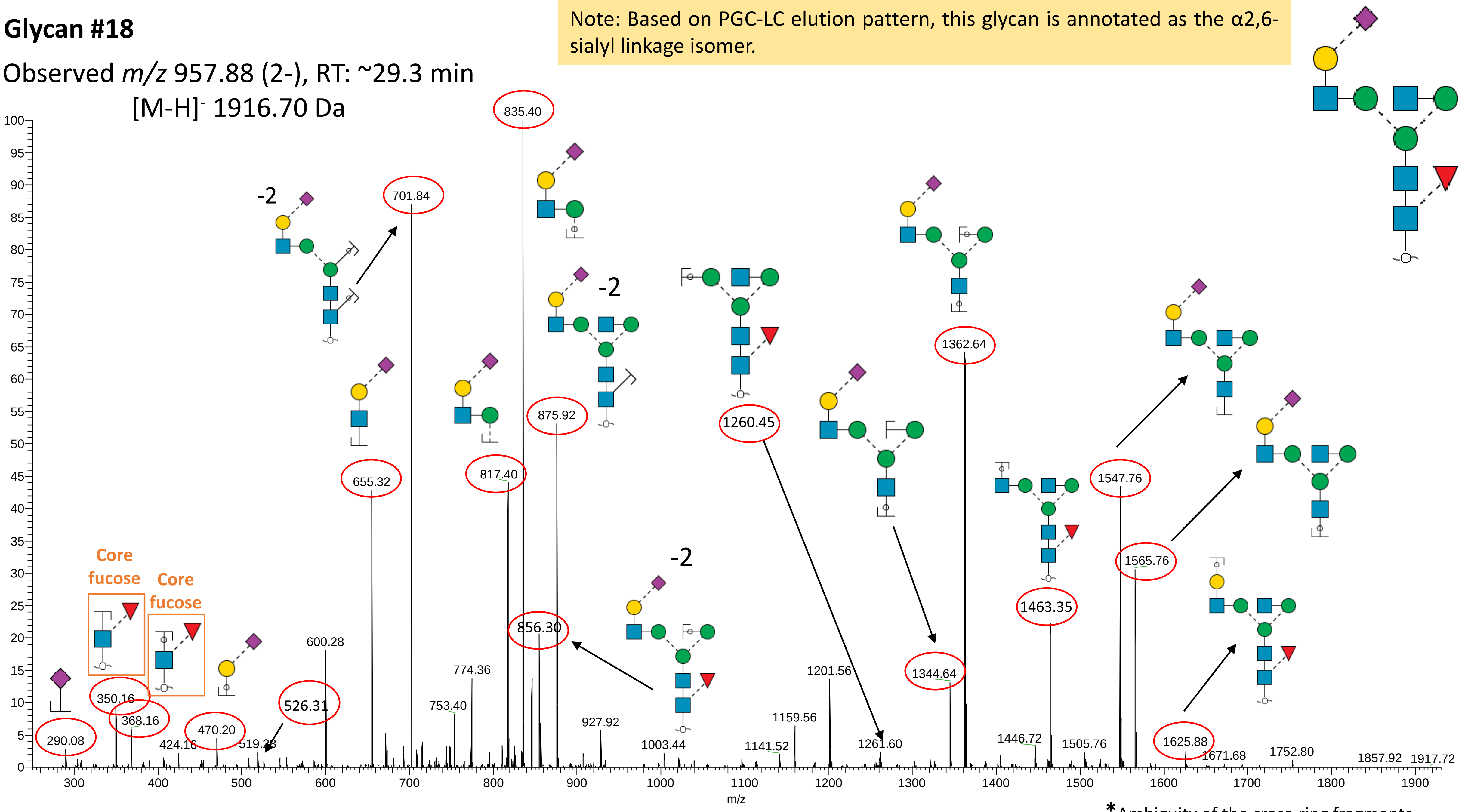

*Ambiguity of the cross-ring fragments

Glycan #19

Observed *m/z* 820.32 (2-), RT: ~25.6 min

$[M-H]^-$ 1641.60 Da

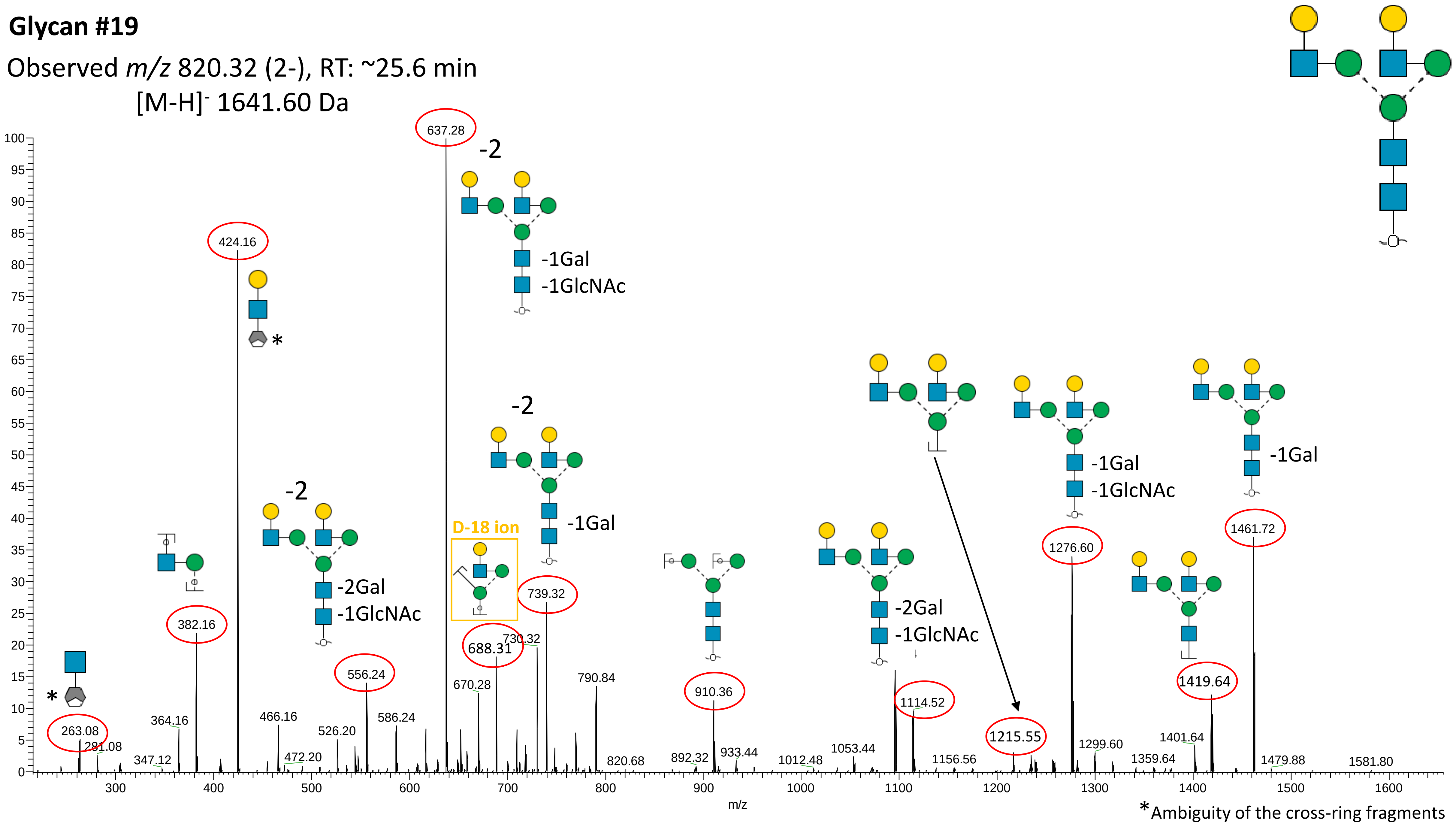

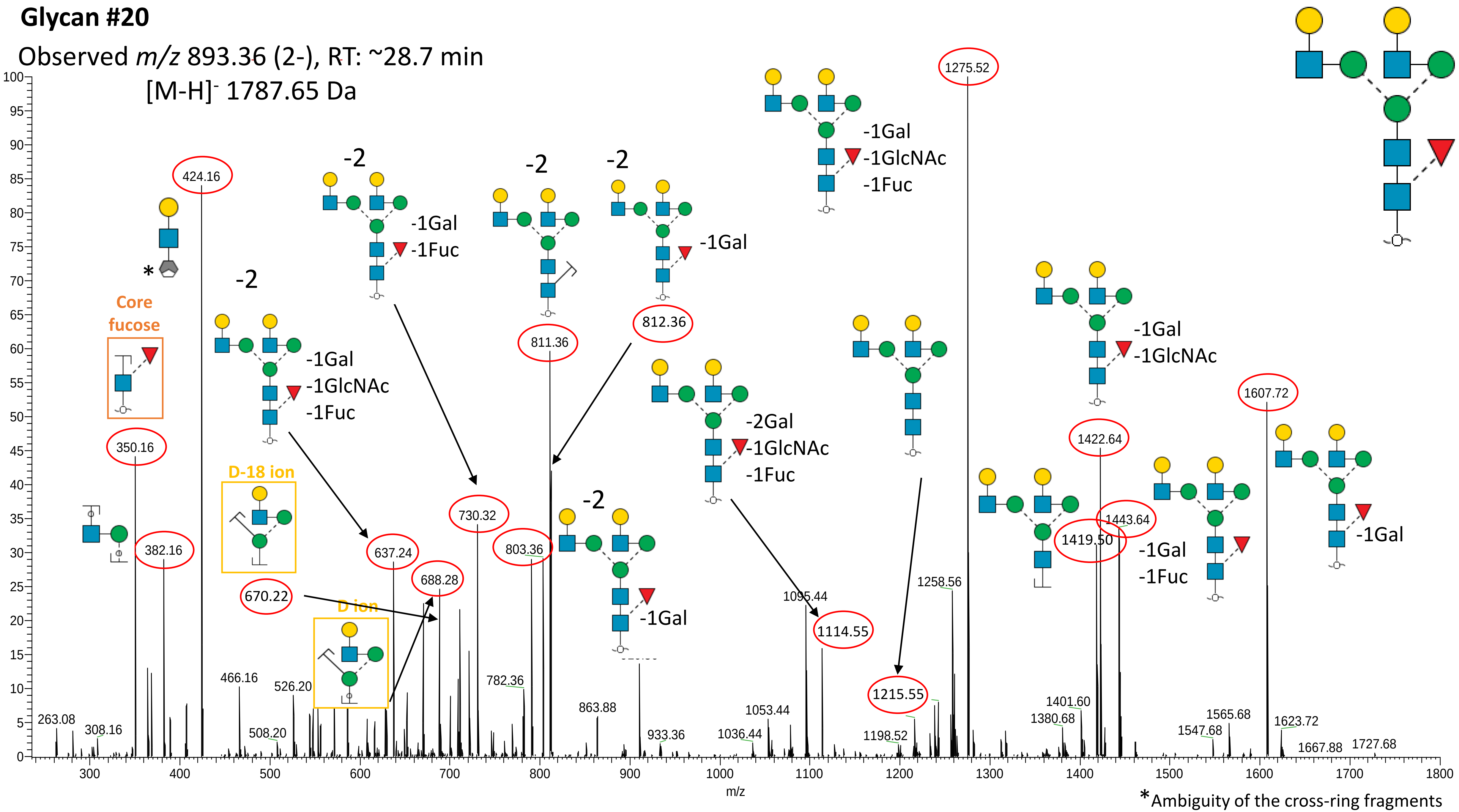
Glycan #20
Observed m/z 893.36 (2-), RT: ~28.7 min
[M-H]- 1787.65 Da
Core fucose
D-18 ion
D ion
-1Gal
-1Fuc
-1GlcNAc
-2Gal
-2
424.16
350.16
382.16
637.24
670.22
688.28
730.32
803.36
811.36
812.36
1114.55
1215.55
1275.52
1419.50
1422.64
1443.64
1607.72
263.08
308.16
466.16
508.20
526.20
782.36
863.88
933.36
1036.44
1053.44
1095.44
1198.52
1258.56
1380.68
1401.60
1547.68
1565.68
1623.72
1667.88
1727.68
m/z
*Ambiguity of the cross-ring fragments

Glycan #21

Observed *m/z* 965.88 (2-), RT: ~25.2 min

$[M-H]^-$ 1932.69 Da

Note: Based on PGC-LC elution pattern, this glycan is annotated as the α2,6-sialyl linkage isomer.

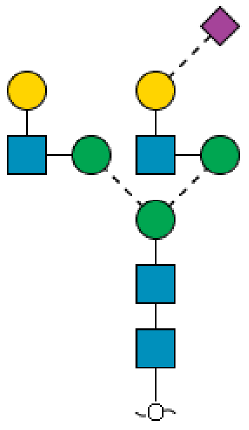

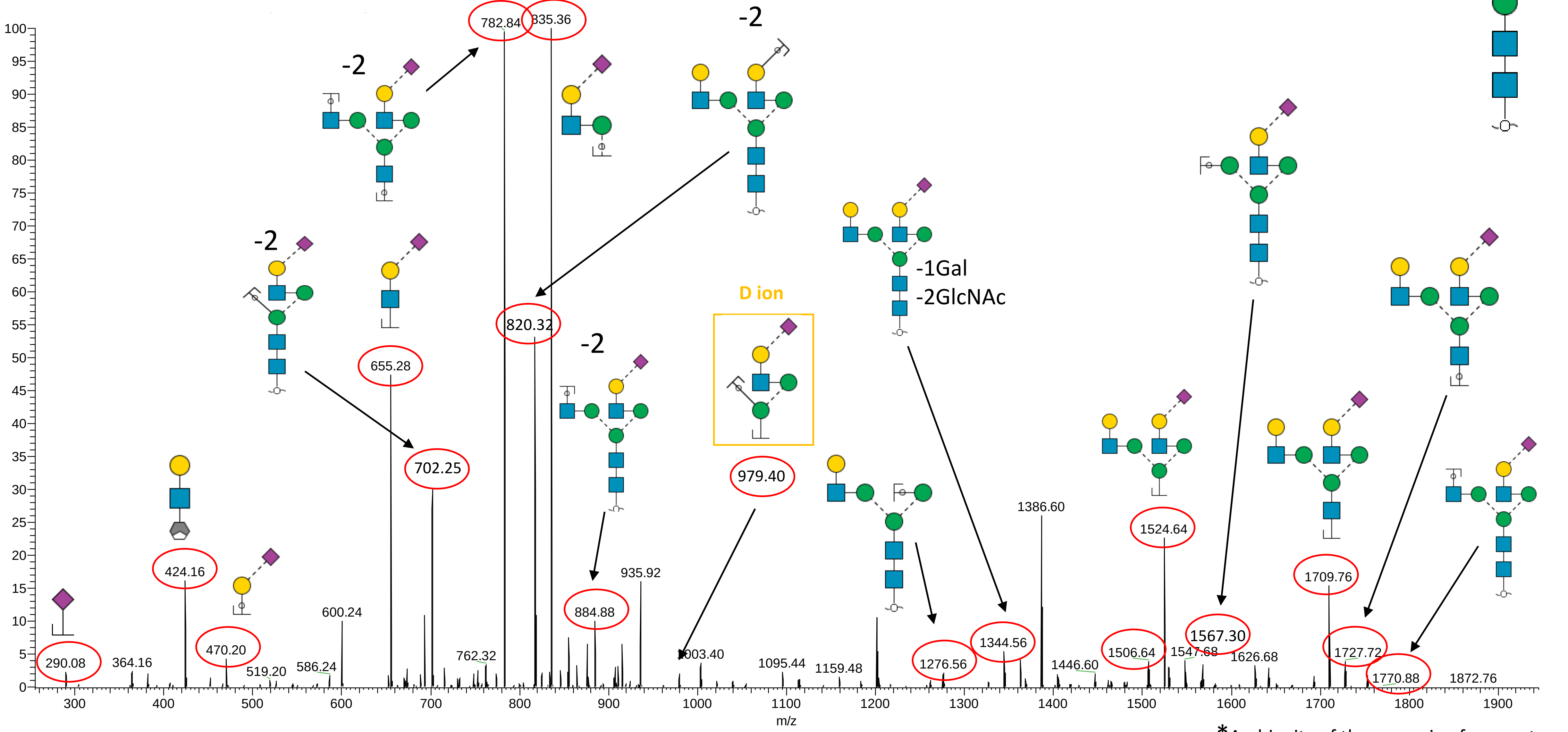

*Ambiguity of the cross-ring fragments

Glycan #22a

Observed *m/z* 1038.90 (2-), RT: ~30.7 min

$[M-H]^-$ 2078.75 Da

Note: Based on PGC-LC elution pattern, this glycan is annotated as the α2,6-sialyl linkage isomer.

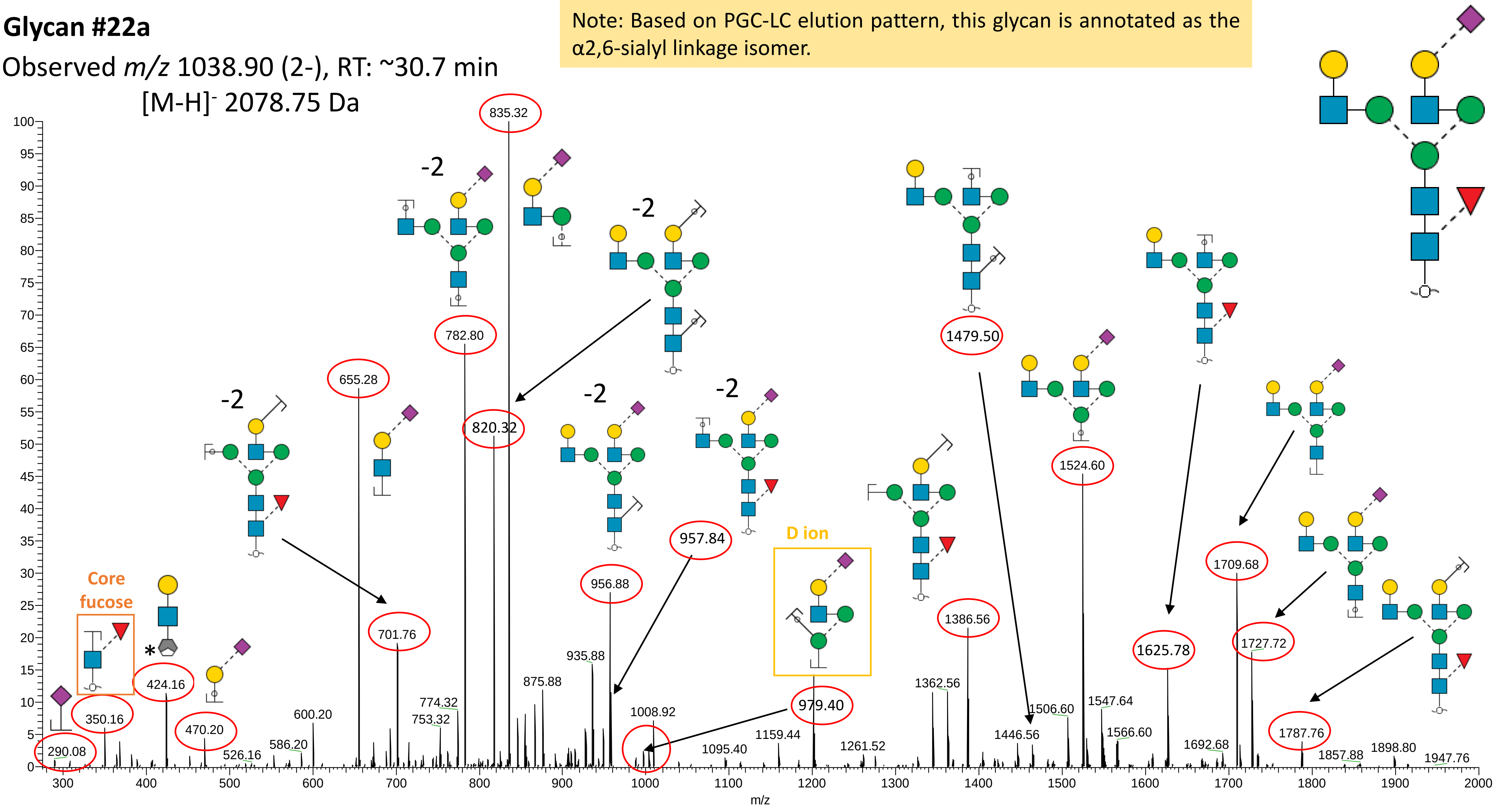

*Ambiguity of the cross-ring fragments

Glycan #22b

Observed *m/z* 1038.90 (2-), RT: ~39.3 min

[M-H]⁻ 2078.75 Da

Note: Based on PGC-LC elution pattern, this glycan is annotated as the α2,3-sialyl linkage isomer.

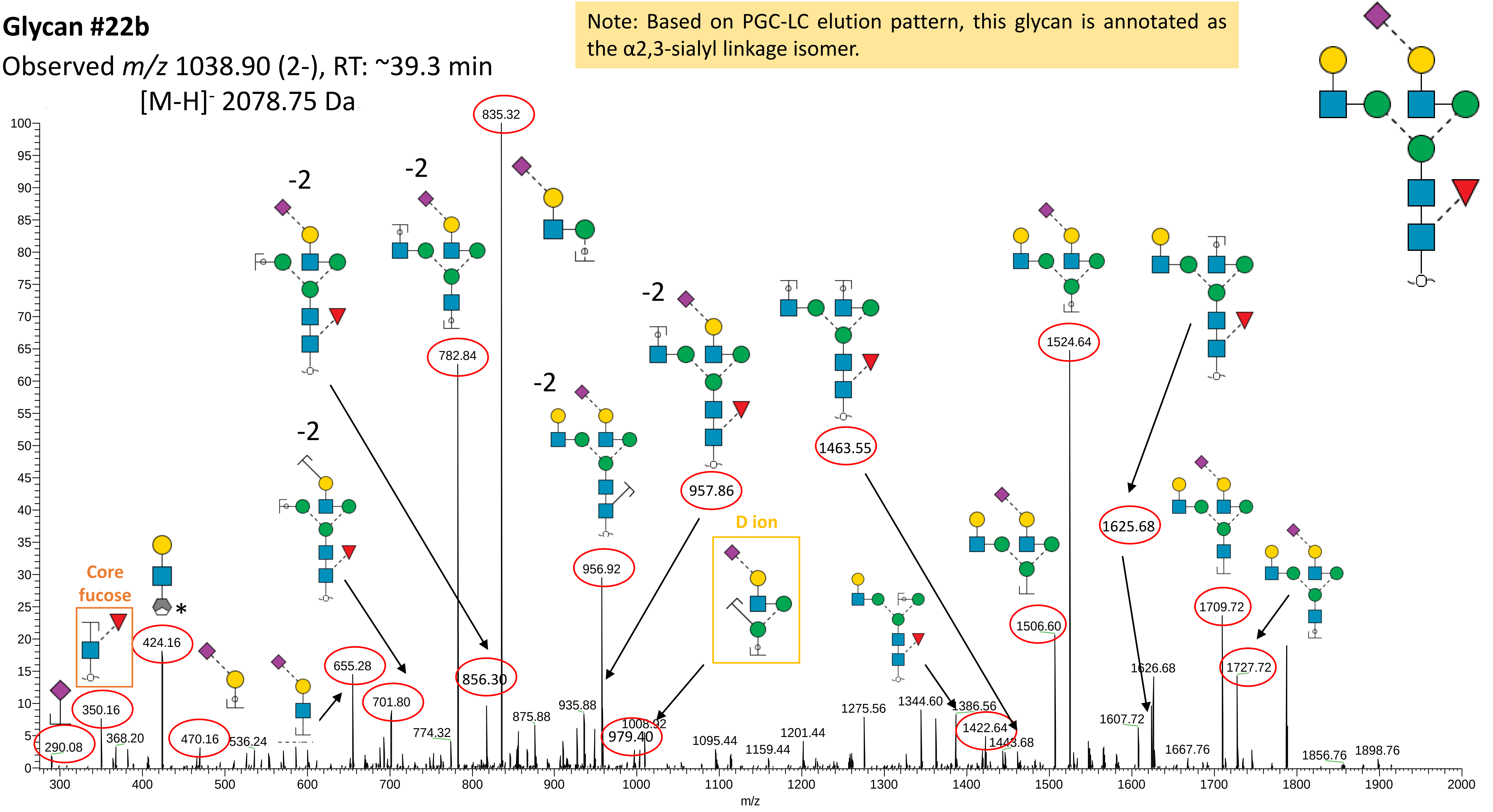

*Ambiguity of the cross-ring fragments

Glycan #23a

Observed *m/z* 1111.44 (2-), RT: ~28.8 min

$[M-H]^-$ 2223.79 Da

Note: Based on PGC-LC elution pattern, this glycan is annotated as the α2,6-α2,6-sialyl linkage isomer.

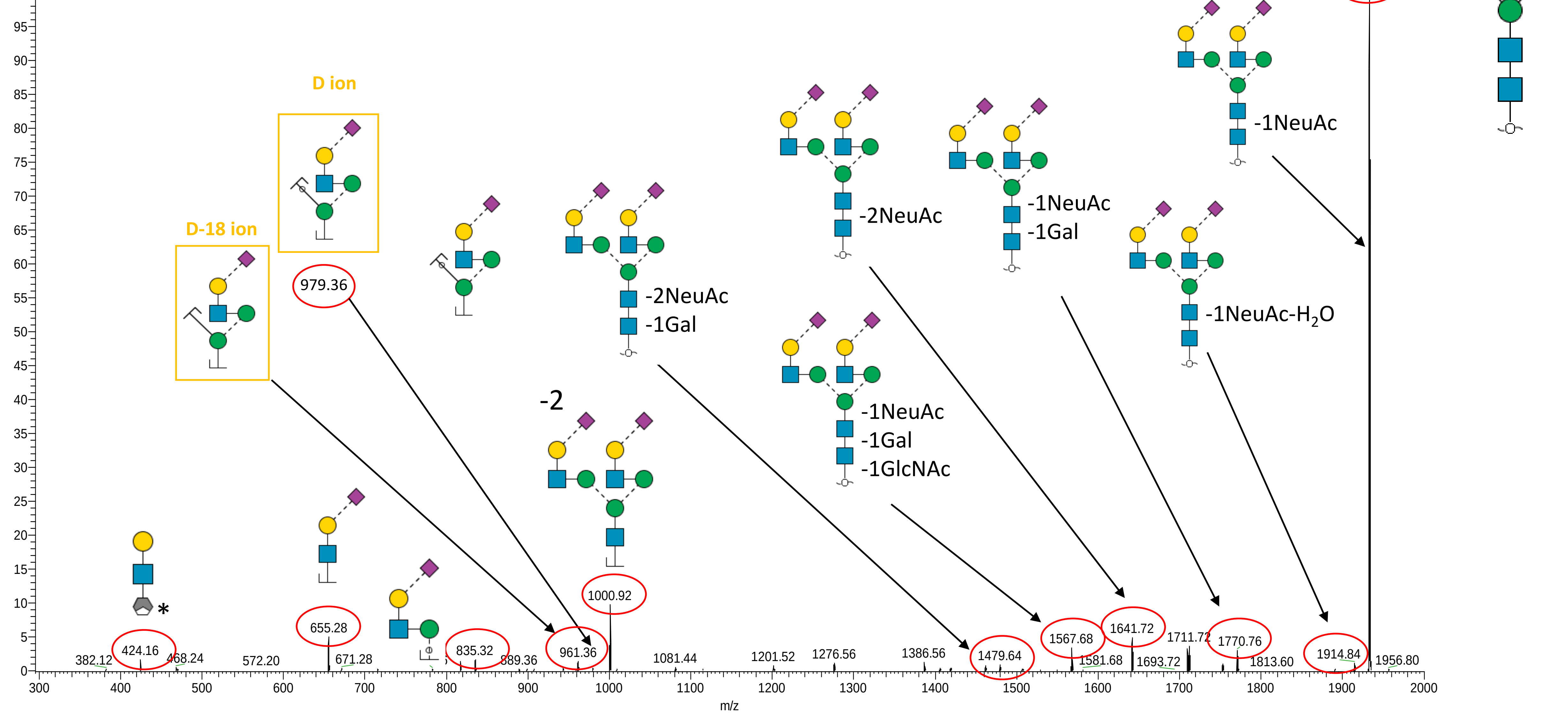

*Ambiguity of the cross-ring fragments

Glycan #23b

Observed *m/z* 1111.44 (2-), RT: ~38.8 min

$[M-H]^-$ 2223.79 Da

Note: Based on PGC-LC elution pattern, this glycan is annotated as the α2,6-α2,3-sialyl linkage isomer. The antenna positions of the linkage-specific sialic acid residues cannot be determined.

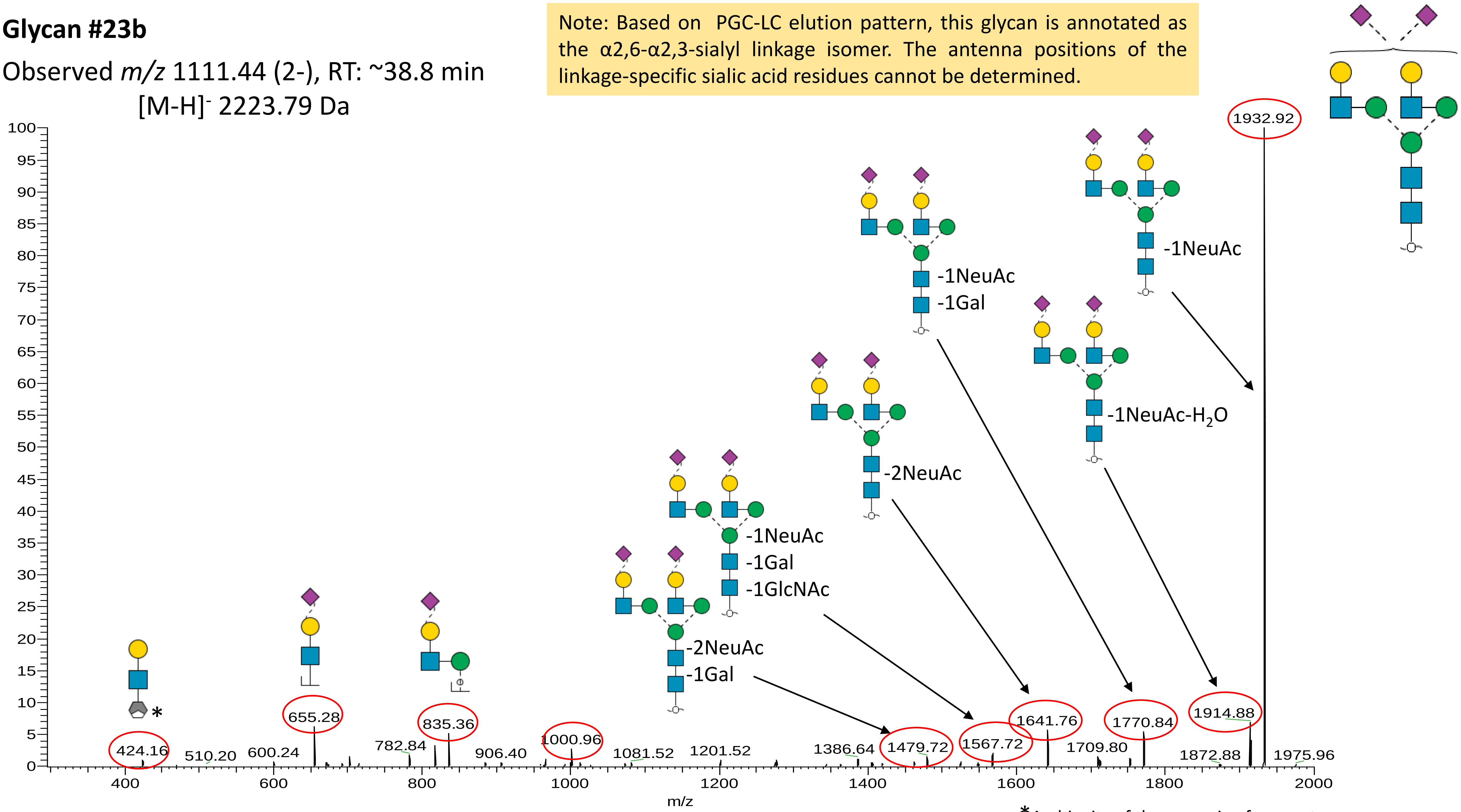

*Ambiguity of the cross-ring fragments

Glycan #24a

Observed *m/z* 1184.46 (2-), RT: ~36.4 min

[M-H]$^-$ 2369.84 Da

Note: Based on the PGC-LC elution pattern, this glycan is annotated as the α2,6-α2,6-sialyl linkage isomer.

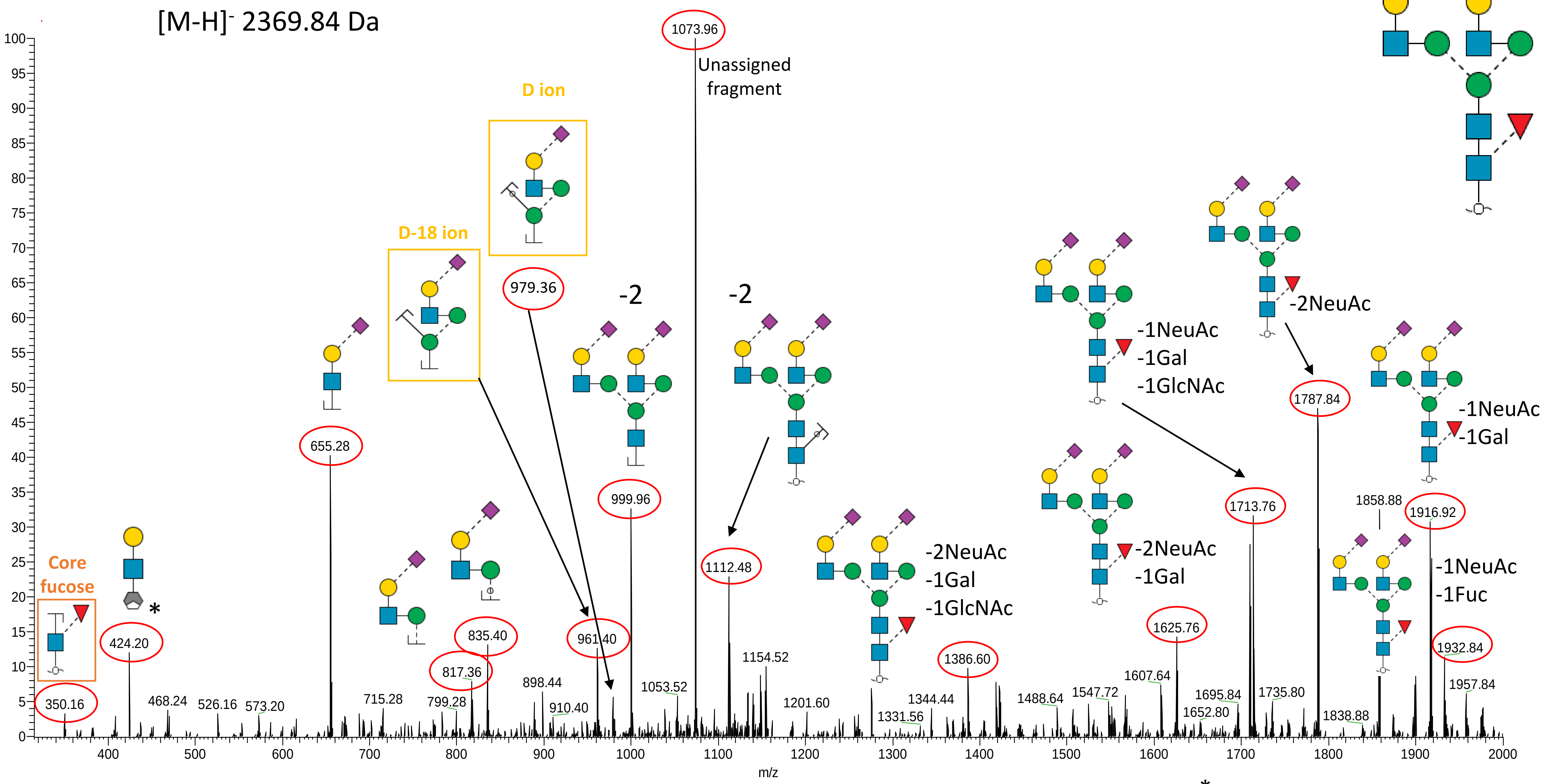

*Ambiguity of the cross-ring fragments

Glycan #24b

Observed *m/z* 1184.46 (2-), RT: ~43.9 min

[M-H]- 2369.84 Da

Note: Based on the PGC-LC elution pattern, this glycan is annotated as the α2,6-α2,3-sialyl linkage isomer. The antenna positions of the linkage-specific sialic acid residues cannot be determined.

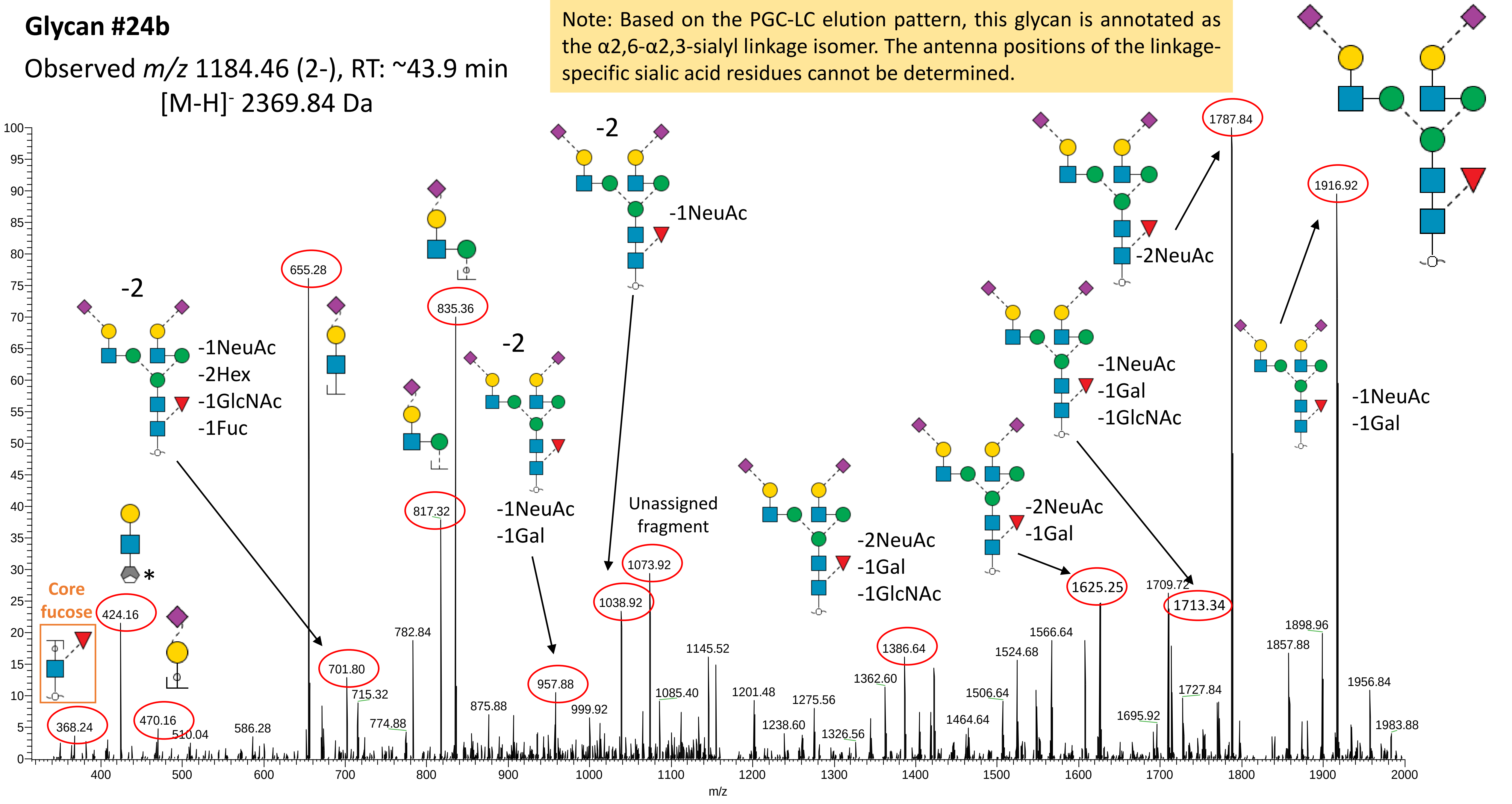

*Ambiguity of the cross-ring fragments

Glycan #24c

Observed *m/z* 1184.46 (2-), RT: ~44.4 min

$[M\text{-}H]^-$ 2369.84 Da

Note: Based on the PGC-LC elution pattern, this glycan is annotated as the α2,3-α2,6-sialyl linkage isomer. The antenna positions of the linkage-specific sialic acid residues cannot be determined.

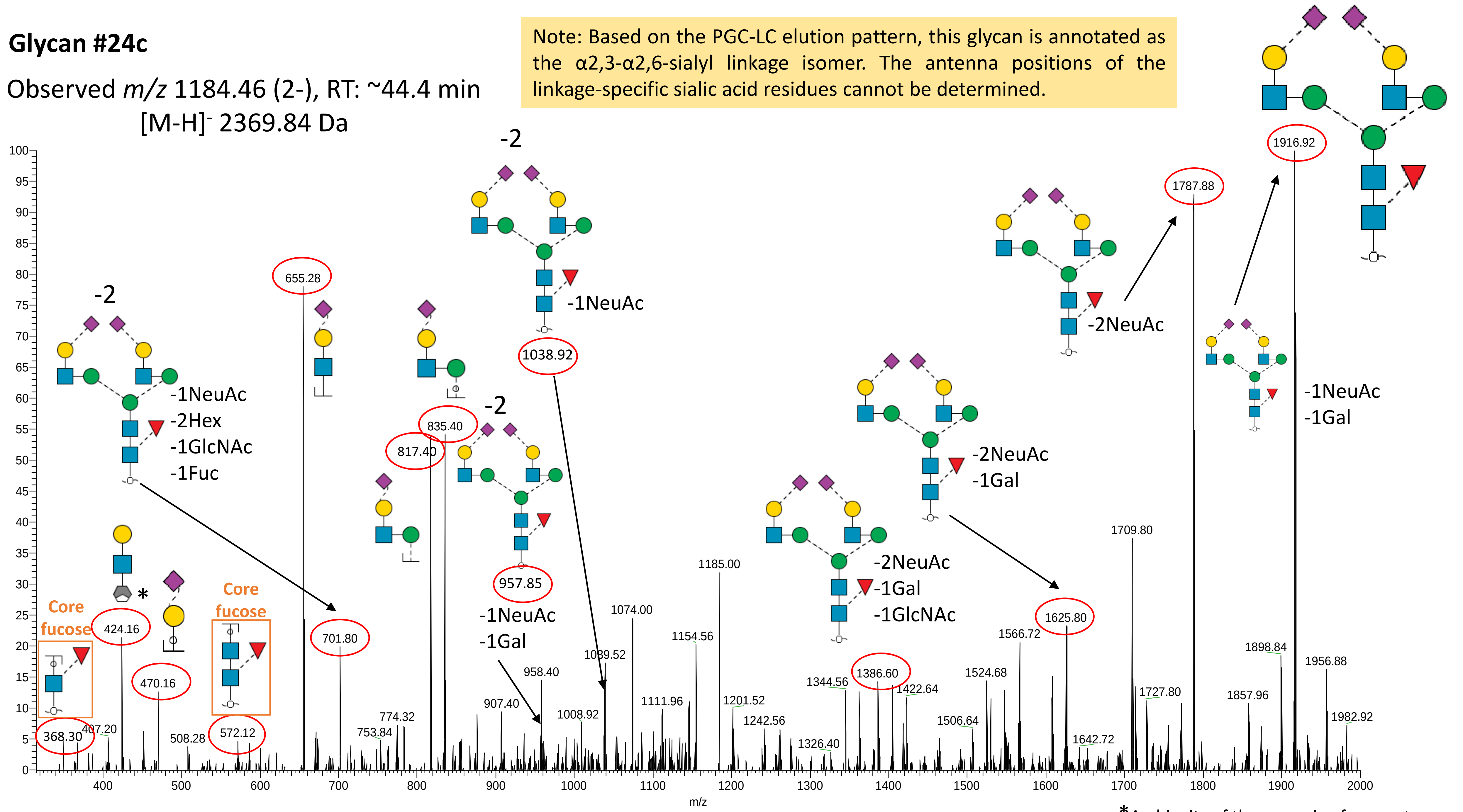

*Ambiguity of the cross-ring fragments

Glycan #24d

Observed *m/z* 1184.46 (2-), RT: ~51.8 min

[M-H]⁻ 2369.84 Da

Note: Based on the PGC-LC elution pattern and the presence of four isomers, this glycan is annotated as the α2,3-α2,3-sialyl linkage isomer.

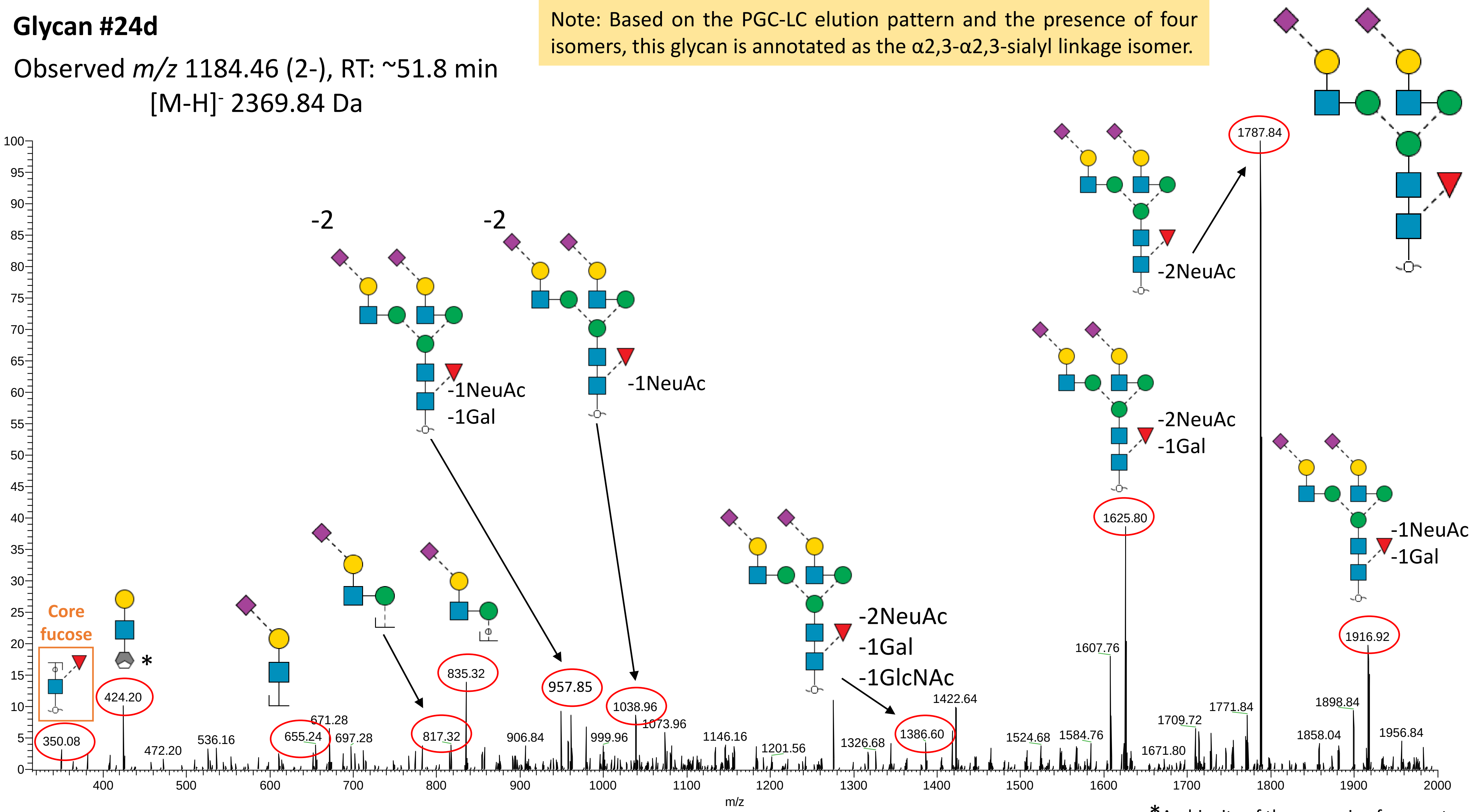

*Ambiguity of the cross-ring fragments

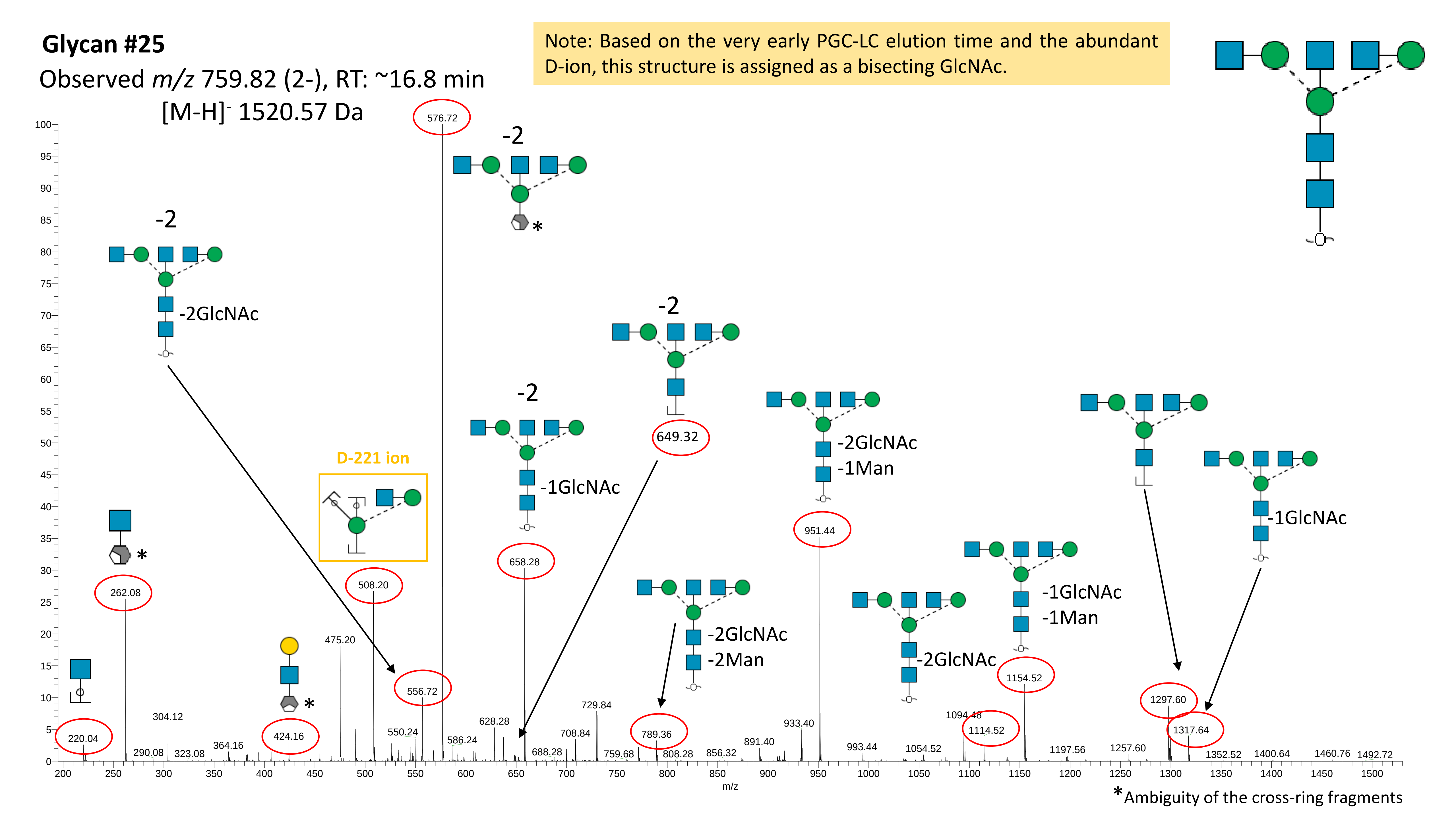

Glycan #25
Observed m/z 759.82 (2-), RT: ~16.8 min
[M-H]- 1520.57 Da
Note: Based on the very early PGC-LC elution time and the abundant D-ion, this structure is assigned as a bisecting GlcNAc.
D-221 ion
-2
-2GlcNAc
-1GlcNAc
-2GlcNAc
-1Man
-2GlcNAc
-2Man
-1GlcNAc
-1Man
*
220.04
262.08
290.08
304.12
323.08
364.16
424.16
475.20
508.20
550.24
556.72
576.72
586.24
628.28
649.32
658.28
688.28
708.84
729.84
759.68
789.36
808.28
856.32
891.40
933.40
951.44
993.44
1054.52
1094.48
1114.52
1154.52
1197.56
1257.60
1297.60
1317.64
1352.52
1400.64
1460.76
1492.72
m/z
*Ambiguity of the cross-ring fragments

Glycan #26

Observed *m/z* 832.81 (2-), RT: ~19.7 min

[M-H]⁻ 1666.63 Da

Note: Based on the very early PGC-LC elution time and the abundant D-ion, this structure is assigned as a bisecting GlcNAc.

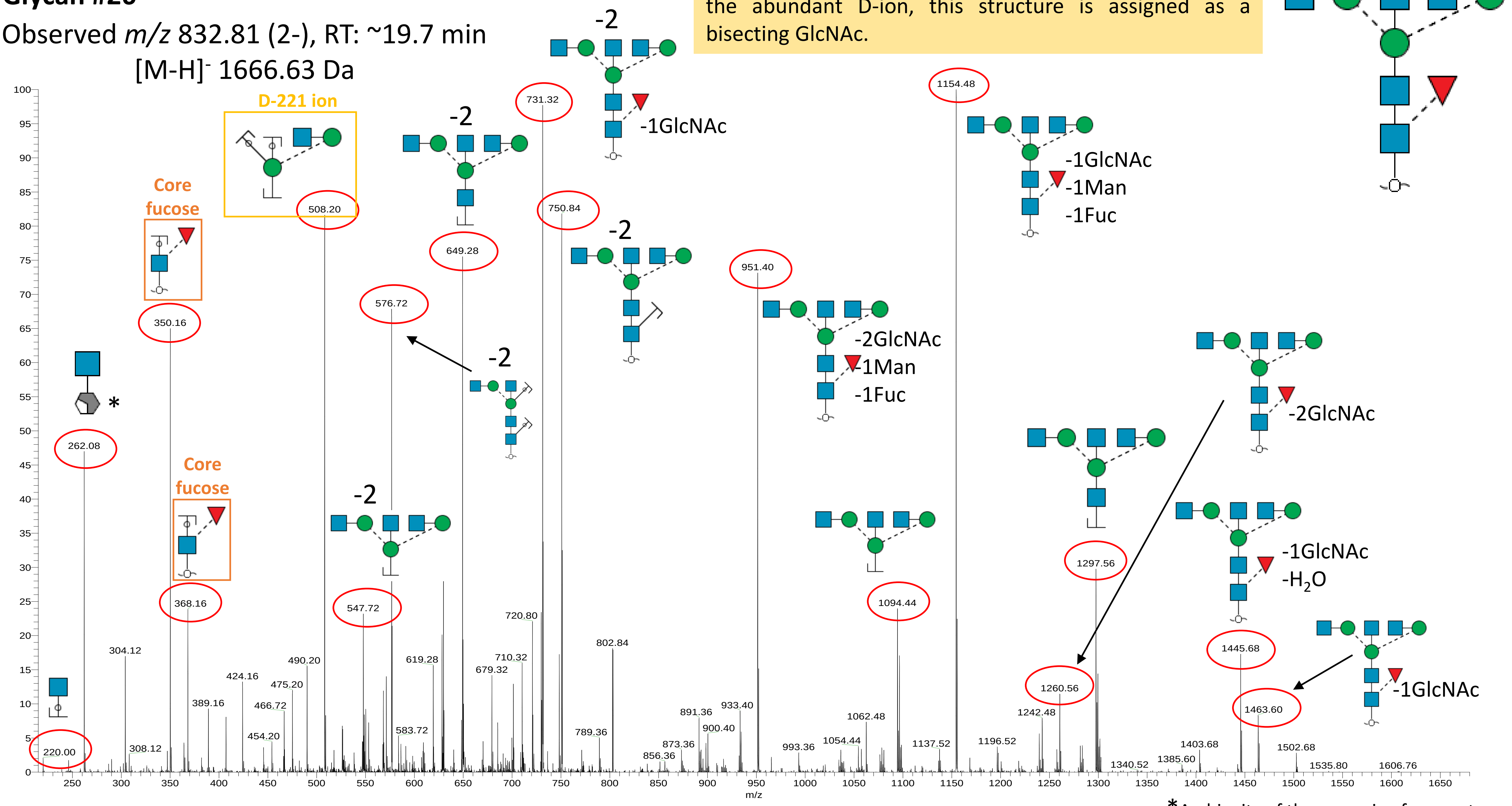

*Ambiguity of the cross-ring fragments

Glycan #27

Observed *m/z* 840.84 (2-), RT: ~17.8 min

[M-H]⁻ 1682.62 Da

Note: Based on the very early PGC-LC elution time and the abundant D-ion, this structure is assigned as a bisecting GlcNAc.

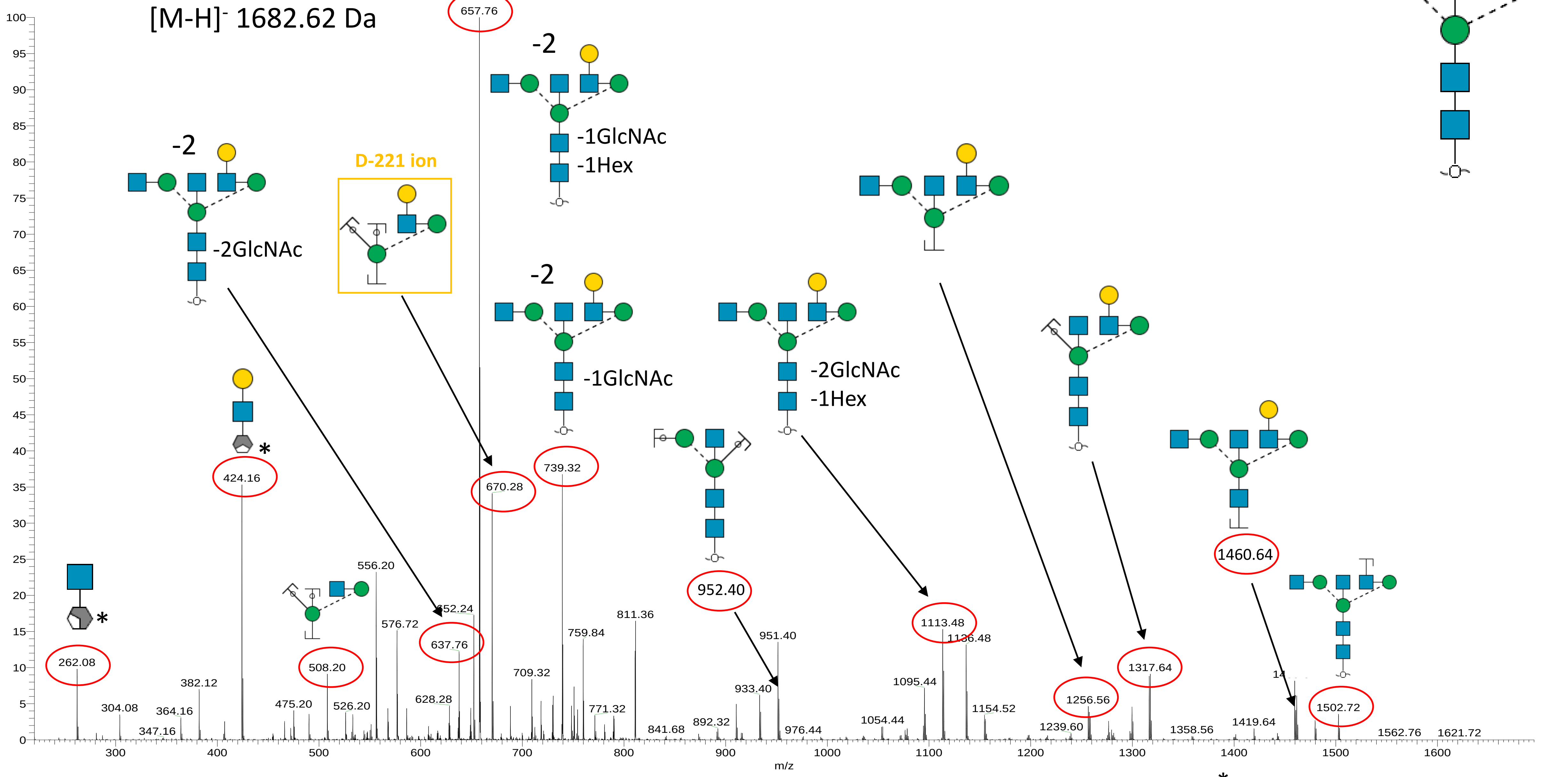

*Ambiguity of the cross-ring fragments

Glycan #28

Observed *m/z* 913.86 (2-), RT: ~20.8 min

$[M-H]^-$ 1828.68 Da

Note: Based on the very early PGC-LC elution time and the abundant D-ion, this structure is assigned as a bisecting GlcNAc.

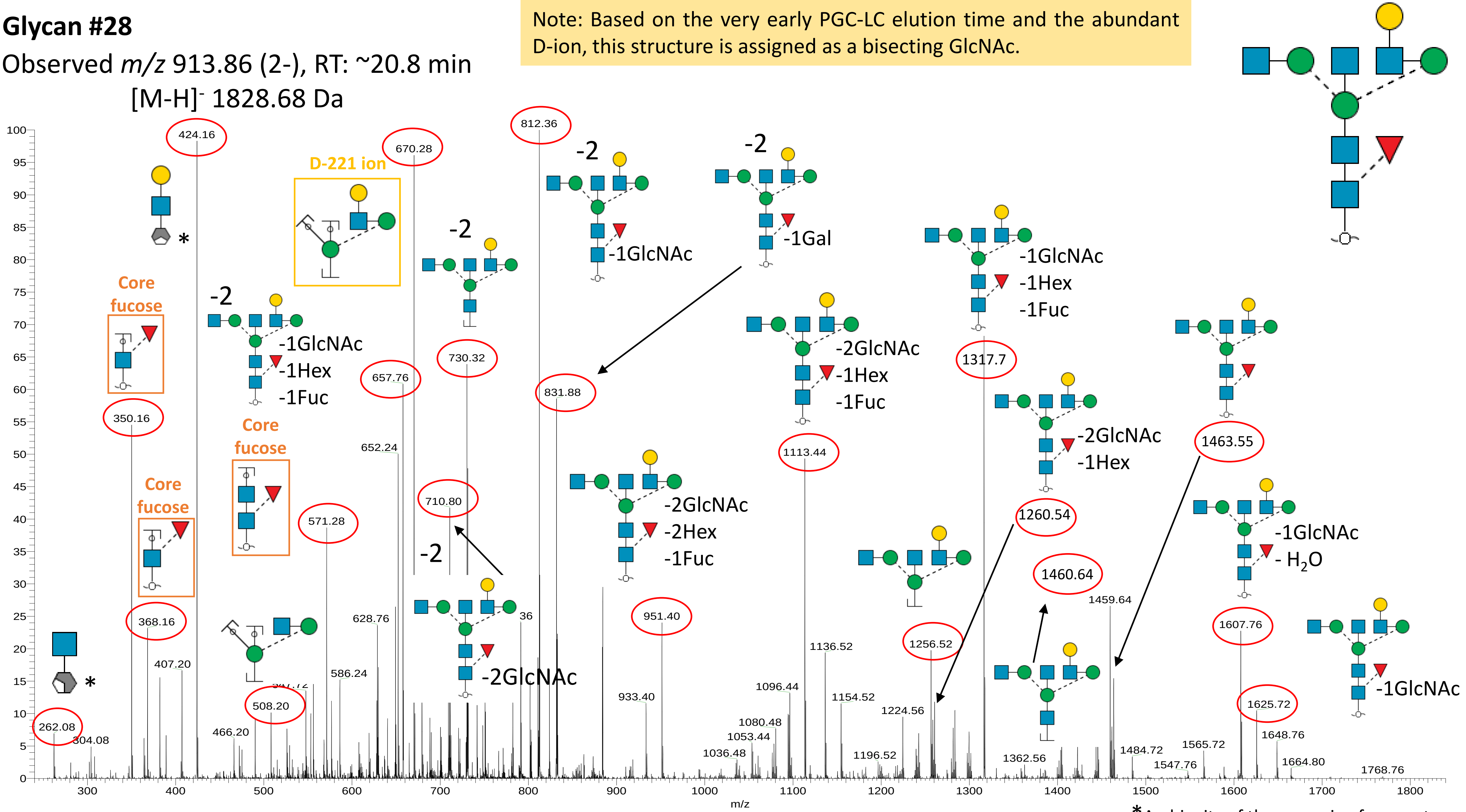

*Ambiguity of the cross-ring fragments

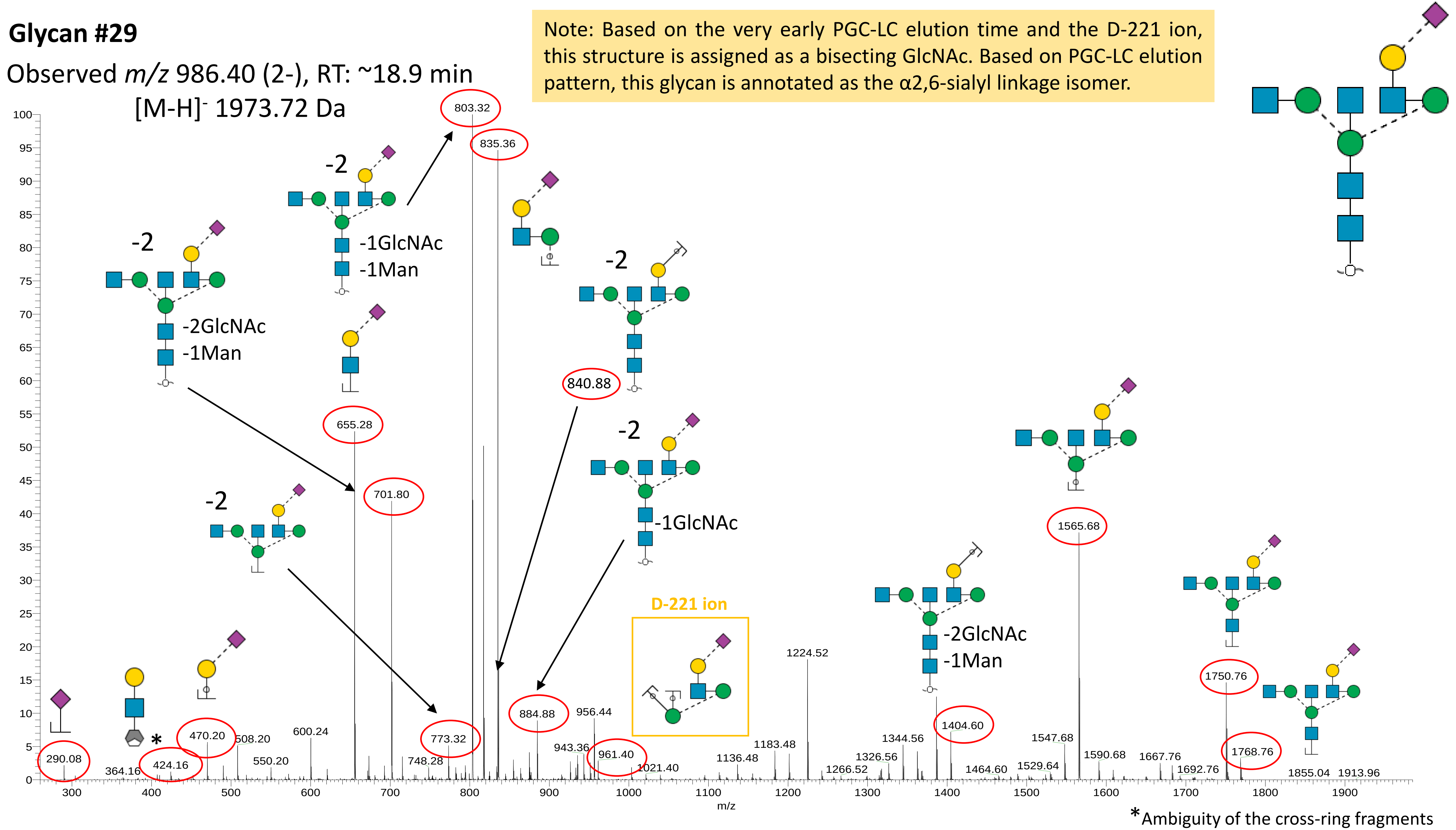

Glycan #29
Observed m/z 986.40 (2-), RT: ~18.9 min
[M-H]- 1973.72 Da
Note: Based on the very early PGC-LC elution time and the D-221 ion, this structure is assigned as a bisecting GlcNAc. Based on PGC-LC elution pattern, this glycan is annotated as the α2,6-sialyl linkage isomer.
-2
-1GlcNAc
-1Man
-2GlcNAc
-1Man
-1GlcNAc
-2GlcNAc
-1Man
D-221 ion
803.32
835.36
840.88
655.28
701.80
1565.68
1224.52
1750.76
1404.60
884.88
956.44
773.32
470.20
508.20
600.24
290.08
364.16
424.16
550.20
748.28
943.36
961.40
1021.40
1136.48
1183.48
1266.52
1326.56
1344.56
1464.60
1529.64
1547.68
1590.68
1667.76
1692.76
1768.76
1855.04
1913.96
m/z
*Ambiguity of the cross-ring fragments

Glycan #30

Observed *m/z* 1059.42 (2-), RT: ~22.5 min

[M-H]- 2119.78 Da

Note: Based on the very early PGC-LC elution time and the abundant D-221 ion, this structure is assigned as a bisecting GlcNAc. Based on PGC-LC elution pattern, this glycan is annotated as the α2,6-sialyl linkage isomer.

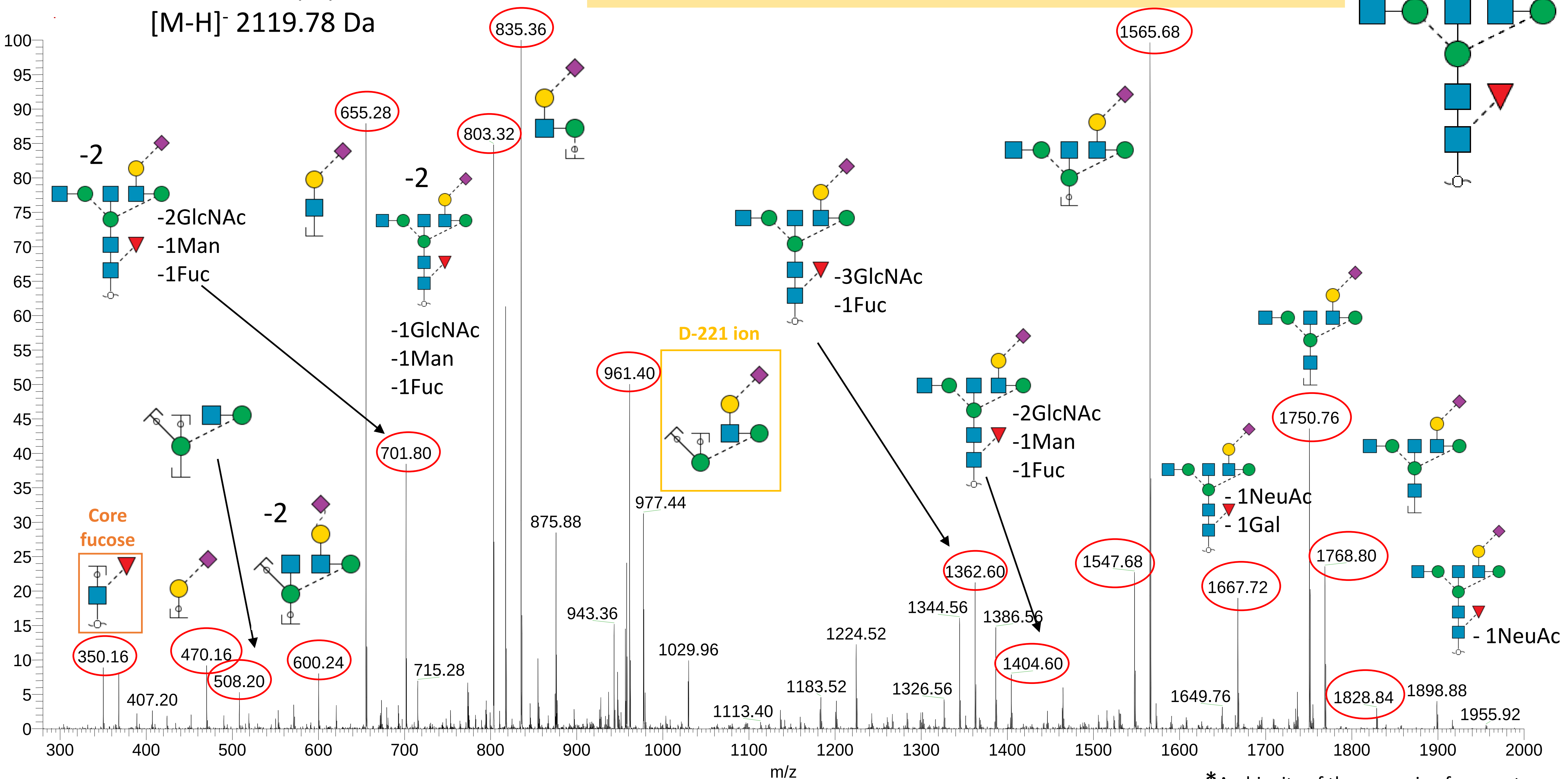

*Ambiguity of the cross-ring fragments

Glycan #31

Observed *m/z* 994.88 (2-), RT: ~22.2 min

$[M-H]^{-}$ 1990.73 Da

Note: Based on the very early PGC-LC elution time and the abundant D-221 ion, this structure is assigned as a bisecting GlcNAc.

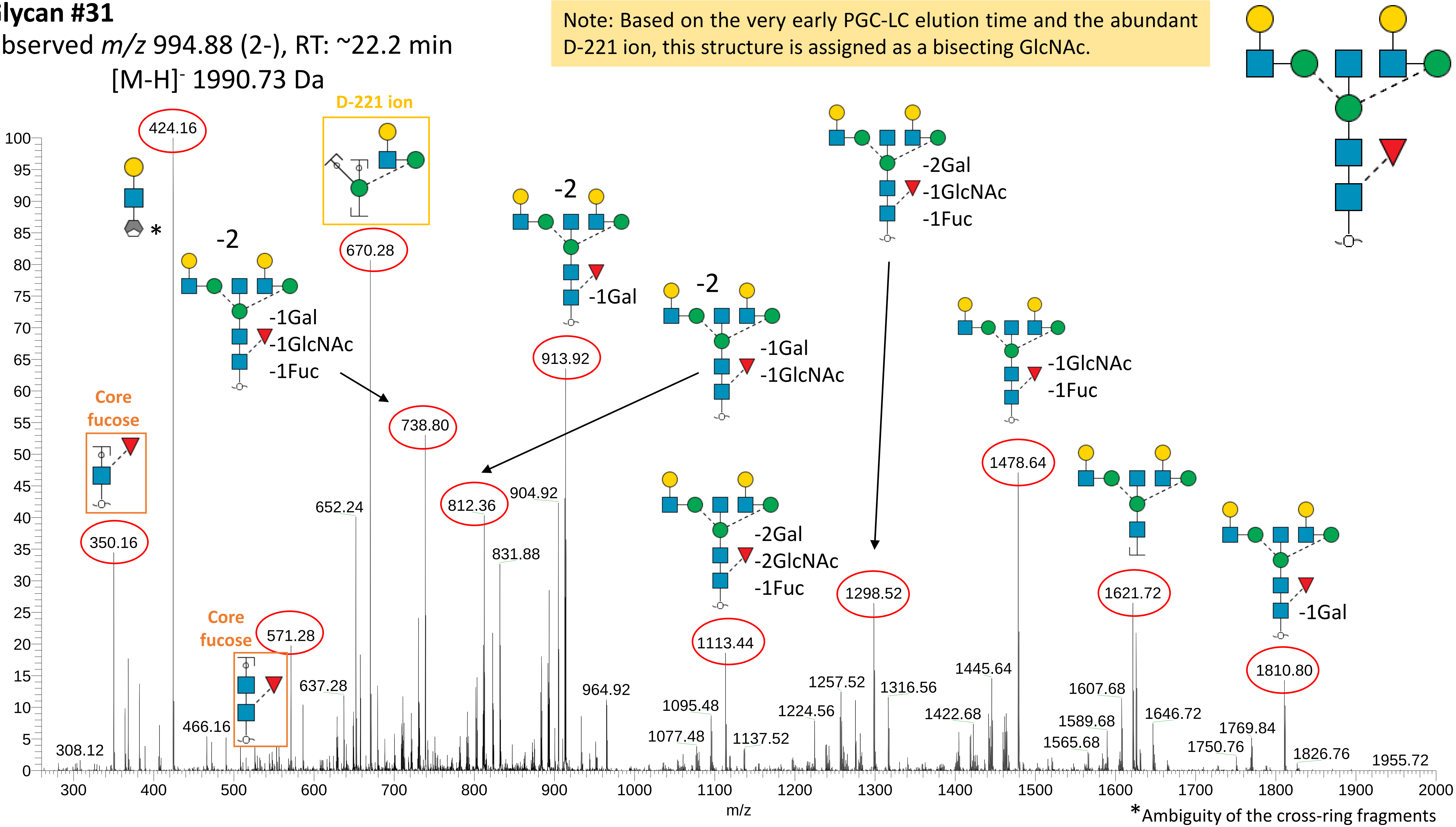

*Ambiguity of the cross-ring fragments

Glycan #32

Observed *m/z* 1067.42 (2-), RT: ~20.5 min

[M-H]⁻ 2135.77 Da

Note: Based on the very early PGC-LC elution time and the D-221 ion, this structure is assigned as a bisecting GlcNAc with sialylation, predicted to occupy the α-1,3 arm. Based on PGC-LC elution pattern, this glycan is annotated as the α2,6-sialyl linkage isomer.

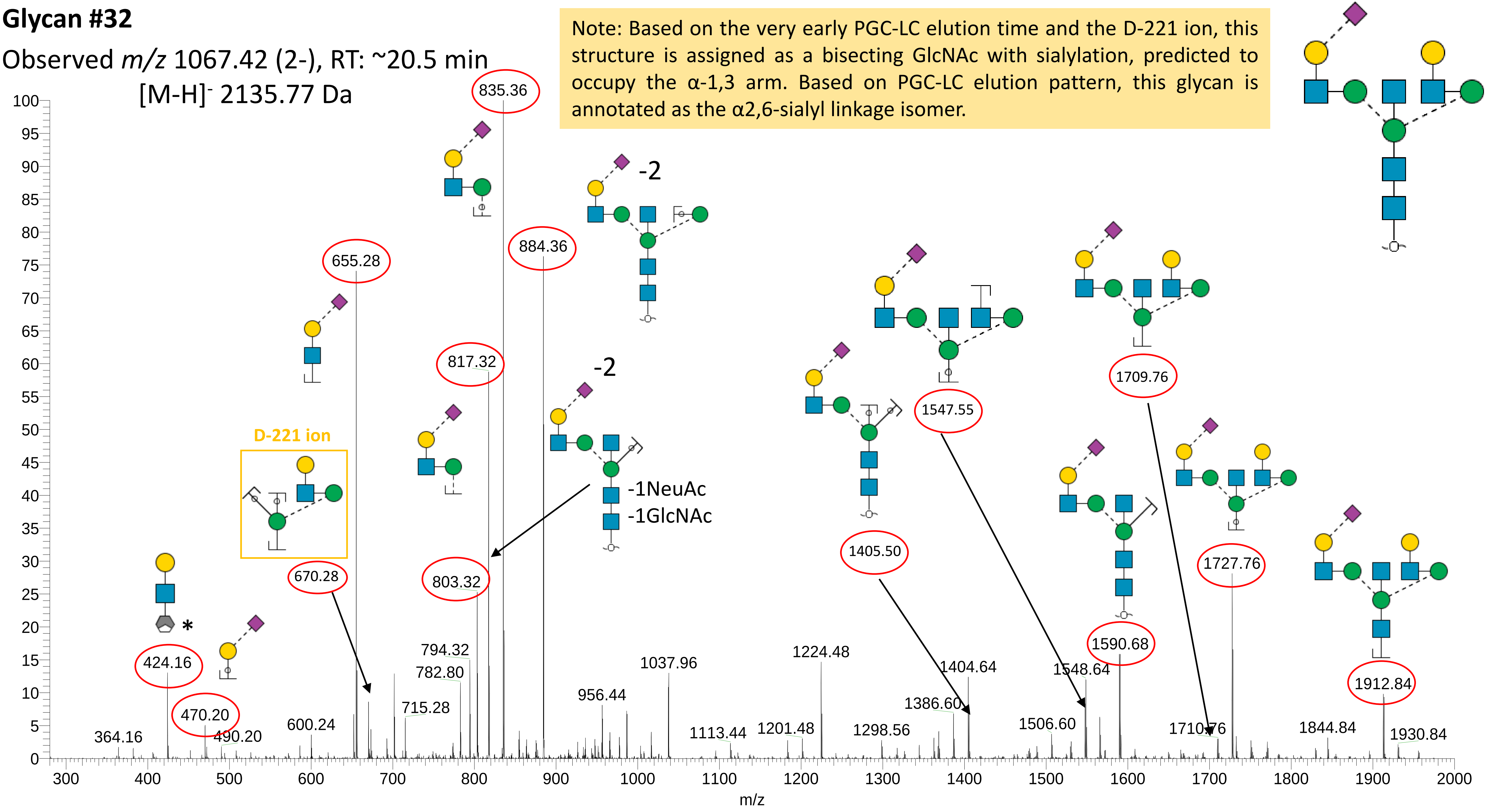

*Ambiguity of the cross-ring fragments

Glycan #33

Observed *m/z* 1140.44 (2-), RT: ~23.7 min

[M-H]- 2135.77 Da

Note: Based on the very early PGC-LC elution time and the D-221 ion, this structure is assigned as a bisecting GlcNAc with sialylation, predicted to occupy the α-1,3 arm. Based on PGC-LC elution pattern, this glycan is annotated as the α2,6-sialyl linkage isomer.

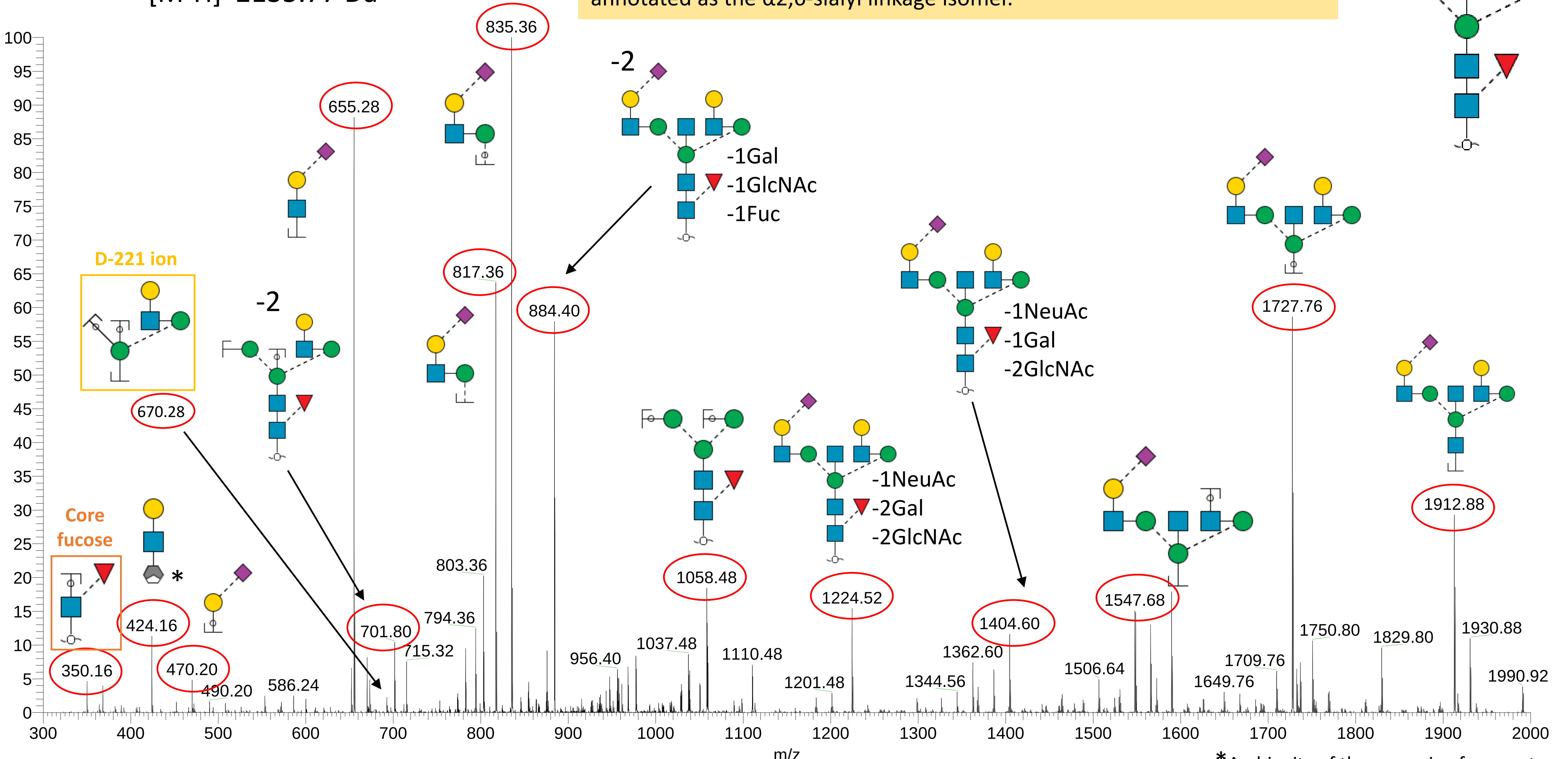

*Ambiguity of the cross-ring fragments

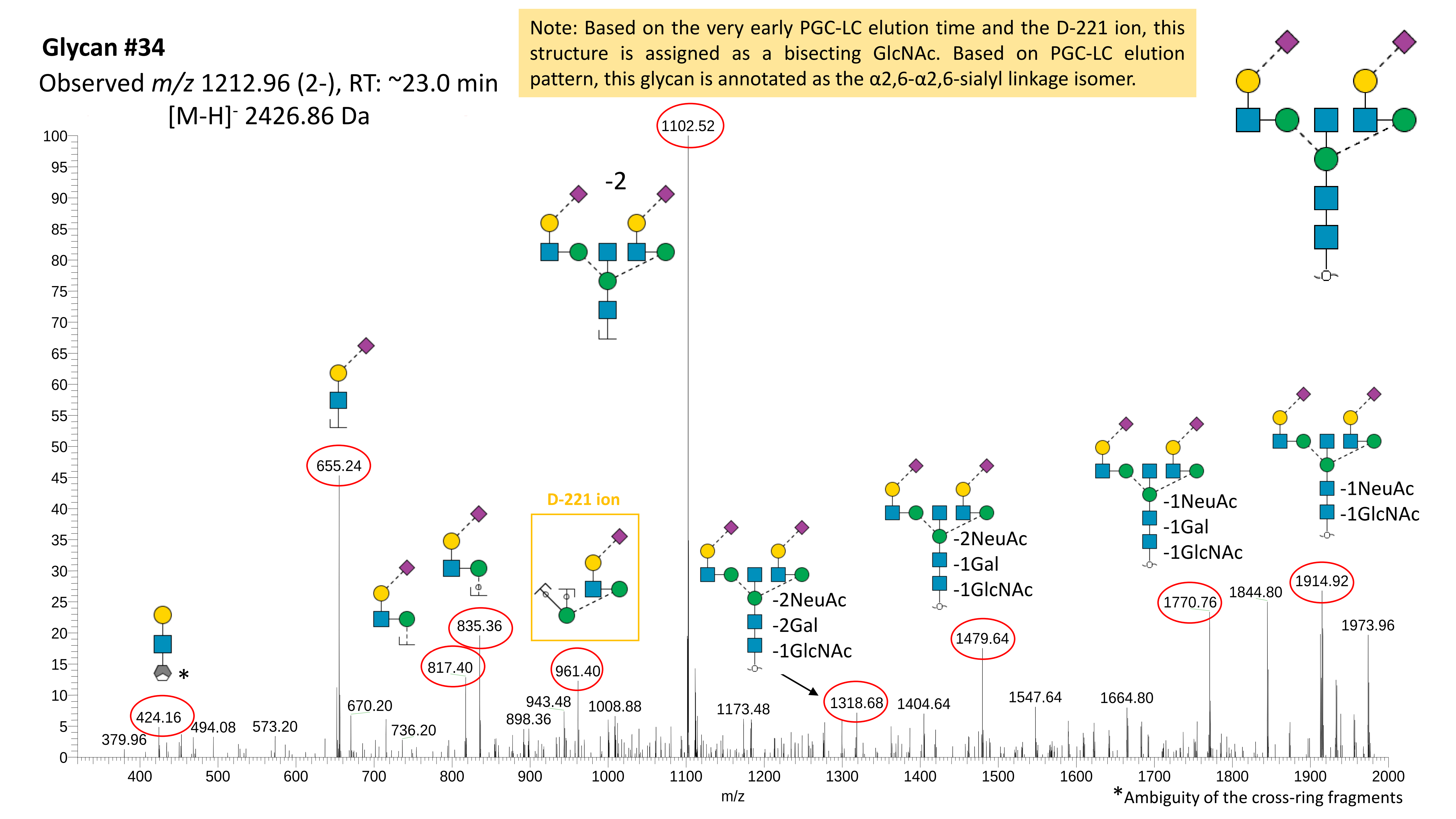

Glycan #34
Observed m/z 1212.96 (2-), RT: ~23.0 min
[M-H]- 2426.86 Da
Note: Based on the very early PGC-LC elution time and the D-221 ion, this structure is assigned as a bisecting GlcNAc. Based on PGC-LC elution pattern, this glycan is annotated as the α2,6-α2,6-sialyl linkage isomer.
-2
1102.52
655.24
D-221 ion
835.36
817.40
961.40
424.16
379.96
494.08
573.20
670.20
736.20
898.36
943.48
1008.88
1173.48
1318.68
1404.64
1479.64
1547.64
1664.80
1770.76
1844.80
1914.92
1973.96
-2NeuAc
-2Gal
-1GlcNAc
-2NeuAc
-1Gal
-1GlcNAc
-1NeuAc
-1Gal
-1GlcNAc
-1NeuAc
-1GlcNAc
m/z
*Ambiguity of the cross-ring fragments

Glycan #35

Observed *m/z* 1286.02 (2-), RT: ~25.1 min

[M-H]$^-$ 2572.92 Da

Note: Based on the very early PGC-LC elution time and the D-221 ion, this structure is assigned as a bisecting GlcNAc. Based on PGC-LC elution pattern, this glycan is annotated as the α2,6-α2,6-sialyl linkage isomer.

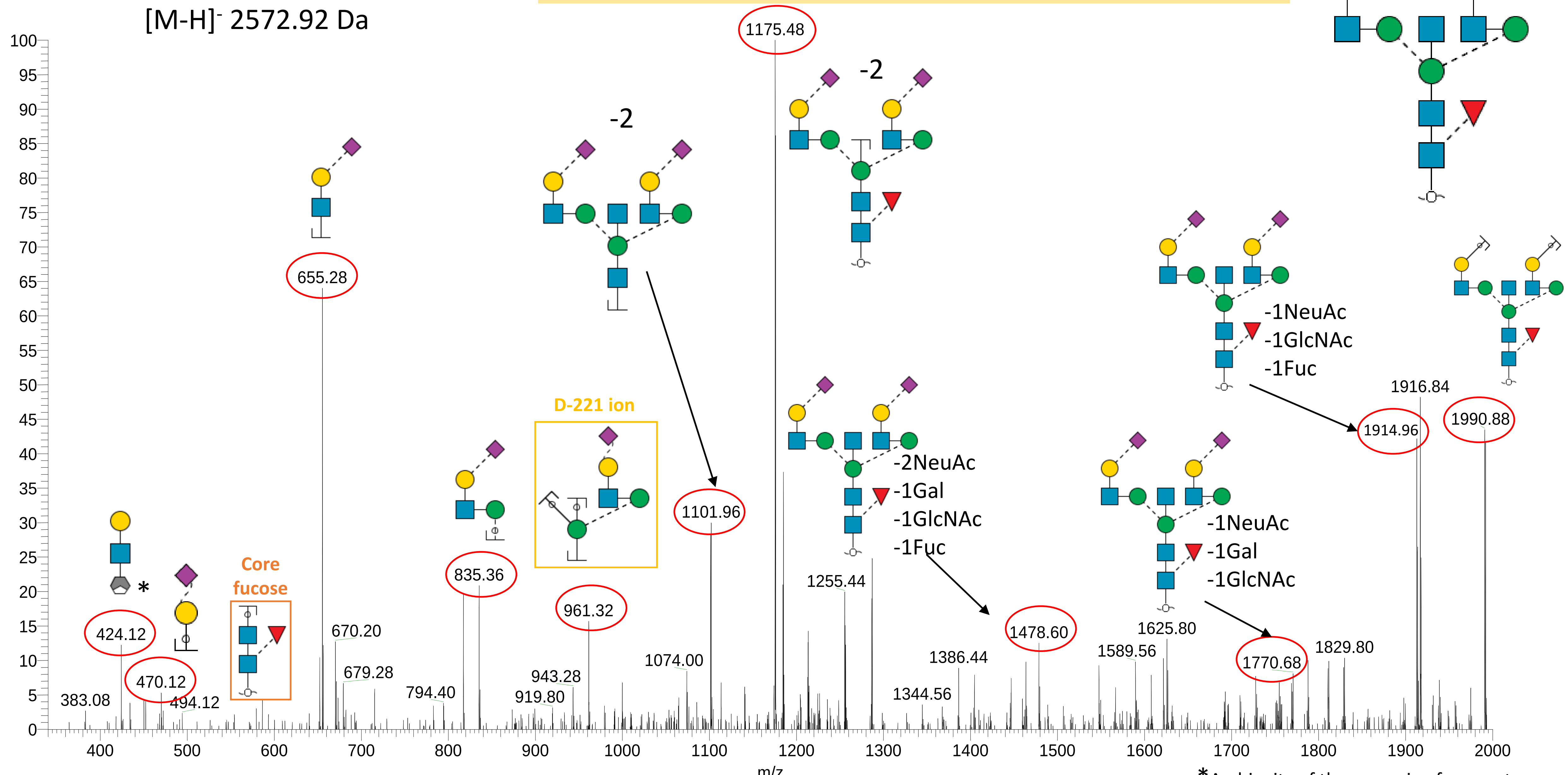

*Ambiguity of the cross-ring fragments

Glycan #36a

Observed *m/z* 1148.44 (2-), RT: ~28.5 min

$[M-H]^-$ 2297.82 Da

Note: Based on PGC-LC elution pattern, this glycan is annotated as the α2,6-sialyl linkage isomer but the exact position cannot be determined. No D ion observed to allocate antennary branching and sialic acid.

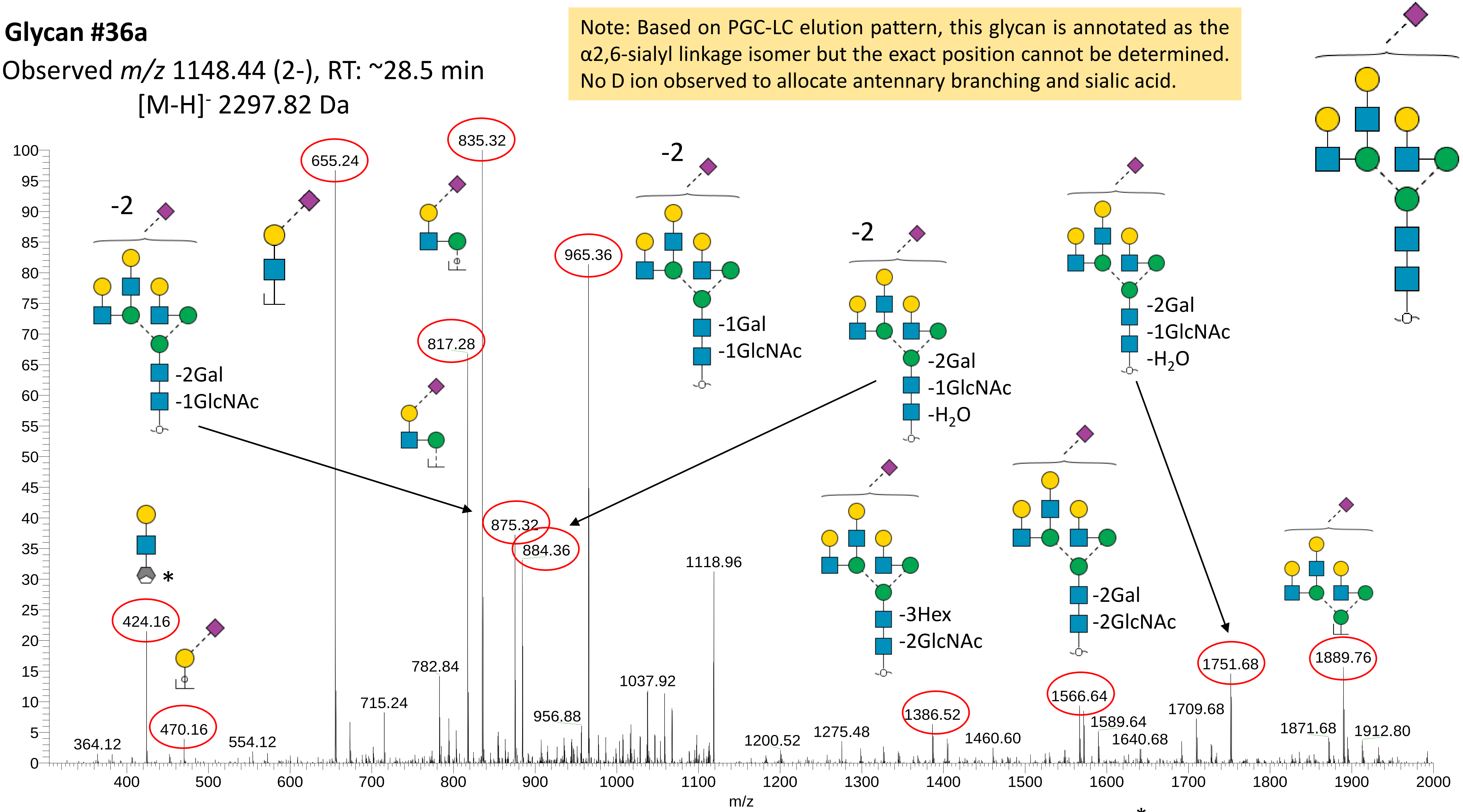

*Ambiguity of the cross-ring fragments

Glycan #36b

Observed *m/z* 1148.44 (2-), RT: ~33.6 min

[M-H]$^-$ 2297.82 Da

Note: Based on PGC-LC elution pattern, this glycan is annotated as the α2,3-sialyl linkage isomer but the exact position cannot be determined. No D ion observed to allocate antennary branching and sialic acid.

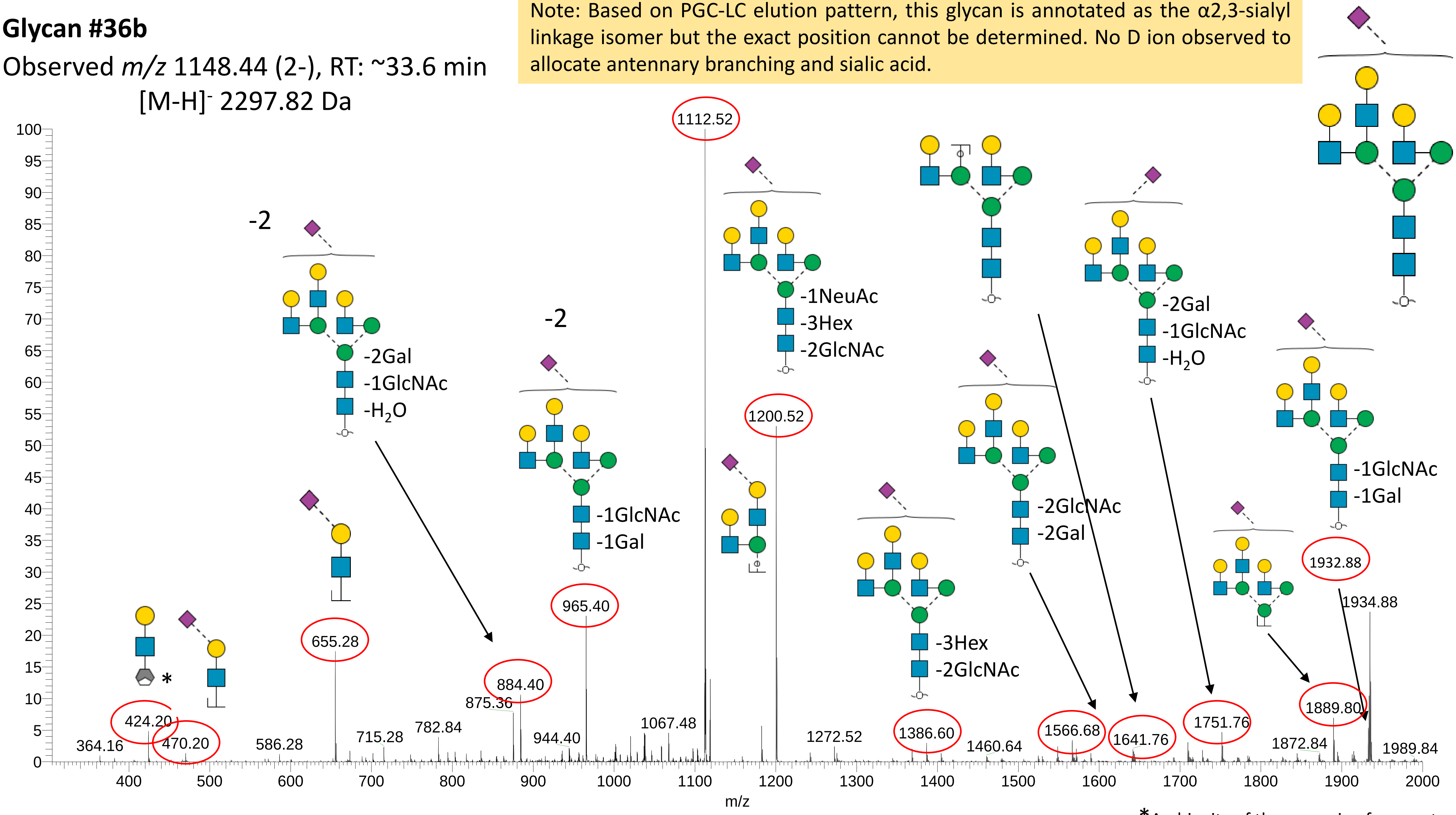

*Ambiguity of the cross-ring fragments

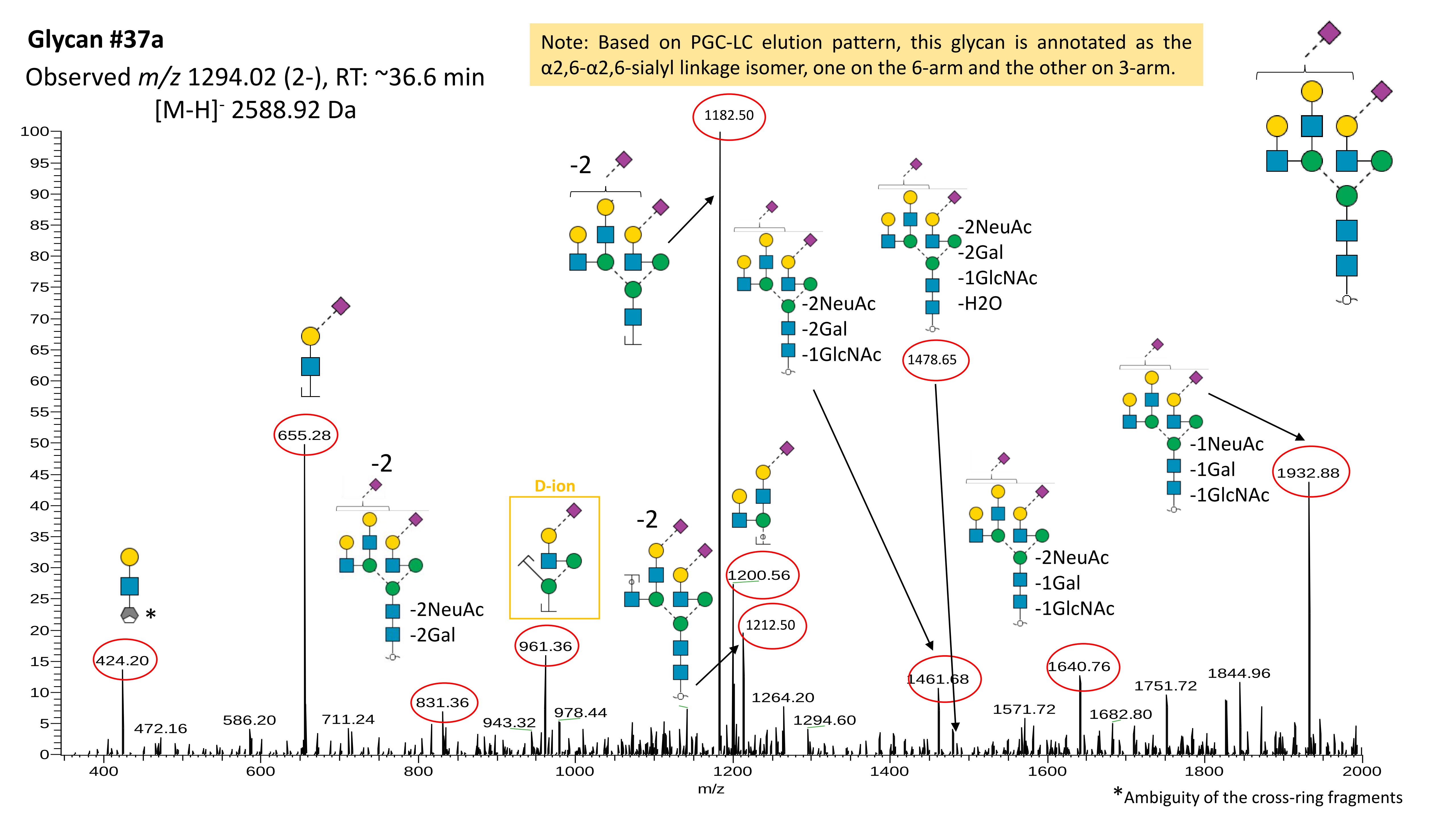
Glycan #37a
Observed m/z 1294.02 (2-), RT: ~36.6 min
[M-H]- 2588.92 Da
Note: Based on PGC-LC elution pattern, this glycan is annotated as the α2,6-α2,6-sialyl linkage isomer, one on the 6-arm and the other on 3-arm.
1182.50
-2
-2NeuAc
-2Gal
-1GlcNAc
-2NeuAc
-2Gal
-1GlcNAc
-H2O
1478.65
655.28
-2
-2NeuAc
-2Gal
D-ion
-2
1200.56
1212.50
-1NeuAc
-1Gal
-1GlcNAc
1932.88
-2NeuAc
-1Gal
-1GlcNAc
*
424.20
472.16
586.20
711.24
831.36
943.32
961.36
978.44
1264.20
1294.60
1461.68
1571.72
1640.76
1682.80
1751.72
1844.96
m/z
*Ambiguity of the cross-ring fragments

Glycan #37b

Observed *m/z* 1294.02 (2-), RT: ~37.3 min

[M-H]⁻ 2588.92 Da

Note: Based on PGC-LC elution pattern, this glycan is annotated as the α2,6-α2,3-sialyl linkage isomer, one on the 6-arm and the other on 3-arm.

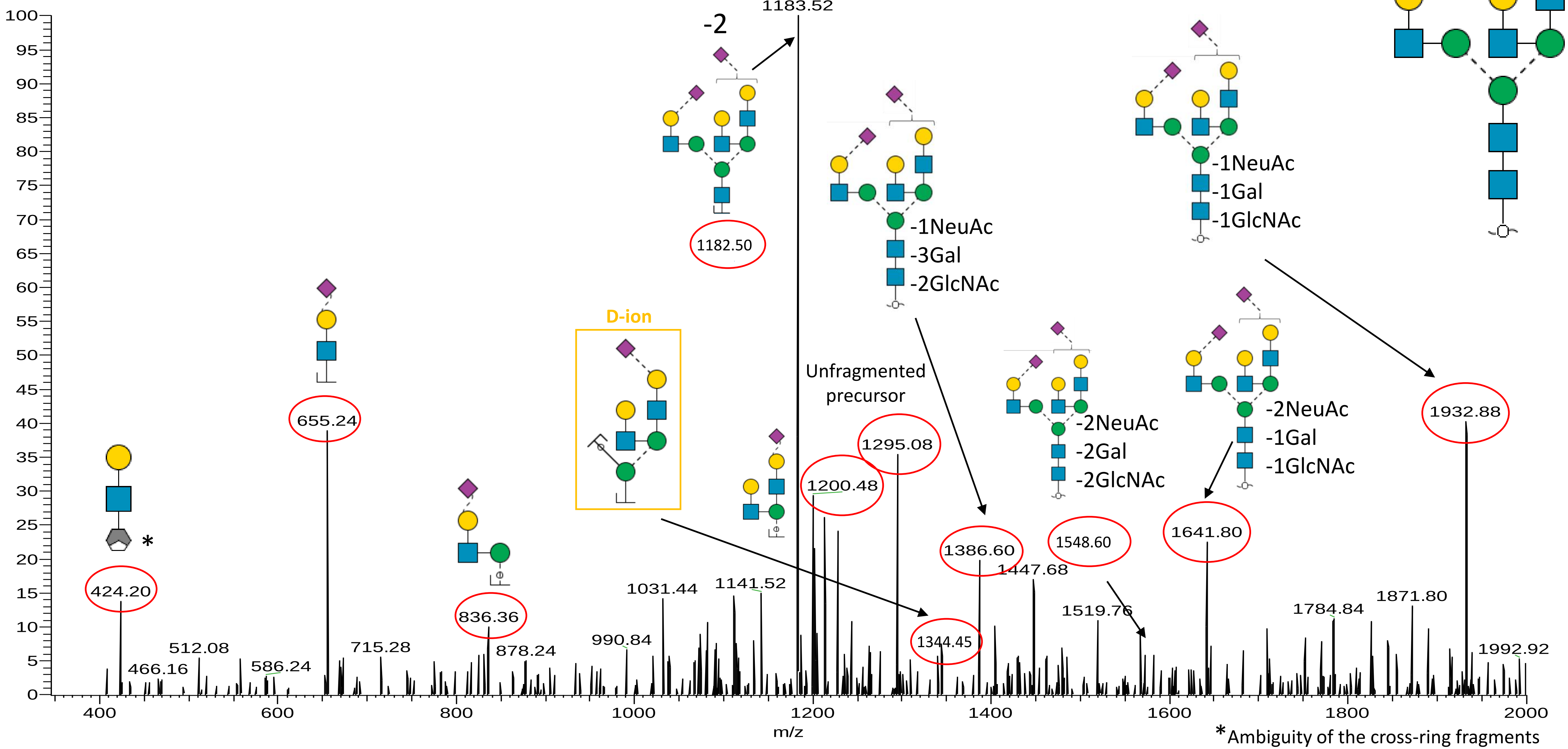

*Ambiguity of the cross-ring fragments

Glycan #37c

Observed *m/z* 1294.02 (2-), RT: ~42.1 min

$[M\text{-}H]^-$ 2588.92 Da

Note: Based on PGC-LC elution pattern, this glycan is annotated as the α2,3-α2,3-sialyl linkage isomer, one on the 6-arm and the other on 3-arm.

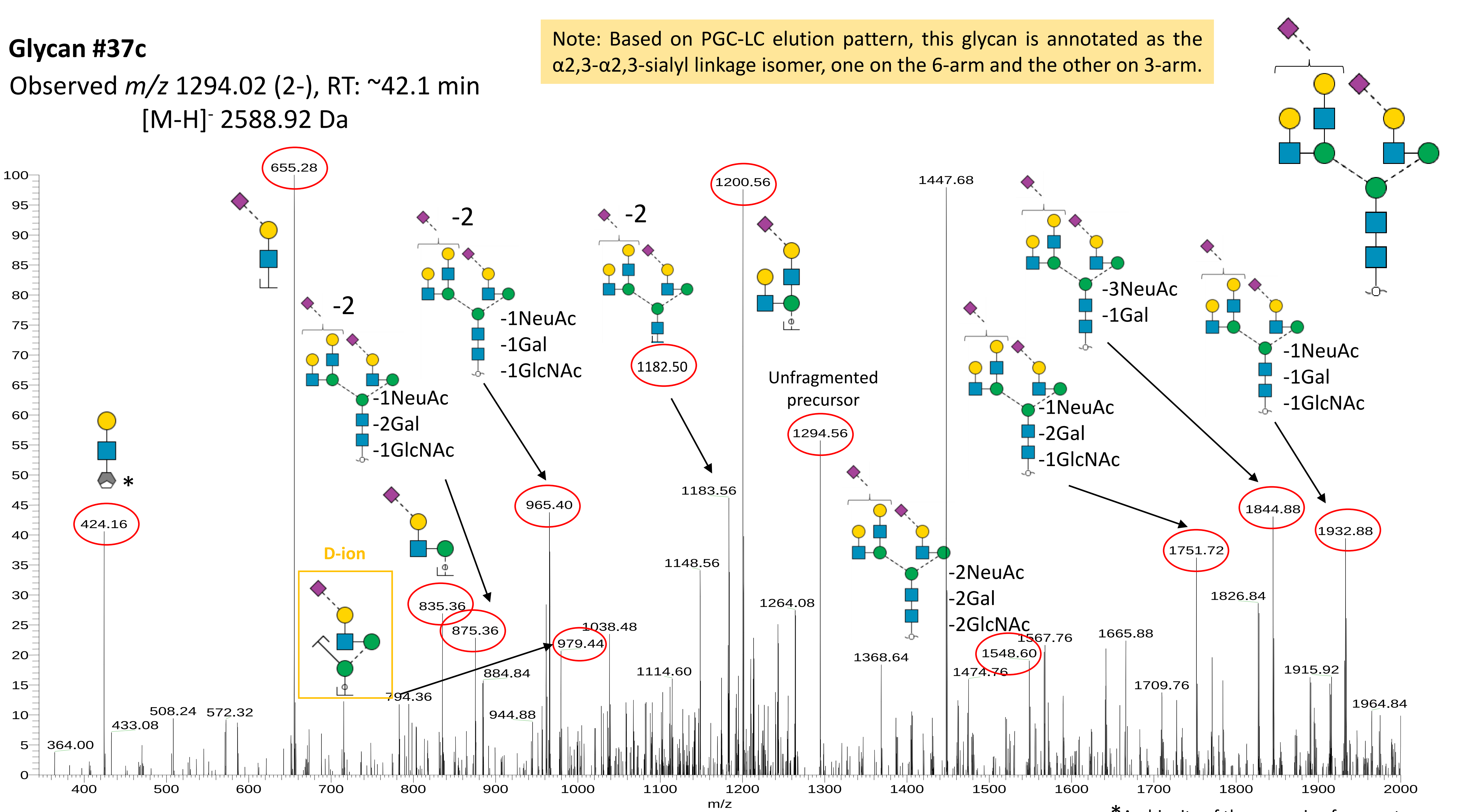

*Ambiguity of the cross-ring fragments

Glycan #37d

Observed m/z 1294.02 (2-), RT: ~43.1 min

$[M-H]^-$ 2588.92 Da

Note: Based on PGC-LC elution pattern, this glycan is annotated as the α2,3-α2,3-sialyl linkage isomer, one on the 6-arm and the other on 3-arm.

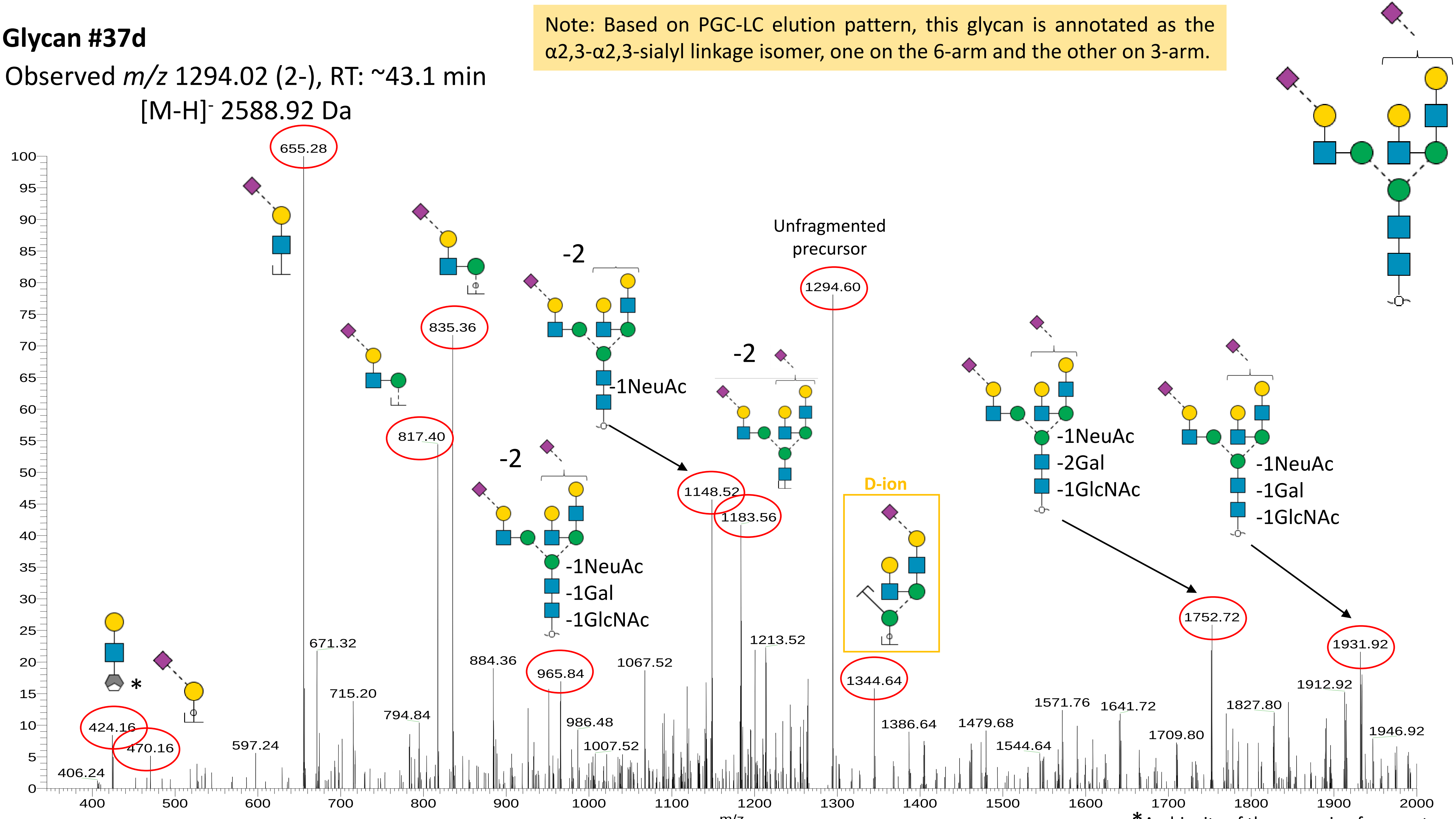

*Ambiguity of the cross-ring fragments

Glycan #38a

Observed *m/z* 1367.06 (2-), RT: ~31.7 min

$[M-H]^-$ 2734.98 Da

Note: Based on PGC-LC elution pattern, this glycan is annotated as the α2,6-α2,6-sialyl linkage isomer but the exact position cannot be determined. Antenna fucose position and linkage also cannot be determined.

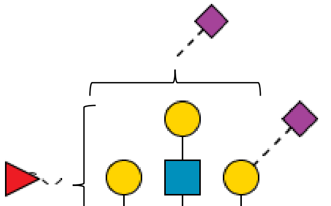

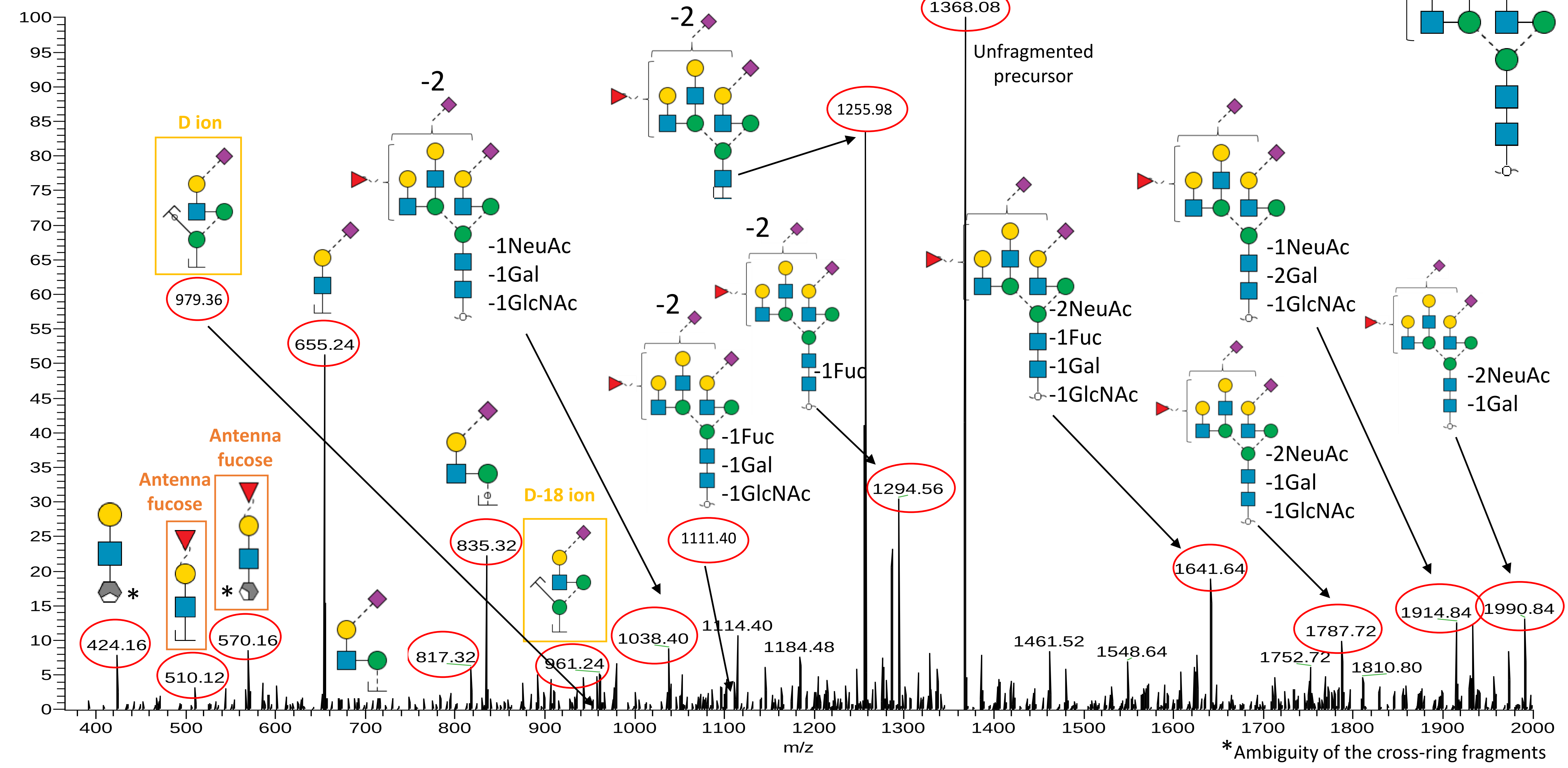

Glycan #38b

Observed *m/z* 1367.06 (2-), RT: ~33.8 min

$[M-H]^-$ 2734.98 Da

Note: Based on PGC-LC elution pattern, this glycan is annotated as the α2,6-α2,3-sialyl linkage isomer but the exact position cannot be determined. Antenna fucose position and linkage also cannot be determined.

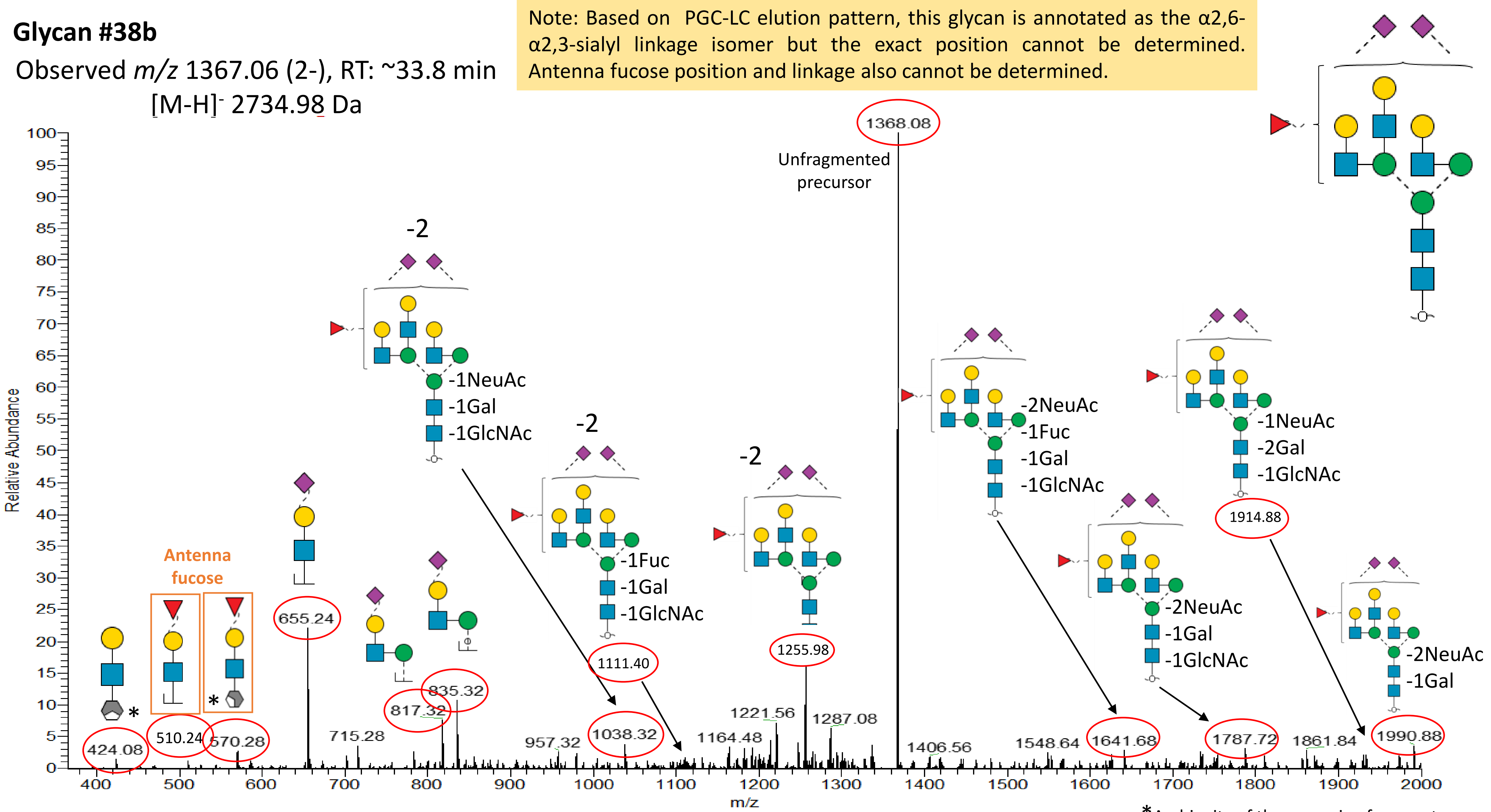

*Ambiguity of the cross-ring fragments

Glycan #39a

Observed *m/z* 1439.56 (2-), RT: ~37.5 min

[M-H]- 2880.00 Da

Note: Based on PGC-LC elution pattern, this glycan is annotated as the α2,6-α2,6-α2,6-sialyl linkage isomer but the exact antennary branching cannot be determined.

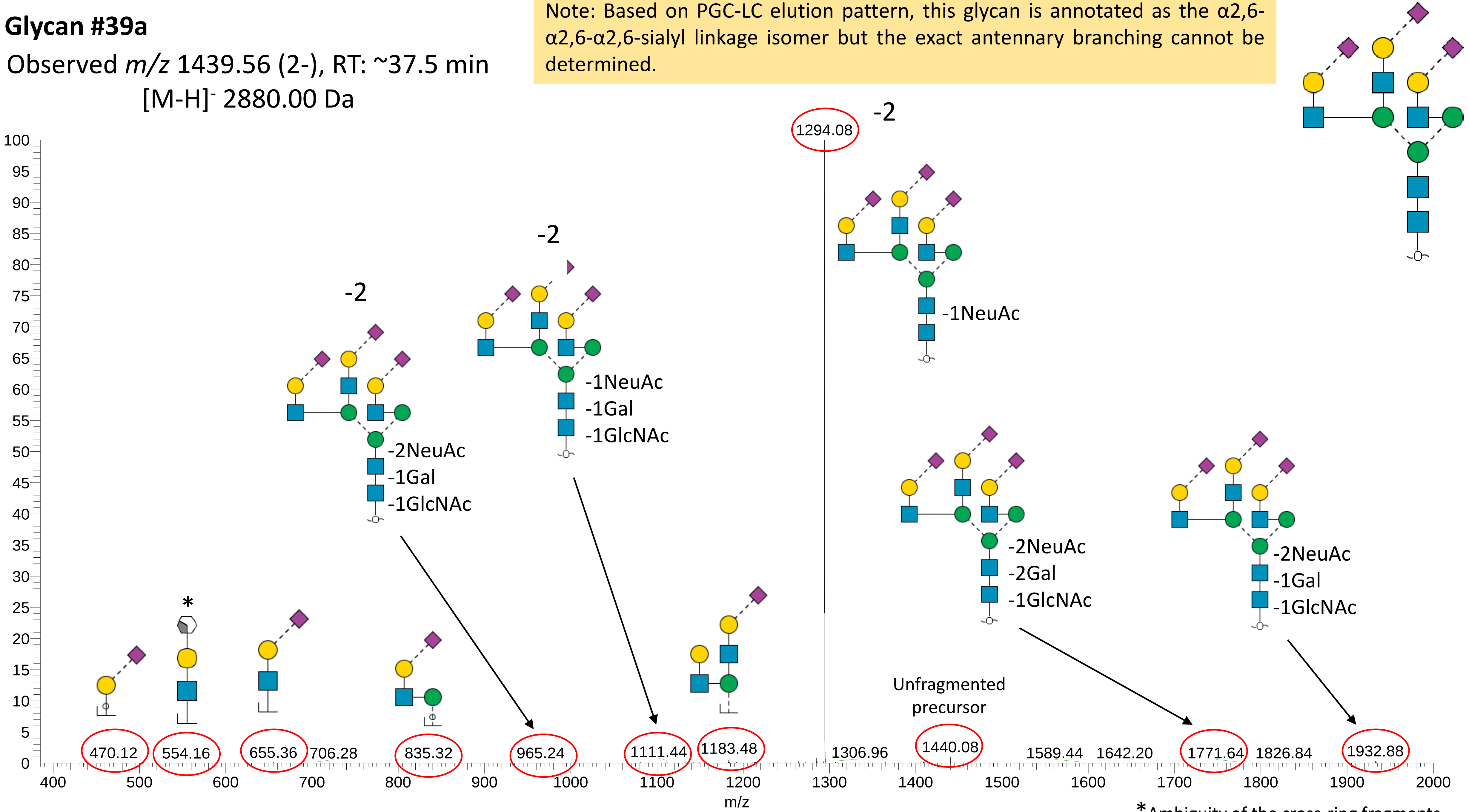

*Ambiguity of the cross-ring fragments

Glycan #39b

Observed *m/z* 1439.56 (2-), RT: ~41.2 min

[M-H]- 2880.00 Da

Note: Based on PGC-LC elution pattern, this glycan is annotated as the α2,6-α2,6-α2,3-sialyl linkage isomer but the exact position and the antennary branching cannot be determined.

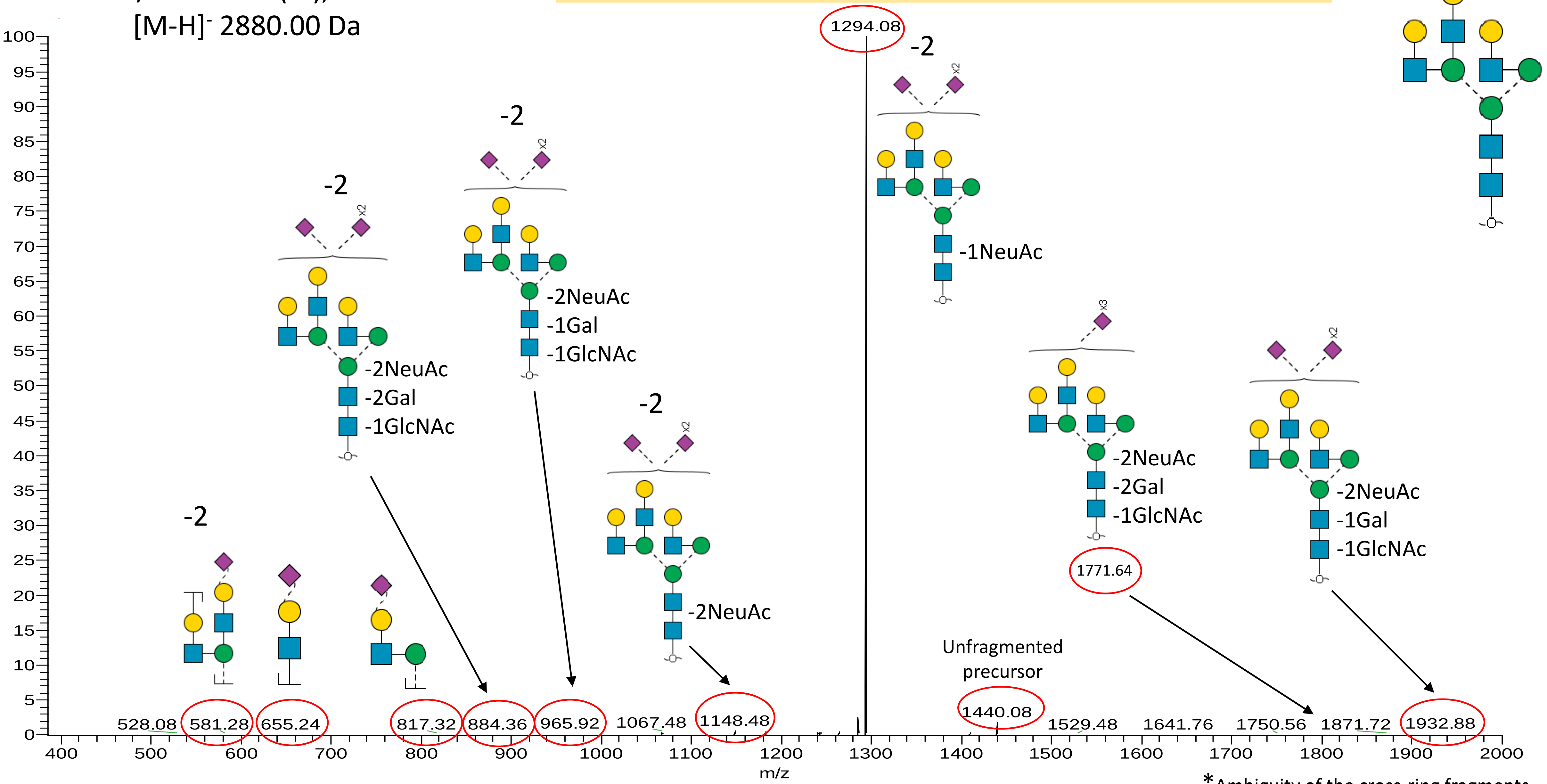

*Ambiguity of the cross-ring fragments

Glycan #39c

Observed *m/z* 1439.56 (2-), RT: ~45.8 min

[M-H]- 2880.00 Da

Note: Based on PGC-LC elution pattern, this glycan is annotated as the α2,6-α2,3-α2,3-sialyl linkage isomer but the exact position and the antennary branching cannot be determined.

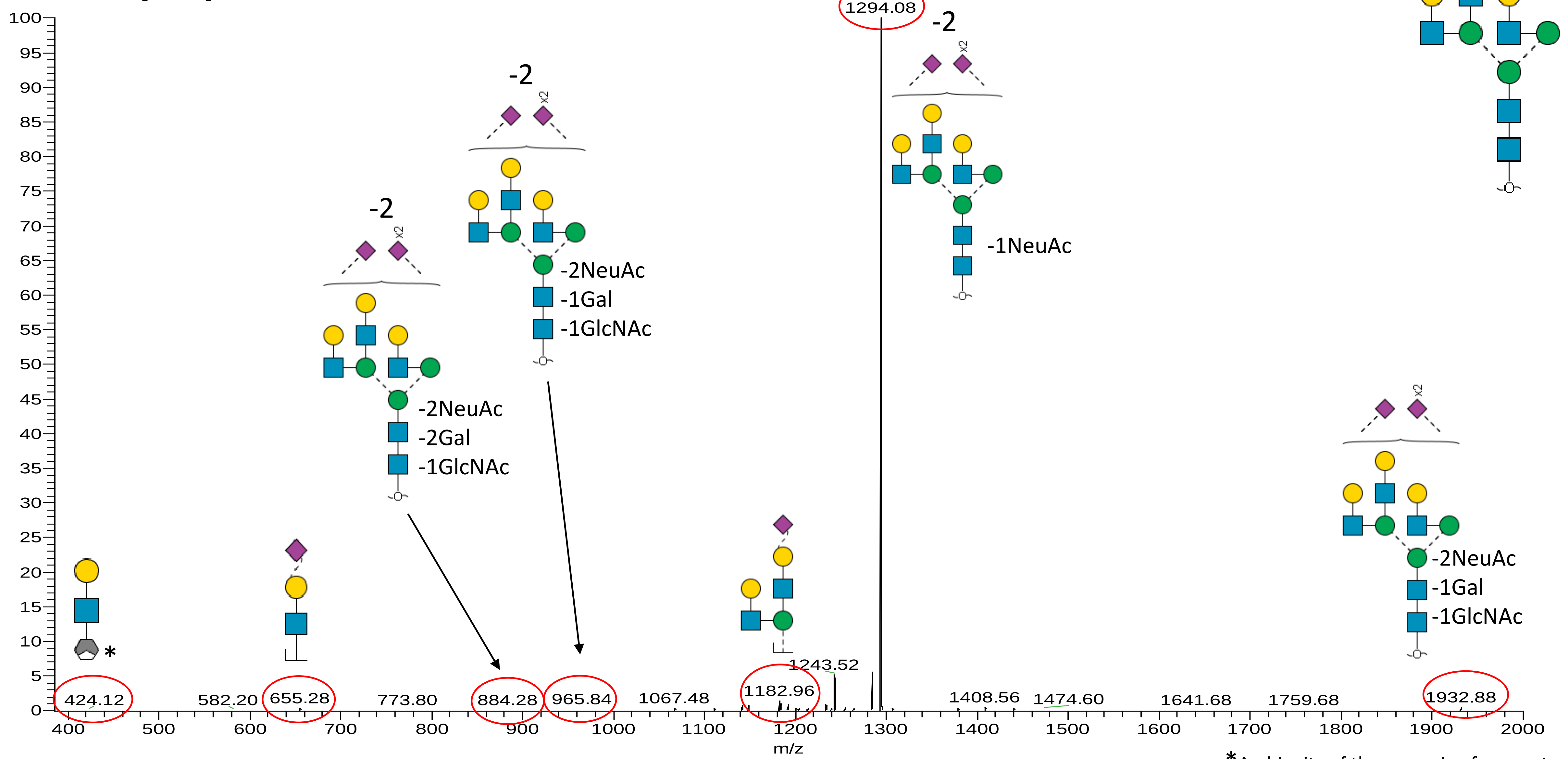

*Ambiguity of the cross-ring fragments

Glycan #39d

Observed *m/z* 1439.50 (2-), RT: ~49.7 min

[M-H]$^-$ 2880.00 Da

Note: Based on PGC-LC elution pattern, this glycan is annotated as the α2,3-α2,3-α2,3-sialyl linkage isomer but the antennary branching cannot be determined.

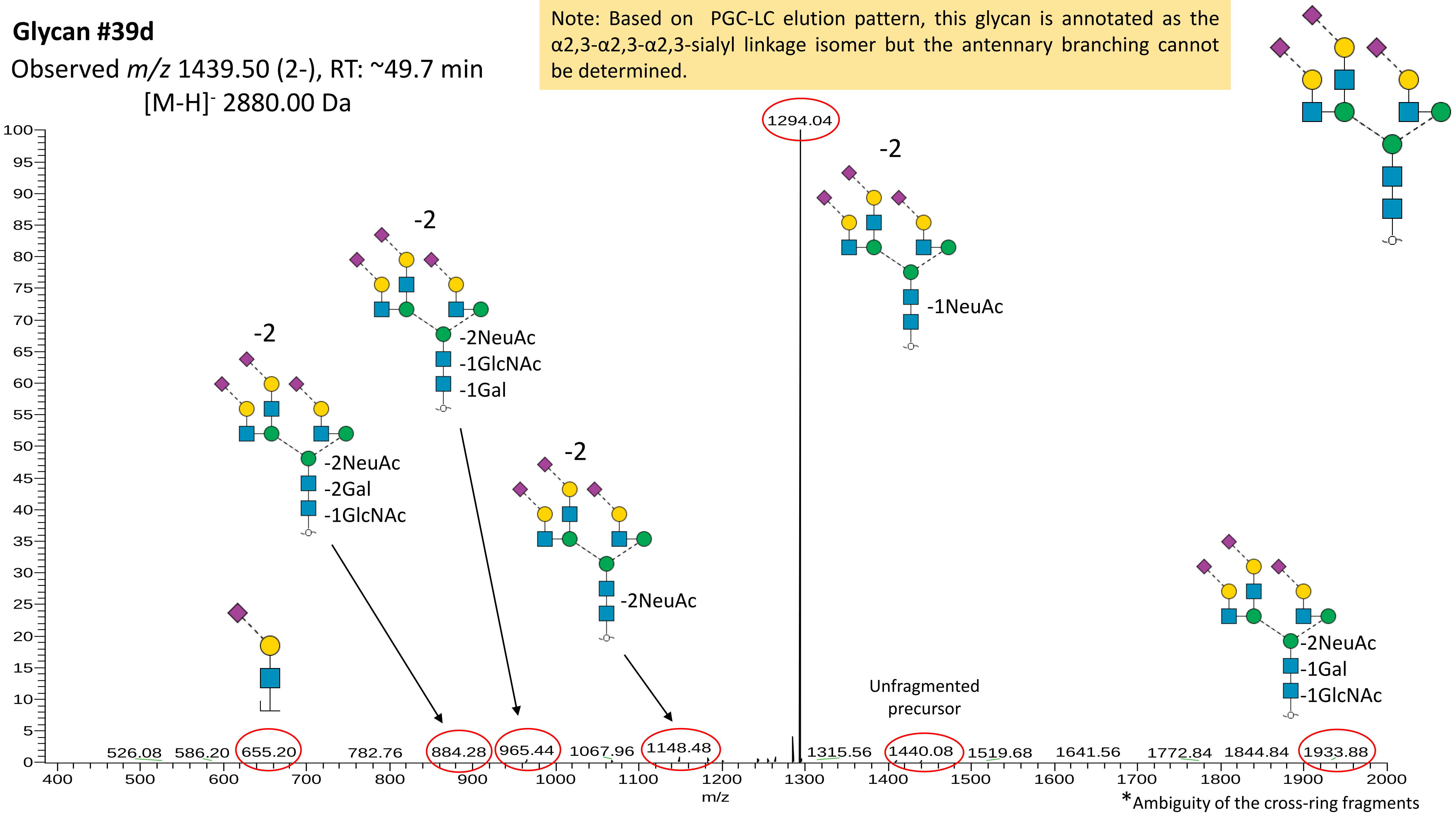

*Ambiguity of the cross-ring fragments

Glycan #40a

Observed *m/z* 1008.0 (3-), RT: ~32.3 min

[M-H]$^-$ 3026.06 Da, [M-2H]$^{2-}$ 1512.5 Da

Note: Based on PGC-LC elution pattern, this glycan is annotated as the α2,6-α2,6-α2,6-sialyl linkage isomer but the antennary branching cannot be determined. Antenna fucose position and linkage also cannot be determined.

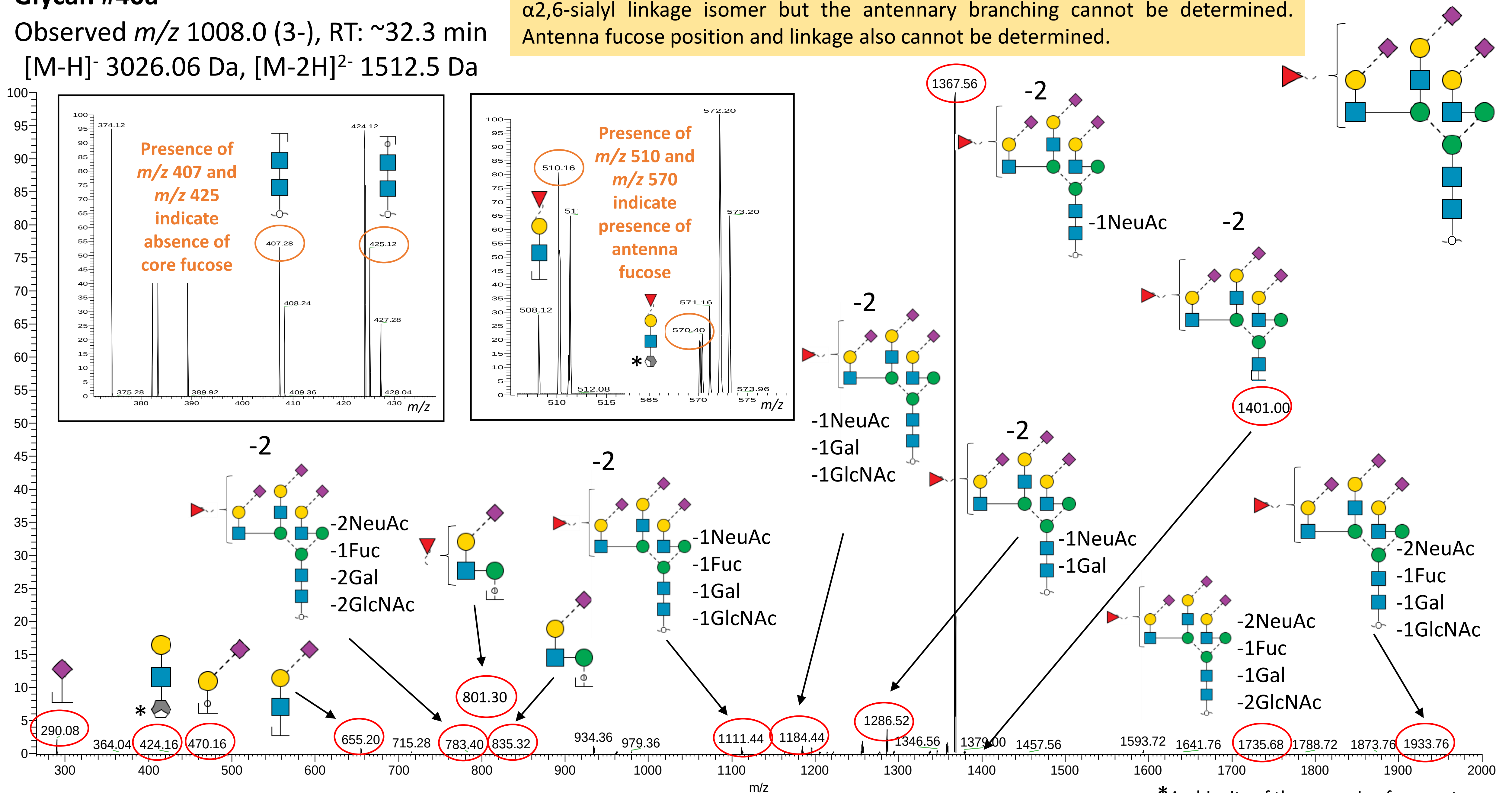

*Ambiguity of the cross-ring fragments

Glycan #40b

Observed *m/z* 1008.0 (3-), RT: ~39.3 min

[M-H]$^-$ 3026.06 Da , [M-2H]$^{2-}$ 1512.5 Da

Note: Based on PGC-LC elution pattern, this glycan is annotated as the α2,6-α2,6-α2,3-sialyl linkage isomer but the exact position and antennary branching cannot be determined. Antenna fucose position and linkage also cannot be determined.

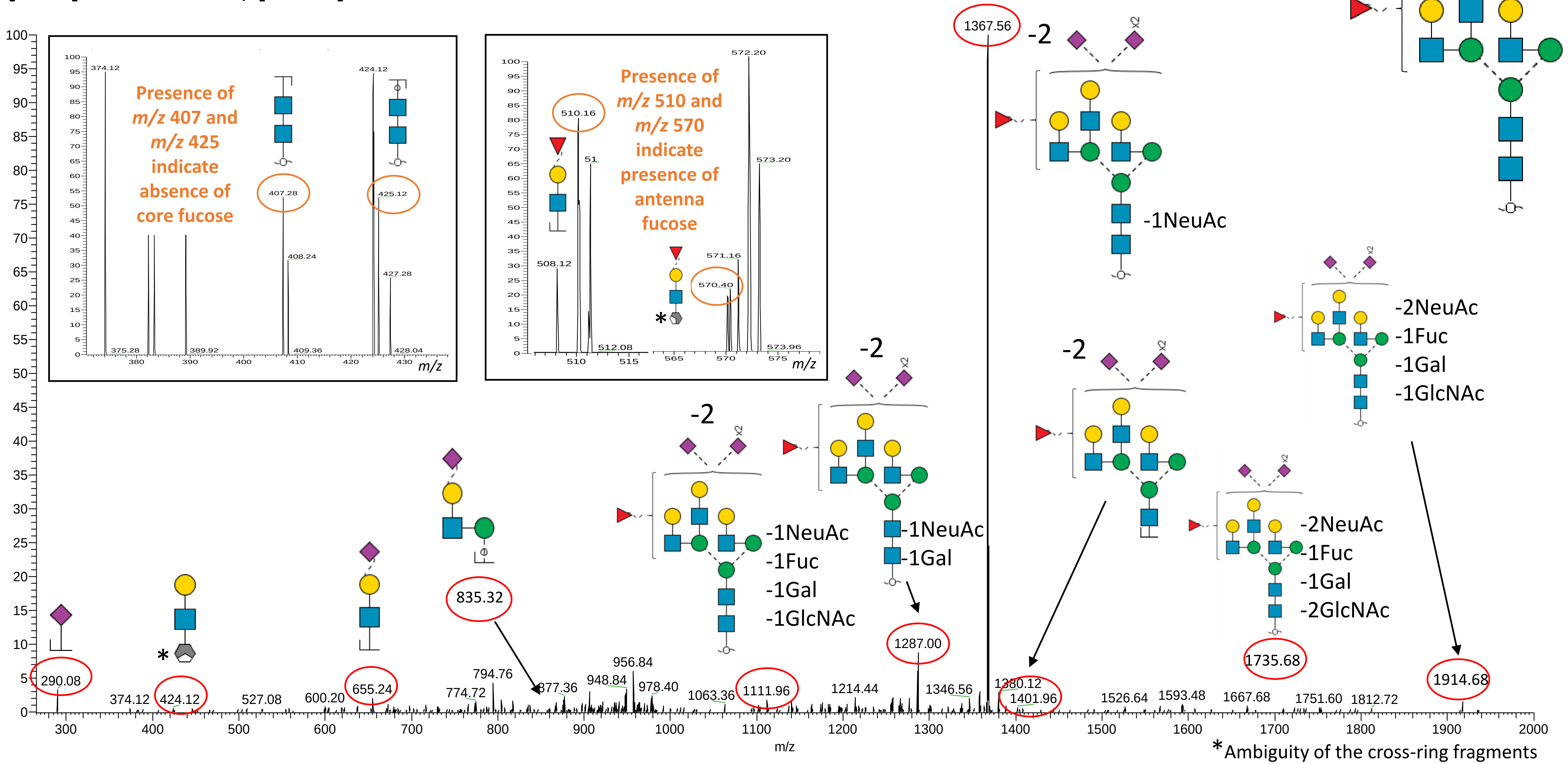

*Ambiguity of the cross-ring fragments

Glycan #40c

Observed *m/z* 1008.0 (3-), RT: ~42.2 min

[M-H]$^-$ 3026.06 Da , [M-2H]$^{2-}$ 1512.5 Da

Note: Based on PGC-LC elution pattern, this glycan is annotated as the α2,6-α2,3-α2,3-sialyl linkage isomer but the exact position and antennary branching cannot be determined. Antenna fucose position and linkage also cannot be determined.

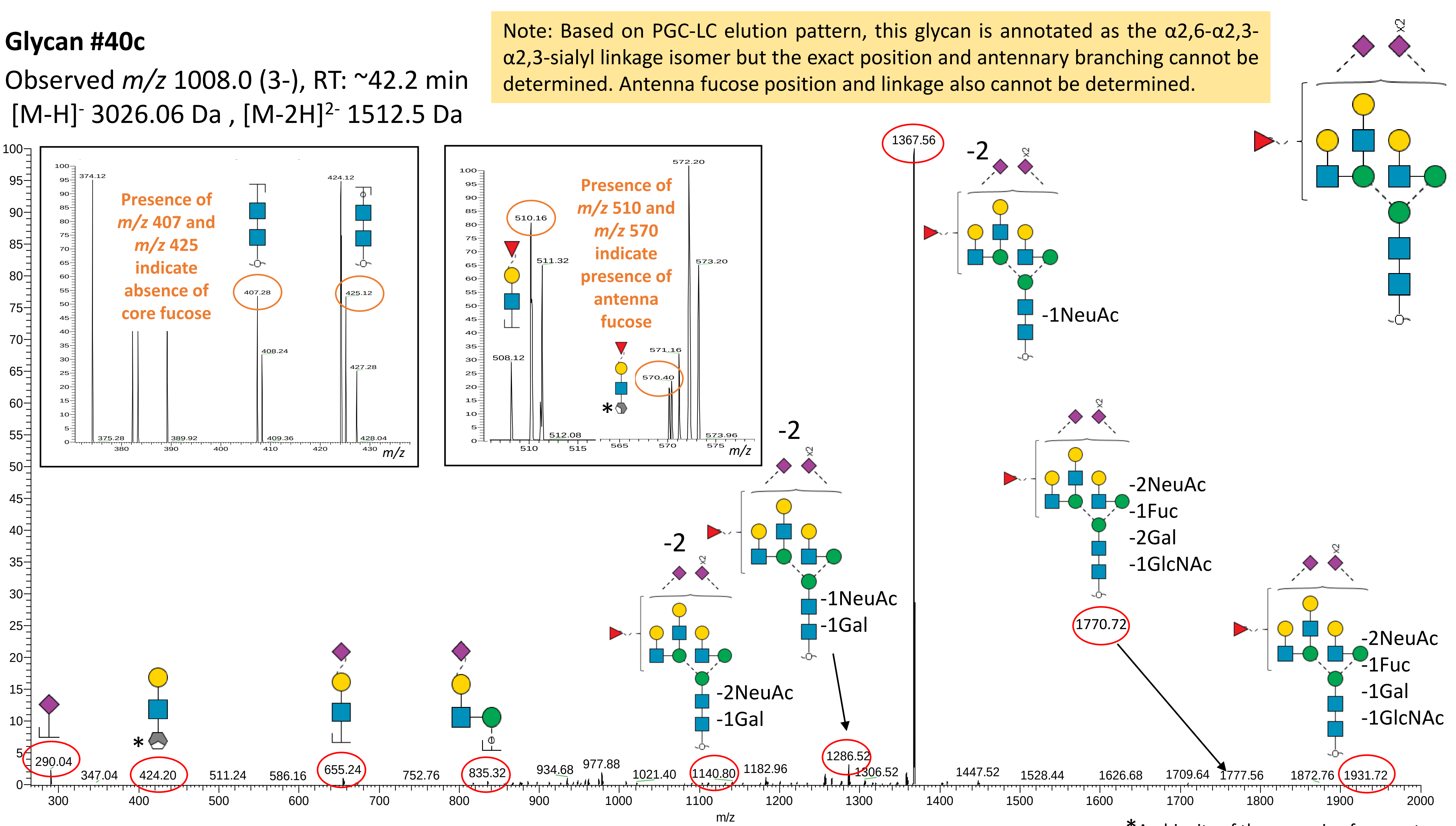

*Ambiguity of the cross-ring fragments

Glycan #40d

Observed *m/z* 1008.0 (3-), RT: ~53.0 min

[M-H]⁻ 3026.06 Da , $[M-2H]^{2-}$ 1512.5 Da

Note: Based on PGC-LC elution pattern, this glycan is annotated as the α2,3-α2,3-α2,3-sialyl linkage isomer but the antennary branching cannot be determined. Antenna fucose position and linkage also cannot be determined.

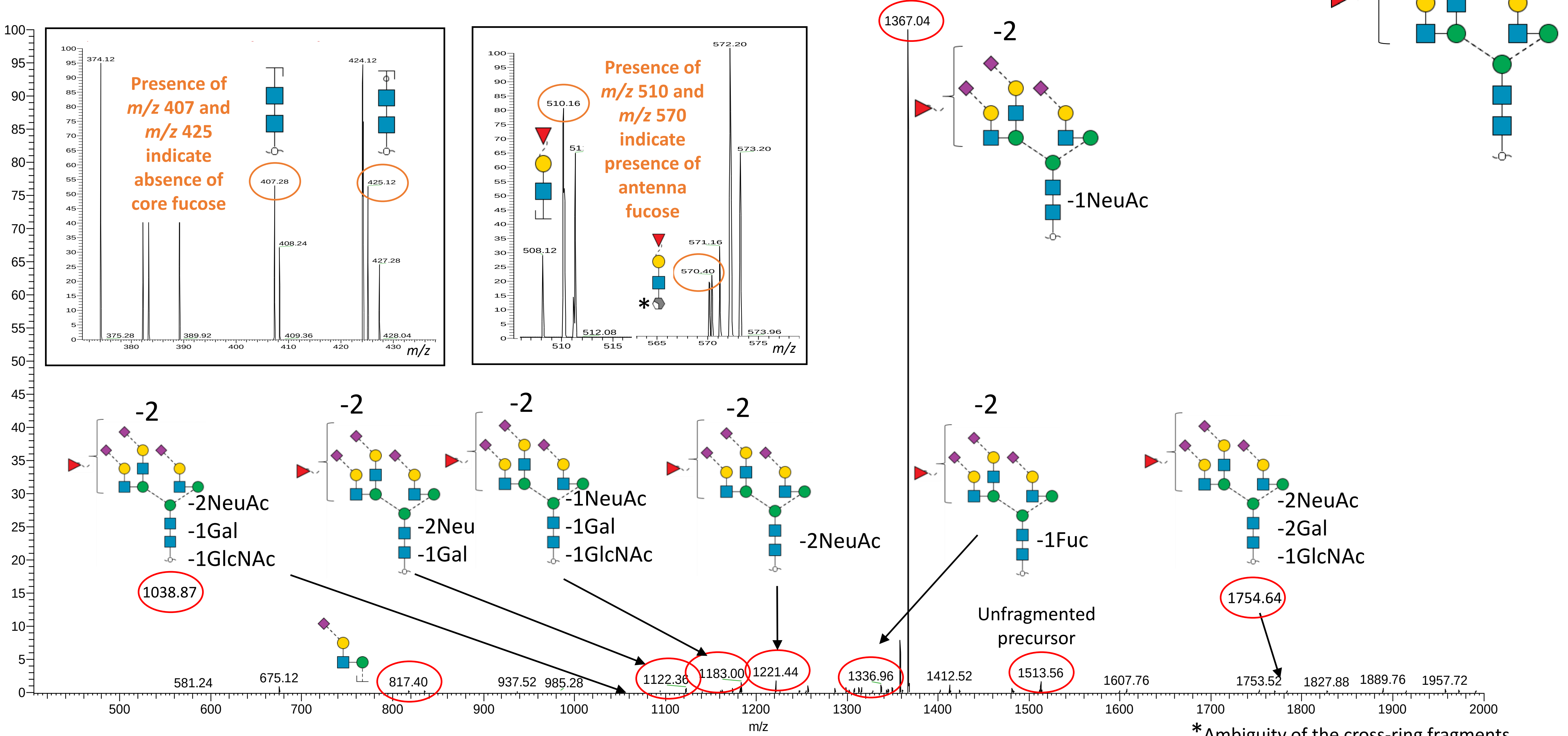

*Ambiguity of the cross-ring fragments

Glycan #41a

Note: This glycan has not been annotated with sialyl linkage isomers and the antenna branching also cannot be determined from the PGC-LC elution pattern.

Observed *m/z* 1178.08 (3-), RT: ~46.2 min

[M-H]- 3536.22 Da , [M-2H]2- 1767.6 Da

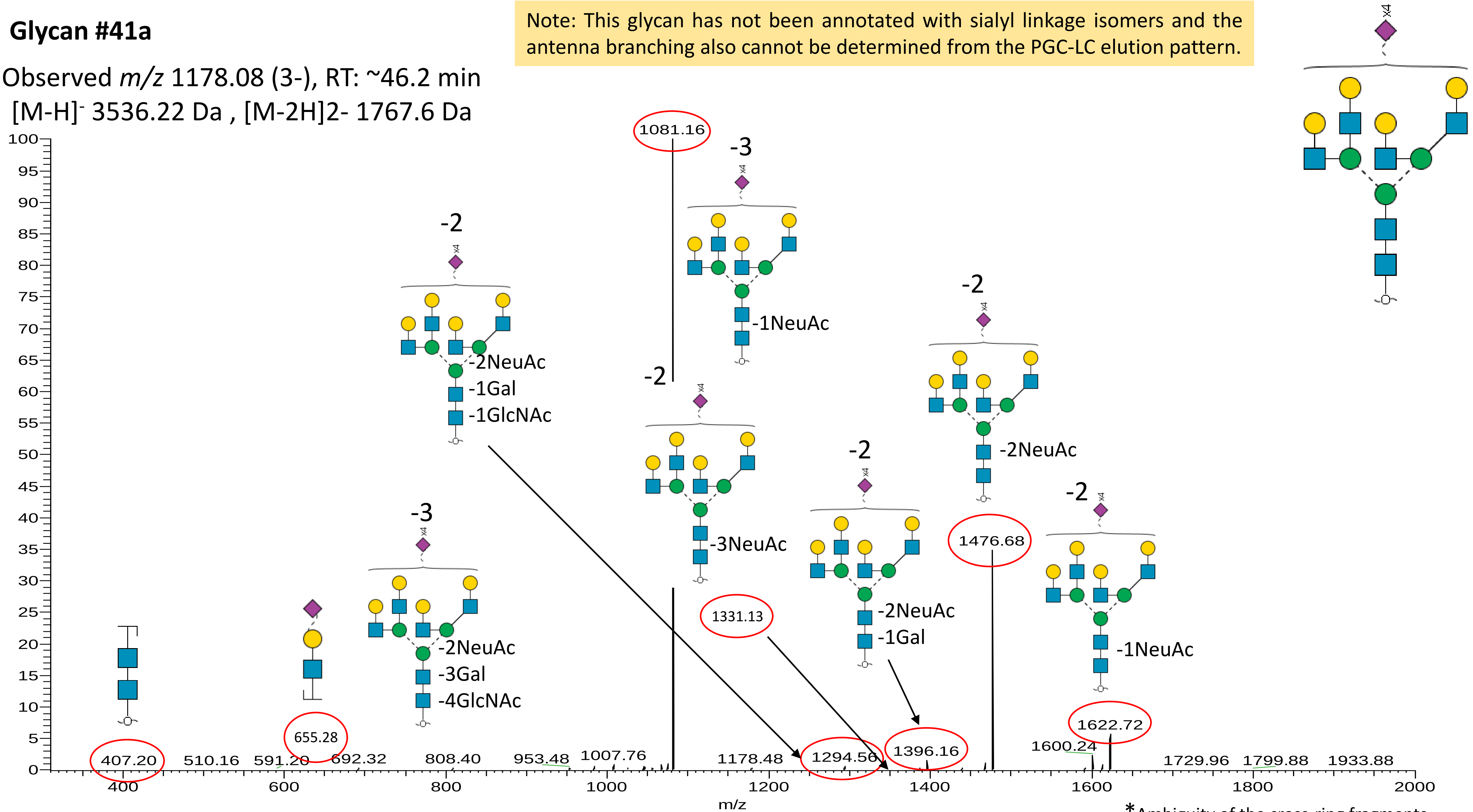

*Ambiguity of the cross-ring fragments

Glycan #41b

Observed *m/z* 1178.08 (3-), RT: ~52.9 min

$[M-H]^-$ 3536.22 Da , [M-2H]2- 1767.6 Da

Note: This glycan has not been annotated with sialyl linkage isomers and the antenna branching also cannot be determined from the PGC-LC elution pattern.

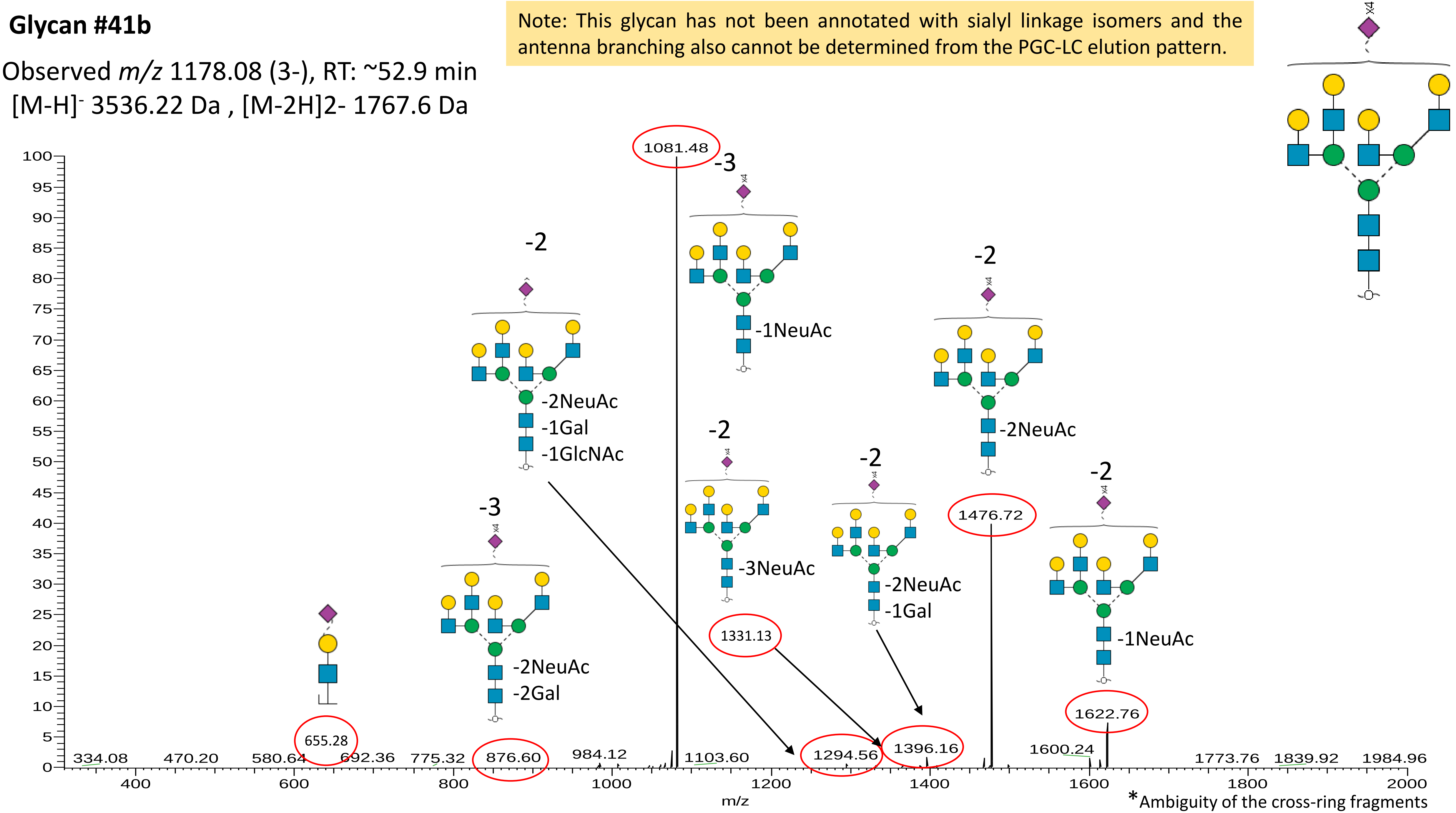

*Ambiguity of the cross-ring fragments

Glycan #41c

Observed *m/z* 1178.08 (3-), RT: ~56.2 min

[M-H]- 3536.22 Da, [M-2H]2- 1767.6 Da

Note: This glycan has not been annotated with sialyl linkage isomers and the antenna branching also cannot be determined from the PGC-LC elution pattern.

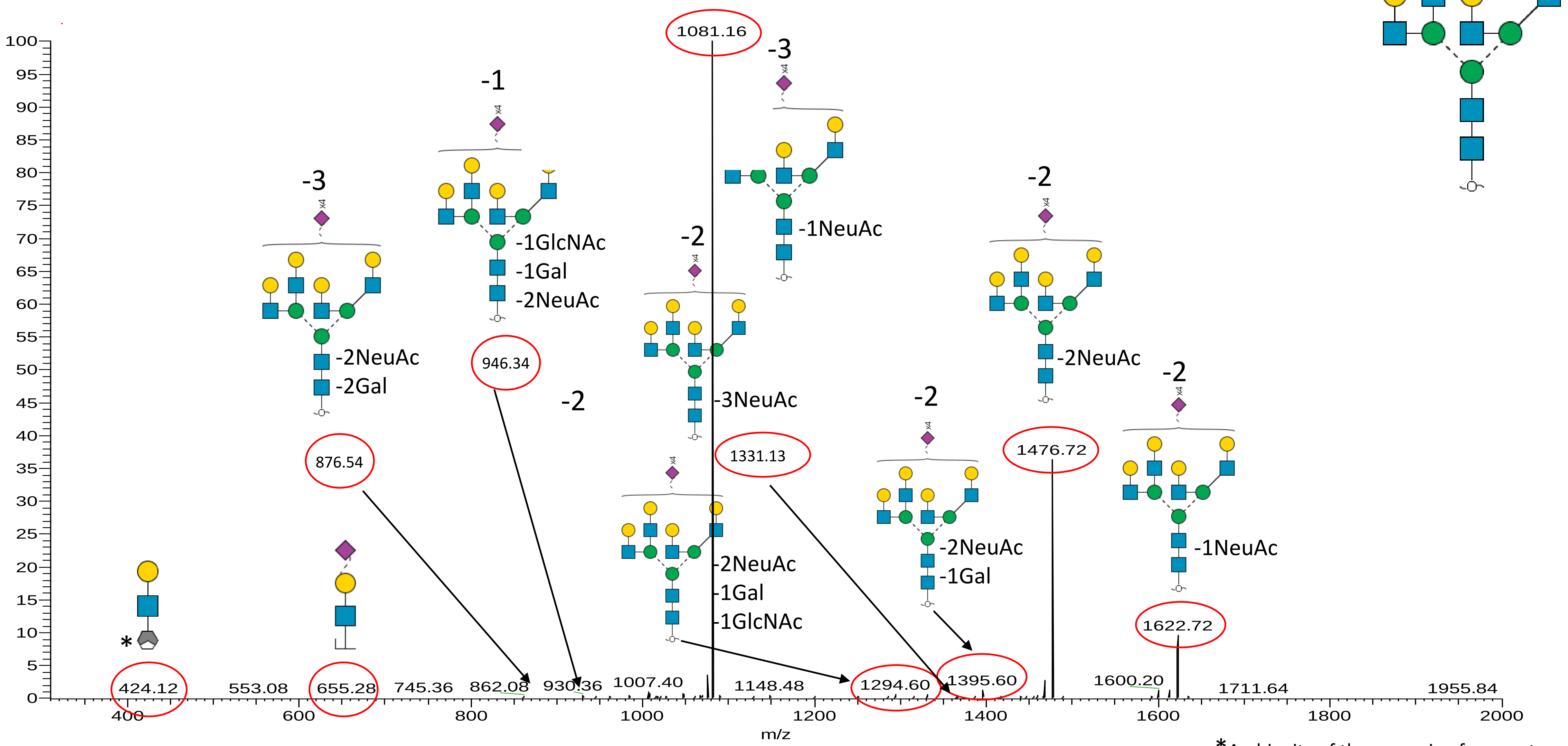

*Ambiguity of the cross-ring fragments
